# Inflammatory proteins associated with Alzheimer’s disease reduced by a GLP1 receptor agonist: a post hoc analysis of the EXSCEL randomized placebo controlled trial

**DOI:** 10.1186/s13195-024-01573-x

**Published:** 2024-10-02

**Authors:** Ivan Koychev, Graham Reid, Maggie Nguyen, Robert J. Mentz, Dan Joyce, Svati H. Shah, Rury R. Holman

**Affiliations:** 1 Department of Psychiatry, Medical Sciences Division, University of Oxford, Oxford, UK; 2 Duke Center for Precision Health, Duke University School of Medicine, Durham, NC USA; 3 Duke Clinical Research Institute, Durham, NC USA; 4 Duke Molecular Physiology Institute, Duke University School of Medicine, Durham, NC USA; 5 Diabetes Trials Unit, Radcliffe Department of Medicine, University of Oxford, Oxford, UK

**Keywords:** Alzheimer’s disease, Glucagon-like peptide-1 receptor agonists, Proteomics

## Abstract

**Background:**

Glucagon-like peptide-1 receptor agonists are a viable option for the prevention of Alzheimer’s disease (AD) but the mechanisms of this potential disease modifying action are unclear. We investigated the effects of once-weekly exenatide (EQW) on AD associated proteomic clusters.

**Methods:**

The Exenatide Study of Cardiovascular Event Lowering study compared the cardiovascular effects of EQW 2 mg with placebo in 13,752 people with type 2 diabetes mellitus. 4,979 proteins were measured (Somascan V0.4) on baseline and 1-year plasma samples of 3,973 participants. C-reactive protein (CRP), ficolin-2 (FCN2), plasminogen activator inhibitor 1 (PAI-1), soluble vascular cell adhesion protein 1 (sVCAM1) and 4 protein clusters were tested in multivariable mixed models.

**Results:**

EQW affected FCN2 (Cohen’s d -0.019), PAI-1 (Cohen’s d -0.033), sVCAM-1 (Cohen’s d 0.035) and a cytokine-cytokine cluster (Cohen’s d 0.037) significantly compared with placebo. These effects were sustained in individuals over the age of 65 but not in those under 65.

**Conclusions:**

EQW treatment was associated with significant change in inflammatory proteins associated with AD.

**Trial Registration:**

EXSCEL is registered on ClinicalTrials.gov: NCT01144338 on 10th of June 2010.

**Supplementary Information:**

The online version contains supplementary material available at 10.1186/s13195-024-01573-x.

## Background

Repurposing existing medications for the prevention of Alzheimer’s disease (AD) is a viable strategy with several promising compounds having been put forward [1]. Among these glucagon-like peptide-1 receptor agonists (GLP-1 RAs), currently marketed for glycaemic control in type 2 diabetes mellitus (T2DM) and for weight loss [2], offer a novel mechanism to modify neurotoxicity in individuals at-risk for AD. GLP-1 RAs are incretins which enhance glucose-dependent insulin secretion, slow gastric emptying, and reduce both postprandial glucagon secretion and food intake [3]. The effect is to reduce post-prandial glycaemia without increasing the risk of hypoglycaemia. Importantly for dementia research, this class of compound has been shown to be anti-inflammatory, reduce cerebrovascular risk [4], to have effects on neural tissue [5, 6]. They can also be given safely in people without diabetes due to the low risk of hypoglycaemia.

Evidence for the potential efficacy of GLP-1 RAs in dementia comes from preclinical research showing that GLP-1 receptors are involved in neurogenesis [7]. Mouse models featuring over-expression of GLP-1 receptors in the hippocampus demonstrated increased neural growth and improved learning [8] while GLP-1 receptor knock-out mice had impaired cognition and evidence for impaired hippocampal function [9].

Pharmaco-epidemiological studies have demonstrated reduced dementia incidence in patients prescribed GLP-1 RAs [10, 11]. A nested case-control study based on dementia diagnosis within a cohort of 176,000 people with T2DM showed that GLP-1 RAs, alongside metformin, dipeptidyl peptidase 4 (DPP4) inhibitors, and sodium-glucose transport protein 2 (SGLT2) inhibitors, are associated with significantly reduced dementia odds ratios after adjusting for demographic and T2DM-related confounders relative to other T2DM therapies: insulin, sulfonylureas and glinides combined, glitazone and acarbose (hazard ratio 0.58, 95% CI 0.50–0.67) [11]. In addition, increasing exposure to GLP-1RAs over time resulted in a further gradual decrease in the risk of dementia. A paper reporting dementia-related outcomes in 15,820 individuals from three placebo-controlled cardiovascular outcome trials (LEADER, PIONEER 6 and SUSTAIN-6), as well as a national healthcare register-based cohort of 120,054 individuals, found strikingly similar results [10]. The authors found a reduced dementia hazard ratio for GLP-1 RAs of 0.47 (95% CI 0.25–0.86) and 0.89 (95% CI 0.86–0.93) in the trial and cohort data respectively. An increase in yearly exposure to GLP-1 RAs was associated with further dementia benefit that primarily affected younger individuals (aged ≤ 70 years), suggesting an age-dependent effect. In a further retrospective cohort study in patients with T2DM [12] we found that 12-month exposure to a GLP-1 RA (semaglutide) was associated with a reduced risk for cognitive deficit compared with sitagliptin (HR 0.72, 95% CI 0.64–0.80; *n* = 23,386) and glipizide (HR 0.72, 95% CI 0.63–0.81; *n* = 19,206), and for new onset dementia compared with sitagliptin (HR 0.52, 95% CI 0.40–0.68).

The existence of legacy biomarker samples from already completed trials testing GLP-1 RAs in other disease areas offer a timely opportunity to further explore the capacity of this class of compounds to alter dementia-related pathophysiology. High dimensional proteomic panels enable extensive examination of biomarker-disease correlates and previous work has shown associations of protein clusters with AD pathology [13, 14]. In one of the largest studies to-date, our group identified 4 protein pathway clusters (metabolic, two cytokine-cytokine receptor interactions, and an undifferentiated one) in a dementia cohort that differentiated individuals with biomarker evidence for AD from controls [15].

In this post-hoc analysis of the Exenatide Study of Cardiovascular Event Lowering (EXSCEL) [16], we sought to provide further evidence for the potential efficacy and mechanism of action in relation to AD of once-weekly exenatide (EQW), a GLP-1 RA, while accounting for the effect of non-modifiable (age) and modifiable (previous cardiovascular events) risk factors. We hypothesized that EQW, compared with placebo, would be associated with significant changes in protein clusters previously shown to be upregulated in AD. We explored subgroup effects based on age and history of previous cardiovascular events based on our hypothesis that GLP-1 RAs may have differential effects in patients with earlier onset of neurodegeneration through its typically more aggressive course [17] and in patients where the neurodegeneration may be cerebrovascular in etiology.

## Methods

### Participants

EXSCEL was a multinational, double-blind, placebo-controlled, randomized trial evaluating the impact of the EQW on CV outcomes in people with T2D [16]. It enrolled 14,752 participants (73.1% with and 26.9% without previous CV disease at 687 sites in 35 countries between June 2010 and September 2015. They were randomized to subcutaneous EQW 2 mg or placebo for 1 year and followed for a median of 3.2 years. Eligible participants were adults with type 2 diabetes (defined as an HbA1c concentration of 6.5–10.0% [48 to 96 mmol/mol]) receiving up to three oral glucose-lowering agents, or insulin alone or with up to two oral glucose-lowering agents. Exclusion criteria included end-stage kidney disease or an estimated glomerular filtration rate (eGFR) of less than 30 ml/ min/1.73 m^2^, high risk for medullary thyroid carcinoma, previous use of GLP-1 RA, or at least two severe hypoglycaemic episodes within the preceding year. While dementia was not an exclusion criterion, participants were required to be able to provide informed consent and thus this minimizes the likelihood of dementia cases at baseline. In addition, we conducted an exploratory natural language processing analysis of adverse event reports (mentions of relevant symptoms, medications, and diagnosis) related to dementia did not uncover statistically significant differences between the randomized groups. The trial protocol was approved by the ethics committee at each participating site (Scotland A Research Ethics Committee in the UK), and all patients provided written informed consent in accordance with the Declaration of Helsinki.

EXSCEL enrolled 14,752 participants (73.1% with and 26.9% without previous CV disease). They were randomized to subcutaneous EQW 2 mg or placebo for 12 months and followed for a median of 3.2 years. Biomarker samples were collected from consenting participants in a subset of sites at baseline and at 1 year (See Supplementary Table 1 for a comparison of overall and biomarker cohorts). Full trial results and CONSORT table are available in the EXSCEL primary results publication [18].

### Proteomic profiling

Plasma proteins were measured using the SomaScan assay platform (SomaLogic Inc.), which uses slow off-rate modified DNA aptamers (SOMAmers) to bind to target proteins and quantify the relative concentrations of proteins. For this study, we used the v.4 assay comprising of 4,979 human proteins SOMAmer reagents mapped to 4776 unique proteins.

For this study, we focused our analysis on 4 proteins and 4 protein clusters that have been shown to be increased in AD(15). The individual proteins were C-reactive protein (CRP), ficolin-2 (FCN2), plasminogen activator inhibitor 1 (PAI-1) and soluble vascular cell adhesion protein 1 (sVCAM-1). There are two aptamer agents targeting FCN2 on the SomaScan assay platform and we included both in our analyses. The protein clusters were two cytokine-cytokine receptor interaction pathways (M2 and M3 pathways), a metabolic pathway (M4 pathway) and an undifferentiated one (M8 pathway). We did not have 100% overlap between the proteins in each cluster and those available in EXSCEL. The percentage of proteins available in EXSCEL for M2, M3, M4, and M8 was 91.2%, 97.7%, 94.8%, and 100%, respectively (See Supplementary Table 2 for a list of included proteins).

### Statistical analyses

Individuals with outlier values, defined as values > 6 median absolute deviations away from the median for either baseline or follow-up, were removed from the analysis of that particular protein. Outlier assessment and removal were performed before calculating cluster scores. All proteins were scaled to a mean 0 and standard deviation 1 distribution before analyses. As we wanted to assess the 1-year change in proteins, we scaled baseline proteins and then used these baseline attributes to scale the 1-year proteins. To create the score, we took the average of the scaled proteins in each cluster. The range of outlier percentage was 0 − 1.37% for our nine endpoints of interest.

Wilcoxon signed rank tests, a non-parametric alternative to the paired t-tests, were used to assess which individual proteins and protein clusters changed between baseline and 1-year. This was done for the entire sample of participants, and in analyses stratified by age (≤ 65 and > 65 years), and by history of prior cardiovascular events (defined as history of a major clinical manifestation of coronary artery disease; ischemic cerebrovascular disease, including history of ischemic stroke or carotid arterial disease; atherosclerotic peripheral arterial disease).

To assess which proteins and protein clusters changed as a function of treatment (EQW or placebo), we fitted three-stage (nested) hierarchical linear mixed-effects models with participant as the random effect. For the first-stage model (Model A), we modelled protein levels as a function of timepoint, treatment, and the interaction between timepoint and treatment. For the second-stage model (Model B), we repeated the base-stage model, while adjusting for age and sex. For the third-stage model (Model C), we repeated the second-stage model, while adjusting for smoking status, systolic blood pressure (SBP), body mass index (BMI), diabetes duration, haemoglobin A1c (HbA1c), baseline estimated glomerular filtration rate (eGFR), and lipids (low density lipoprotein (LDL) cholesterol, high density lipoprotein (HDL) cholesterol, and triglycerides). As an exploratory analysis, we repeated these linear mixed models for the individual proteins included in each protein clusters.

Multiple comparisons were corrected using the Benjamini-Hochberg procedure for the Wilcoxon-signed rank analysis and model A of the linear mixed model analyses, with significance being determined by false discovery rate adjusted p value (FDR p) < 0.1. For all other analyses, significance was determined by nominal *p* < 0.05. All statistical analyses were conducted using R v4.2.1.

## Results

**Fig. 1:**
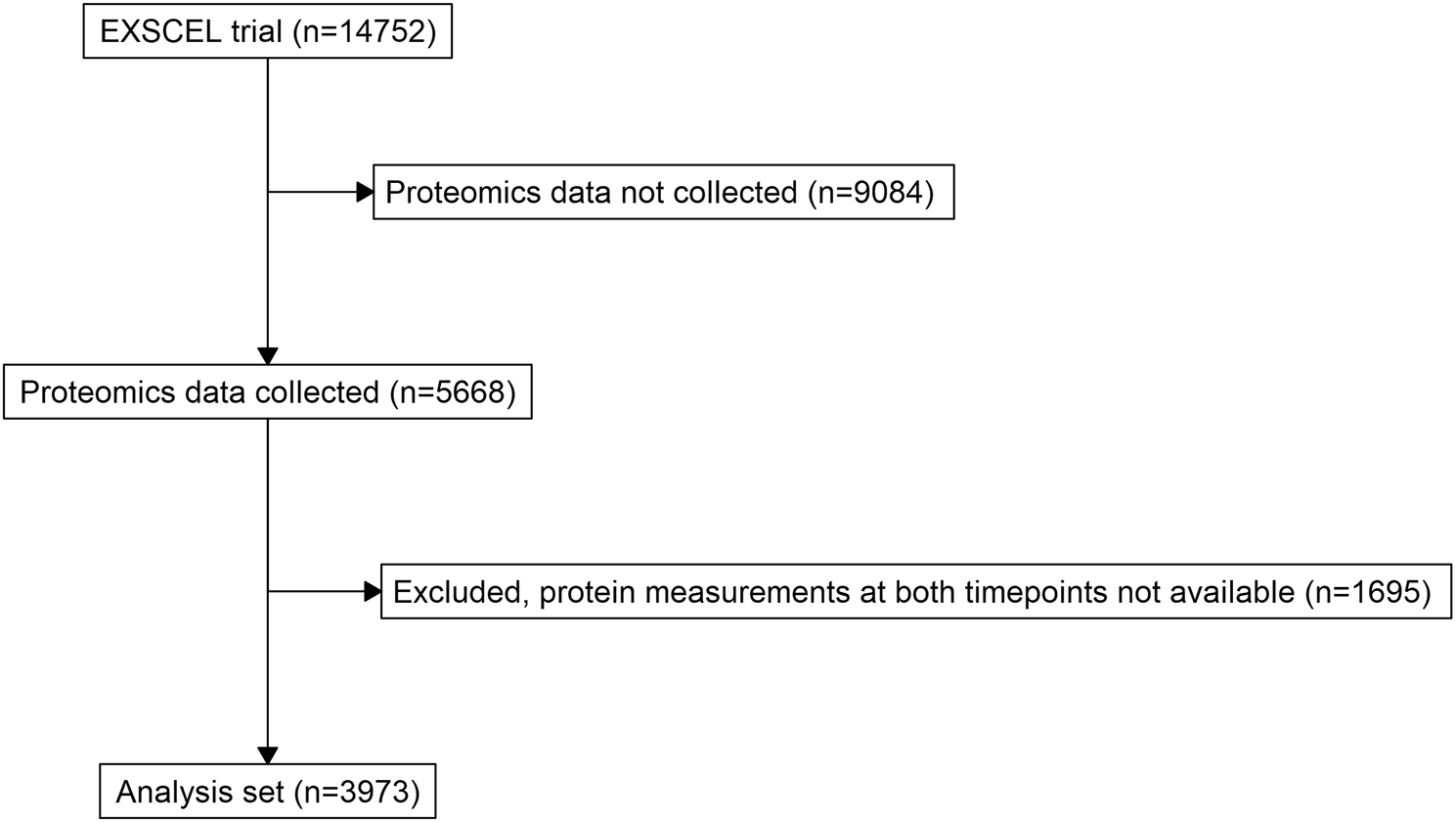
CONSORT diagram of included samples

Of the 5668 EXSCEL participants who provided biomarker samples, we selected 3973 who had baseline and 1-year blood samples (Fig. 1). Table 1 shows the baseline characteristics of the EQW and placebo groups, which did not differ significantly in terms of age, sex, ethnicity, geographic region, smoking status or cardiovascular medical history. From baseline to 1-year, there were small but statistically significant changes in FCN2, PAI-1, sVCAM-1 and M3 score in the overall group of participants. Endpoints that decreased from baseline to 1-year included FCN2 (Cohen’s d -0.019, FDR *p* = 0.035) and PAI-1 (Cohen’s d -0.033, FDR *p* = 0.013); while sVCAM-1 and M3 score increased (Cohen’s d 0.035 and 0.037, FDR *p* = 0.005 and 0.017, respectively) (Table 2). These change directions remained consistent in subsequent risk factor sub-analyses. For participants > 65 years, the same proteins/ clusters were significant (FDR p range 3.0e^− 4^ – 0.07). M2 score significantly increased as well (FDR p 0.07, Cohen’s d 0.059). For participants aged ≤ 65 years, there were no significant changes in proteins after adjustment for multiple comparisons. In participants with prior cardiovascular events, there were significant, but small decrease in FCN2 and increase in sVCAM-1 and M2 scores from baseline to 1-year (FDR-adjusted p range 0.001–0.054, Cohen’s d range − 0.029–0.055). For those without a history of prior cardiovascular events, there was a statistically significant, but small, decrease in PAI-1 from baseline to 1-year (FDR-adjusted p value 0.099, Cohen’s d -0.024).

**Table 1:** Baseline demographics and medical history of EXSCEL trial participants with baseline and 1-year biosamples

			Treatment		
	Overall	Missing	EQW 2 mg	Placebo	P values
n	3973		1994	1979	
Analysis Age (mean (SD))	62.20 (9.40)	0	61.99 (9.48)	62.42 (9.30)	0.146
Sex = M (%)	2404 (60.5)	0	1217 (61.0)	1187 (60.0)	0.518
Pooled Race Group (%)		0			0.709
Asian	251 (6.3)		135 (6.8)	116 (5.9)	
Black	57 (1.4)		26 (1.3)	31 (1.6)	
Hispanic	319 (8.0)		164 (8.2)	155 (7.8)	
Other	17 (0.4)		8 (0.4)	9 (0.5)	
White	3329 (83.8)		1661 (83.3)	1668 (84.3)	
Geographic Region (%)		0			0.736
Asia Pacific	342 (8.6)		180 (9.0)	162 (8.2)	
Europe	2422 (61.0)		1214 (60.9)	1208 (61.0)	
Latin America	304 (7.7)		155 (7.8)	149 (7.5)	
North America	905 (22.8)		445 (22.3)	460 (23.2)	
Smoking Status at BL (%)		0			0.25
Current	412 (10.4)		221 (11.1)	191 (9.7)	
Former	1552 (39.1)		784 (39.3)	768 (38.8)	
Never	2009 (50.6)		989 (49.6)	1020 (51.5)	
Prior CV Event (for stratification) (%)	2821 (71.0)	0	1418 (71.1)	1403 (70.9)	0.907
Duration of Diabetes at BL (mean (SD))	12.93 (8.18)	0.3	12.91 (8.10)	12.96 (8.27)	0.84
Weight at baseline (mean (SD))	95.11 (20.82)	1.8	95.21 (20.79)	95.00 (20.86)	0.753
Body Mass Index at BL (mean (SD))	33.42 (6.30)	1.2	33.37 (6.21)	33.48 (6.40)	0.56
Systolic blood pressure at BL (mean (SD))	135.19 (15.77)	0.1	135.01 (15.67)	135.38 (15.88)	0.46
Diastolic blood pressure at BL (mean (SD))	78.27 (10.12)	0.1	78.48 (10.20)	78.06 (10.03)	0.184
HbA1c at BL (mean (SD))	8.16 (0.94)	0.4	8.17 (0.95)	8.14 (0.93)	0.45
eGFR at BL (mean (SD))	81.32 (20.27)	0.3	81.66 (20.08)	80.99 (20.45)	0.297
History of Heart Failure Flag (%)	841 (21.2)	0	400 (20.1)	441 (22.3)	0.094
History of Cardiovascular Disease Flag (%)	2000 (50.3)	0	996 (49.9)	1004 (50.7)	0.644
Hyperlipidemia (%)	3171 (79.8)	0	1573 (78.9)	1598 (80.7)	0.155
History of Hypertension (%)	3440 (86.6)	0	1716 (86.1)	1724 (87.1)	0.352
History of Cerebrovascular Disease (%)	662 (16.7)	0	330 (16.5)	332 (16.8)	0.882
Hx of Peripheral Arterial Disease (%)	620 (15.6)	0	323 (16.2)	297 (15.0)	0.322
Micro/macro albuminuria at BL (%)	665 (23.3)	28.3	348 (24.5)	317 (22.2)	0.158
Biguanides Therapy at BL (%)	2916 (73.4)	0	1485 (74.5)	1431 (72.3)	0.132
Metformin Therapy at BL (%)	2915 (73.4)	0	1484 (74.5)	1431 (72.3)	0.134
Sulfonylurea Therapy at BL (%)	1424 (35.8)	0	733 (36.8)	691 (34.9)	0.239
Thiazolidinedione Therapy at BL (%)	165 (4.2)	0	83 (4.2)	82 (4.1)	1
Non-sulfonylurea Therapy at BL (%)	46 (1.2)	0	22 (1.1)	24 1.2)	0.862
Alpha-glucosidase Therapy at BL (%)	45 (1.1)	0	27 (1.4)	18 (0.9)	0.24
GLP-1 Analogues Therapy at BL (%)	1 (0.0)	0	0 (0.0)	1 (0.1)	0.997
SGLT-2 Inhibitors Therapy at BL (%)	5 (0.4)	64.2	2 (0.3)	3 (0.4)	1
Insulin Therapy at BL (%)	1881 (47.3)	0	936 (46.9)	945 (47.8)	0.631
Other Therapy at BL (%)	6 (0.4)	64.6	3 (0.4)	3 (0.4)	1
Cholesterol (mg/dl) (mean (SD))	176.99 (52.38)	9.7	175.84 (49.07)	178.14 (55.46)	0.19
HDL (mg/dl) (mean (SD))	44.32 (13.86)	16.9	44.26 (14.75)	44.38 (12.90)	0.804
LDL (mg/dl) (mean (SD))	96.39 (42.23)	23.6	95.71 (40.63)	97.08 (43.77)	0.372
Triglycerides (mg/dl) (mean (SD))	195.33 (140.98)	11.9	195.27 (143.23)	195.39 (138.74)	0.98
Atrial fibrillation/ atrial flutter at BL (%)	334 ( 8.4)	0	167 ( 8.4)	167 ( 8.4)	0.988
Baseline FCN2 (mean (SD))	0.02 (0.99)	0.2	0.01 (1.01)	0.02 (0.97)	0.933
Baseline PAI-1 (mean (SD))	0.02 (0.99)	0	-0.01 (0.87)	0.04 (1.10)	0.118
Baseline VCAM-1 (mean (SD))	-0.02 (0.98)	0	-0.03 (0.99)	-0.01 (0.97)	0.572
Baseline FCN2 (mean (SD))	0.02 (1.00)	0.5	0.01 (1.00)	0.03 (1.00)	0.49
Baseline CRP (mean (SD))	-0.01 (1.00)	0.1	0.00 (1.01)	-0.01 (0.99)	0.89
Baseline Module 2 score (mean (SD))	0.00 (0.17)	0	0.00 (0.17)	-0.01 (0.16)	0.008
Baseline Module 3 score (mean (SD))	0.00 (0.30)	0	-0.01 (0.30)	0.00 (0.31)	0.299
Baseline Module 4 score (mean (SD))	0.00 (0.26)	0	0.00 (0.26)	-0.01 (0.25)	0.462
Baseline Module 8 score (mean (SD))	0.00 (0.30)	0	0.01 (0.30)	-0.01 (0.29)	0.069
Change in FCN2 (mean (SD))	-0.02 (0.56)	0.3	0.00 (0.57)	-0.04 (0.55)	0.026
Change in PAI-1 (mean (SD))	-0.03 (0.85)	0	-0.05 (0.82)	-0.02 (0.88)	0.293
Change in VCAM-1 (mean (SD))	0.04 (0.61)	0	0.01 (0.63)	0.06 (0.59)	0.02
Change in FCN2* (mean (SD))	-0.01 (0.99)	1.3	0.00 (0.98)	-0.02 (1.01)	0.636
Change in CRP (mean (SD))	0.01 (0.87)	0.1	-0.06 (0.89)	0.07 (0.84)	< 0.001
Change in Module 2 score (mean (SD))	0.00 (0.19)	0	0.01 (0.20)	0.00 (0.18)	0.125
Change in Module 3 score (mean (SD))	0.01 (0.23)	0	0.00 (0.23)	0.02 (0.24)	0.005
Change in Module 4 score (mean (SD))	0.00 (0.29)	0	0.00 (0.30)	-0.01 (0.29)	0.172
Change in Module 8 score (mean (SD))	0.00 (0.29)	0	-0.01 (0.29)	0.02 (0.29)	0.018

**Table 2:** Baseline to 1-year changes in proteins and protein clusters, overall, for participants aged ≤ 65 or > 65 years, and for those with or without prior cardiovascular event history

	Overall (*n* = 3973)			Age > 65 (*n* = 1454)			Age ≤ 65 (*n* = 2519)			Prior CV event (*n* = 2821)			No prior CV event (*n* = 1152)		
Target	Mean difference	FDR-adjusted *P* value	Cohen’s d	Mean difference	FDR-adjusted *P* value	Cohen’s d	Mean difference	FDR-adjusted *P* value	Cohen’s d	Mean difference	FDR-adjusted *P* value	Cohen’s d	Mean difference	FDR-adjusted *P* value	Cohen’s d
FCN2	-0.019*	0.035	-0.019	-0.037*	0.022	-0.040	-0.009	0.54	-0.009	-0.028*	0.046	-0.029	0.004	0.71	-0.029
PAI-1	-0.033*	0.013	-0.033	-0.042*	0.070	-0.048	-0.028	0.26	-0.026	-0.025	0.11	-0.024	-0.052*	0.099	-0.024
sVCAM-1	0.035*	0.005	0.035	0.061*	3.0E-04	0.059	0.020	0.48	0.021	0.048*	0.001	0.047	0.004	0.84	0.047
FCN2	-0.010	0.92	-0.010	-0.036	0.60	-0.037	0.004	0.88	0.004	-0.024	0.54	-0.025	0.024	0.52	-0.025
CRP	0.007	0.78	0.007	-0.013	0.59	-0.014	0.018	0.48	0.018	0.027	0.23	0.027	-0.042	0.37	0.027
M2 score	0.005	0.32	0.027	0.010*	0.07	0.058	0.002	0.98	0.009	0.009*	0.054	0.055	-0.007	0.37	0.055
M3 score	0.012*	0.017	0.037	0.014*	0.07	0.044	0.010	0.26	0.035	0.011	0.11	0.035	0.012	0.15	0.035
M4 score	-0.002	0.92	-0.008	0.004	0.59	0.015	-0.006	0.65	-0.022	0.002	0.54	0.009	-0.013	0.37	0.009
M8 score	0.005	0.19	0.016	0.006	0.37	0.021	0.004	0.48	0.013	0.005	0.26	0.015	0.005	0.50	0.015

CRP: C-reactive protein, FCN2: Ficolin-2, PAI-1: Plasminogen activator inhibitor 1, sVCAM1: soluble vascular cell adhesion protein 1, CV: cardiovascular (CV)

* denotes statistically significant results after false discovery rate correction (FDR)

Levels of several endpoints significantly changed differently between participants in the placebo and the once-weekly exenatide group. Across participants mixed-models revealed a significant interaction (FDR p values range 3.9e^− 6^ – 0.061) between treatment and timepoint for several proteins including CRP, sVCAM-1, M3, and M8 whereby they decreased (or increased less) after 1-year EQW treatment compared with placebo (Fig. 1). FCN2 and M2, on the other hand, significantly decreased less (increased more) in the EQW group compared with the placebo group over 1-year. As an outlier sensitivity analysis, we repeated model A as well as the Wilcoxon signed rank test for the individual proteins with all outliers included, and the results did not substantially change (Supplementary Table 3.1 and 3.2).

After adjustment for age and sex only (Model B) these significant interaction effects remained. FCN2, sVCAM-1, CRP as well as M2, M3 and M8 across participants (nominal p 3.3e^− 7^ – 0.044). For model C (adjustment for age, sex, smoking, SBP, DBP, BMI, HbA_1C_, HDL-cholesterol, LDL-cholesterol, triglycerides, diabetes duration, and eGFR), the sample size dropped to 2933 participants due to missing lipid values at baseline. The significant change by treatment in FCN2, sVCAM1, CRP, M3, and M8 remained (nominal p 5e^− 5^ – 0.024, Table 3).

**Table 3:** P values for the interaction term in Model C (adjusted for age, sex, smoking, SBP, DBP, BMI, HbA_1c_, HDL-cholesterol, LDL, triglycerides, diabetes duration, and eGFR), overall, for participants ≤ 65 or > 65 years old, and for those with or without prior cardiovascular events

	Overall	Age > 65	Age ≤ 65	Prior CV	No prior CV
Target	Interaction P statistic	Interaction P statistic	Interaction P statistic	Interaction P statistic	Interaction P statistic
FCN2	0.024*	0.005*	0.524	0.009*	0.955
PAI-1	0.381	0.367	0.077	0.242	0.794
sVCAM-1	0.006*	0.330	0.006*	0.058	0.026*
FCN2	0.781	0.451	0.397	0.848	0.824
CRP	< 0.001*	0.079	< 0.001*	0.001*	0.031*
M2 score	0.146	0.012*	0.925	0.201	0.493
M3 score	0.003*	0.310	0.003*	0.000*	0.752
M4 score	0.102	0.093	0.420	0.245	0.230
M8 score	0.008*	0.466	0.006*	0.006*	0.525

CRP: C-reactive protein, FCN2: Ficolin-2, PAI-1: Plasminogen activator inhibitor 1, sVCAM1: soluble vascular cell adhesion protein 1, CV: cardiovascular (CV)

* denotes statistically significant results (nominal p-value < 0.05)

**Fig. 2:**
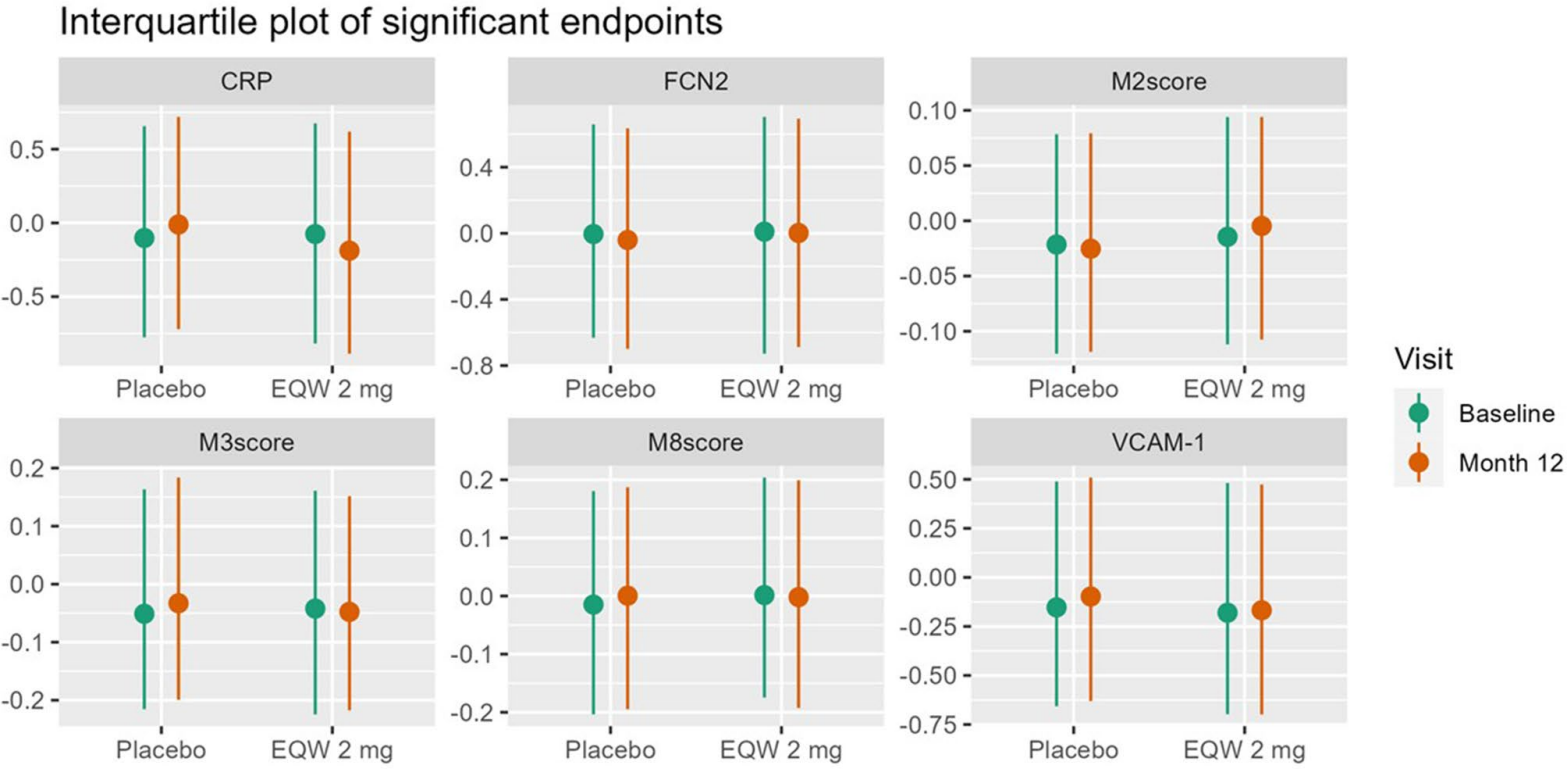
Interquartile plots by visit and treatment of endpoints with a significant interaction term in model A (FDR *p* < 0.1). Abbreviations: C-reactive protein (CRP), Ficolin-2 (FCN2), vascular cell adhesion protein 1 (VCAM1)

In participants older than 65 years, FCN2 and M2 increased in the EQW group compared to placebo, while CRP decreased (FDR p 0.028–0.079). CRP lost significance in the interaction term in our model C adjustment. In participants ≤ 65 years of age, 1-year of EQW treatment compared with placebo associated with decreased levels of PAI-1, sVCAM-1, CRP, M3, and M8 (FDR p 2.3e^− 5^ – 0.088), the last four of which remained significant after model C adjustment (Fig. 2; Table 3).

**Fig. 3:**
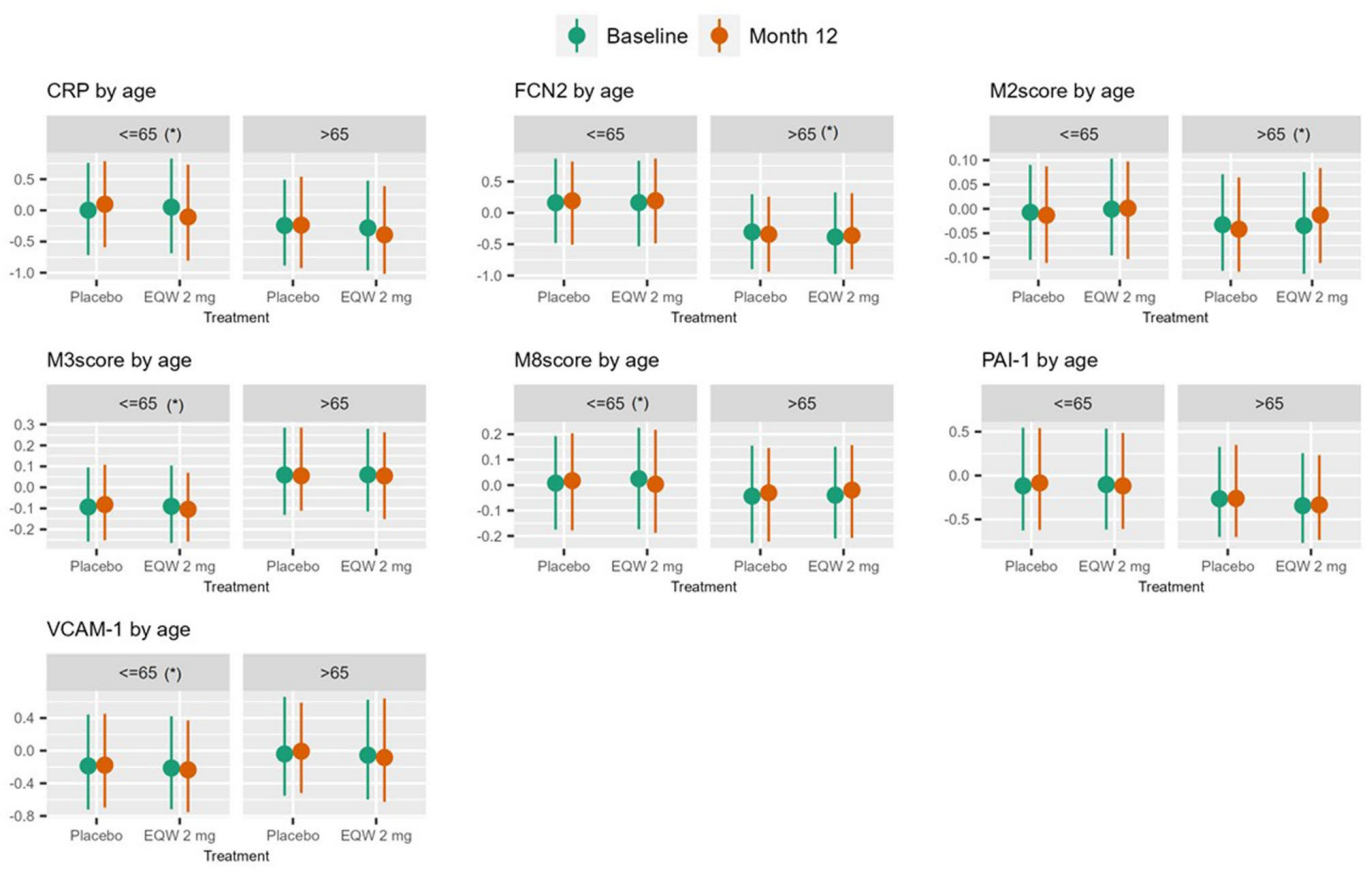
Interquartile plots for endpoints with significant interaction terms in model A in either subgroup. (*) indicates that the interaction term remains significant after adjusting for covariates (model C)

Among those with prior cardiovascular events, 1-year of EQW treatment, compared with placebo, associated with an increase in FCN2 and M2, and a decrease in CRP, M3, and M8 in participants with prior CV events (FDR p 8e^− 4^ – 0.089). These significant interactions remained after model C adjustment, except for M2. In those without history of prior CV events, sVCAM-1 and CRP significantly decreased with EQW treatment throughout models A to C (Fig. 3; Table 3).

**Fig. 4:**
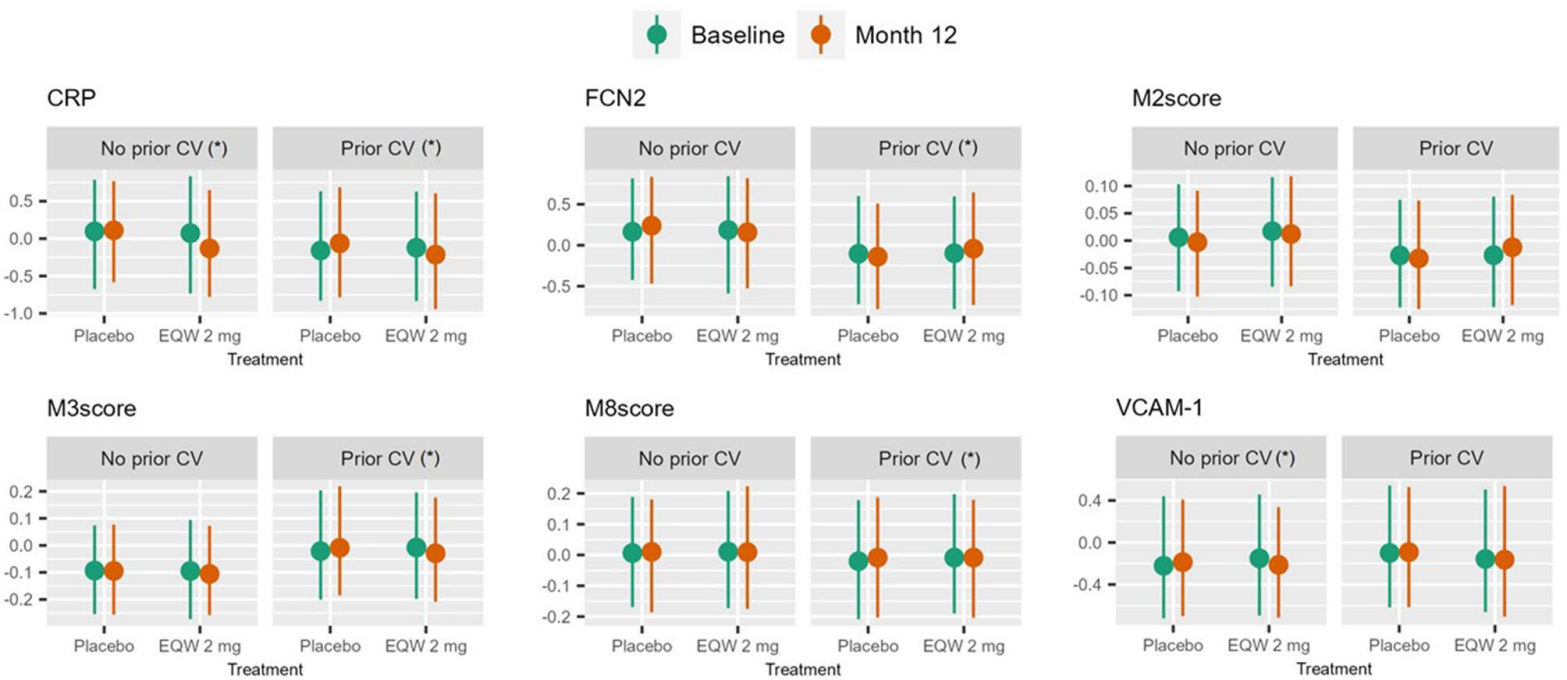
Interquartile plots for endpoints with significant interaction terms in model A in either subgroup. (*) indicates that the interaction term remains significant after adjusting for covariates (model C)

**Fig. 5:**
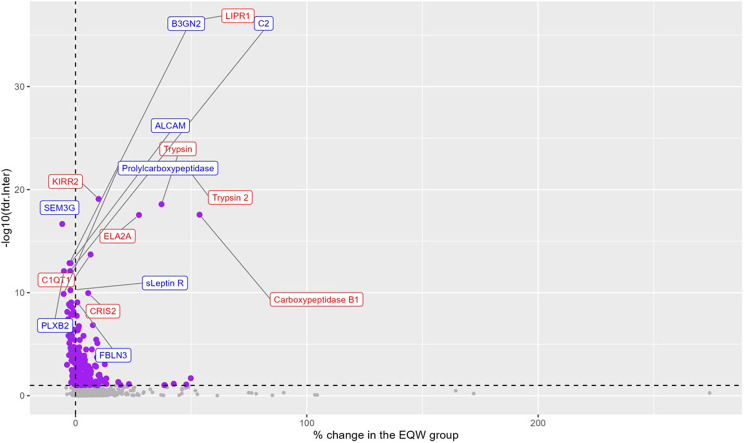
Exploratory analysis for individual proteins included in four clusters. 273/1767 individual proteins included in four clusters change differently from baseline to month 12 with EQW treatment (FDR-adjusted pvalue < 0.1). Model adjusted for age, sex, smoking, systolic blood pressure (BP), diastolic BP, BMI, HBa1C, HDL, LDL, triglycerides, baseline eGFR, and diabetes duration. The x-axis shows the average percentage change in the EQW group. Area to the right of the dashed line indicates increased protein levels in the exenatide group. Top 8 proteins that had average % change in the EQW group greater than and less than the placebo group are labeled in red and blue, respectively

An exploratory analysis of the proteins included in the four protein pathways showed that, after adjusting for covariates (third stage model) and multiple comparisons, 273 proteins changed differentially from baseline to 12 months for the placebo and EQW groups (FDR-adjusted p value of the interaction term < 0.1, Fig. 4). The distribution of those between pathways was 123 in M2, 133 in M3, 28 in M4 and 4 in M8 (some proteins belong to more than one cluster). Supplementary Table S4 presents the top 20 proteins (See Fig. 5).

## Discussion

The aim of this study was to assess the effects of EQW, a GLP-1 RA, on clusters of proteins previously associated with AD using legacy biomarker samples from the EXSCEL trial. We found 1-year of treatment with EQW 2 mg resulted in significant changes to a range of AD-linked inflammatory response proteins, including CRP, sVCAM-1, FCN-2 as well as co-expressed cluster proteins (M3 and M8) scores.

CRP is a hepatic acute-phase reactant that is produced in response to tissue damage, inflammation, and infection. The use of plasma CRP levels as a biomarker for cerebral CRP levels has been validated in previous research showing that CRP in cerebrospinal fluid is correlated with plasma levels of CRP [19]. Looking at the role of CRP in dementia, it has been shown that CRP is upregulated in the brains of AD patients, both at the protein and the mRNA level [20]. Furthermore, studies have demonstrated that CRP is detectable in extracellular amyloid plaques [16], and that it induces the phosphorylation of tau [21]. While some data point to higher levels of CRP predict the progression of normal cognition to dementia [22], the relationship between CRP and dementia risk seems to be moderated by the presence of APOE4 carriership. In a recent study by Tao and colleagues (2021), it was found that CRP was related to longitudinal cognitive decline as measured by Mini-Mental State Examination scores, but only in people who were homozygous for APOE4 [23]. Similarly, in APOE4 homozygotes, but not in other genetic groups, higher levels of CRP were also associated with increased cerebrospinal fluid levels of total and phosphorylated tau [23]. Adding to existing literature showing the efficacy of GLP-1 RAs, such as exenatide, in lowering levels of CRP, we show for the first time that these effects are not age- nor comorbidity- dependent, pointing to the potential scalability and widespread suitability of repurposing of this diabetic drug in lowering systemic inflammation associated with dementia risk.

As for sVCAM-1, it is a cell surface protein that is part of the immunoglobin gene superfamily with evidence of involvement in vascular function. sVCAM-1 is expressed on the surfaces of microvascular endothelial cells and plays a key role in the binding of inflammatory molecules and transmigration of leukocytes to the vascular intima. As such, plasma levels of sVCAM-1 can be taken as a biomarker of endothelial dysfunction. sVCAM-1 has therefore been suggested as a marker of atherosclerosis given its role in vascular remodeling and arterial stiffening [24]. sVCAM-1 has also been shown to associate with impaired cerebrovascular reactivity [25] which has relevance to AD [26]. To this end, VCAM-1 is higher in patients with AD compared to healthy age-matched controls [27] while in initially cognitively healthy adults it was shown to associate with cumulative incidence of cognitive impairment over a 10-year period, independently of age, education, and cardiovascular risk factors [28]. In the current study, 1-year treatment with EQW resulted in reduced levels of VCAM-1, a finding that was seen in participants ≤ 65 years of age and in participants without any history of prior cardiovascular events. This suggests that the beneficial effects of GLP-1 RAs on dementia risk that are mediated through cerebrovascular mechanisms may be more pronounced in individuals without existing vascular pathology.

Whilst the results of our study have shown that 1-year EQW treatment reduces levels of some AD-related inflammatory proteins, our results also showed that EQW treatment increases levels of FCN-2. FCN-2 is a hepatic soluble pattern recognition molecule that can detect pathogen-associated molecular pattern (PAMP) on the surface of foreign particles. Such pattern recognition molecules are part of the innate immune system, functioning as the initial immunological and non-specific response against pathogens. It is known that FCN2, like CRP, functions as an opsonin, encouraging phagocytosis of pathogens when it binds to the carbohydrate, *N*-acetyl-d-glucosamine [29]. Thus, higher FCN2 may be reflective of functioning immune system which contributes to effective clearance of abnormal amyloid and tau. In people across the AD spectrum, FCN2 is increased in those with lower levels of CSF phosphorylated tau [30] and larger entorhinal cortex volumes [14]. In the current study, we found that 12-months of exenatide increase levels of FCN2, but that this finding was most evident in participants over the age of 65 and with a history of previous CV events. These individuals represent the population with the highest burden of modifiable and non-modifiable risk factors for dementia, suggesting that GLP-1 RA effects on regulating the immune system may be particularly relevant in those at highest dementia risk.

It is important to consider the mechanisms through which GLP-1 RAs exerts its anti-inflammatory effects which in turn may have relevance to dementia risk. Studies have shown that GLP-1 RAs reduce the levels of pro-inflammatory cytokines, such as TNF-alpha and IL1-beta [31]. It has been suggested that the anti-inflammatory action of exenatide functions through decreasing TNF-alpha and IL-1Beta and increasing IL-10 by altering the phenotypic behavior of macrophages that excrete inflammatory cytokines [32]. Moreover, GLP-1 has also been shown to upregulate the synthesis of nitric oxide in endothelial cells of the body’ vasculature, including in the umbilical vein [33], as well as in the coronary artery [34]. Not only is nitric oxide one of the body’s main vasodilators that supports circulation and organ perfusion, but nitric oxide also exerts anti-inflammatory effects on the body. Research has shown that nitric oxide indirectly reduces the expression of pro inflammatory cytokines, such as IL-1 and IL-8, and vascular cell adhesion modules by inhibiting NF-KB [35, 36].

The relevance of inflammation to neurodegeneration is substantial with evidence that inflammatory and immunological process may influence the progression and expression of cerebral pathologies [37]. Microglia and astrocytes have been shown to be involved in the immune defense in the parenchyma and dendritic cells can behave like antigen-presenting cells working in tandem with the brain’s vascular system [38, 39]. Astrocytes, whilst performing immunologically in the central nervous system, are also involved in most other cerebral functions that are compromised in neurodegenerative disorders, such as dementia. These include supporting the integrity of the blood brain barrier, neuronal metabolism, synaptogenesis, and the balancing of neurotransmitter levels [40]. Of note, however, is that whilst astrocytic activation can encourage tissue repair, activation can also lead to inflammation and tissue damage within the central nervous system [41]. The hallmark pathologies of AD represent potent triggers of inflammatory responses. That is, amyloid and tau depositions, as well as damaged neurons, are localized and discrete much like localized upregulation of complement, acute phase reactants and cytokines involved in inflammatory processes and the body’s immune system [42]. Neuronal necrosis and apoptosis are, at least in part, attributable to reactive astrocytes that results from amyloid beta activating microglia [43]. GLP-1 RA mediated processed may be a potent mechanism to regulate this process. For example, GLP-1 RA administration downregulates the conversion of astrocytes, inhibits neurodegeneration, and prevents cognitive impairments in animal models of Alzheimer’s disease [44]. Indeed, up to a third of dementia cases are thought to be caused by modifiable risk factors (e.g. diabetes, depression, and obesity) that in turn associate with chronic inflammation [45]. Diabetes has been linked to increased risk for AD through oxidative stress, mitochondrial dysfunction and chronic inflammation. It has been shown that insulin resistance impairs the inhibition of glycogen synthase kinase 3 beta (GSK3β), which is involved in the hyperphosphorylation of tau [46]. GLP-1 RAs, such as exenatide, may thus represents a disease modifying intervention for Alzheimer’s disease, or adjuvant secondary treatment for symptom progression through regulation of chronic inflammation.

### Limitations

Notwithstanding the findings of the current study, a few limitations are worth pointing out. In the first instance, measures of cognitive function and activities of daily living were not captured in the EXSCEL study. Whilst it is promising that the administration of a GLP-1 RA was associated with a reduction in inflammatory biomarkers, many have called for cognitive functioning to be the pivotal assessment of a drug’s efficacy in Alzheimer’s disease trials [47]. This is because other drugs have been shown to alter the cerebral abnormalities associated with dementia without any remediation or slowing of the cognitive deficits. A second important limitation is in the demographic makeup of the original sample in which most participants were Caucasian older adults. Research has shown that non-white adults have higher risk of dementia compared to white adults, necessitating a better understanding of risk and intervention in these groups. After adjustment for age, research has suggested that dementia incidence rates are highest amongst black and Hispanic older adults compared to similar and lower rates for Asian, white and native American older adults [48]. Studies have also shown that modifiable risk factors for dementia differ across ethnic groups and socioeconomic levels. For example, there is variation in the population attributional fraction of dementia across modifiable risk factors, which represents a metric of the proportion of dementia cases in the population that would be avoided if a given risk factor were eliminated. It has been shown that the population attributional fraction of dementia cases caused by modifiable risk factors in lower income countries such as China, India, and areas of Latin America is higher than the estimates usually quoted for higher income countries [49]. This means that the scope for dementia prevention by targeting modifiable risk factors is potentially higher in countries with typically non-Caucasian ethnic backgrounds. Furthermore, there was a high rate of study drug discontinuation [21]. Concomitant, non-T2DM, medications did not differ between groups at baseline [50] but changes over time may theoretically have impacted the proteins studied. Finally, the follow-up period was short in relation to the timescales of AD pathophysiology. However, the fact that we are observing effects on such relative short treatment is promising. Also, in a recently published retrospective cohort study we found that even only a 12-month exposure to another GLP-1 RA associated with reduced risk of cognitive deficits relative to other T2DM medications [12]. Longer-term exposure studies are likely to be required to address this limitation.

Overall, this large study in a sample of ageing T2DM patients has, nonetheless, shown that EQW, a GLP-1 RA, is associated with significant decreases in the levels of inflammatory proteins known to be upregulated in Alzheimer’s disease. In the current paper, we have outlined evidence for the potential role of incretin mimetics in affecting AD risk through inflammatory pathways, potentially pointing to the use of this class of medication in secondary dementia prevention.

## Electronic supplementary material

Below is the link to the electronic supplementary material.

Supplementary Table S1

Supplementary Table S2

Supplementary Table S3

Supplementary Table S4

## Data Availability

Requests for data access and proposals for analyses of EXSCEL data can be submitted to the EXSCEL Publications Committee using the EXSCEL Trial Topic Form found at https://www.rdm.ox.ac.uk/about/our-clinical-facilities-and-units/DTU/completed-trials/exscel.

## Abbreviations

AD: Alzheimer’s disease
BMI: Body mass index
CRP: C-reactive protein
EXSCEL: Exenatide Study of Cardiovascular Event Lowering
FCN2: Ficolin-2
GLP-1 RAs: Glucagon-like peptide-1 receptor agonists
HDL: High density lipoprotein
HbA1c: Haemoglobin A1c
LDL: Low density lipoprotein
EQW: Once-weekly exenatide
PAI-1: Plasminogen activator inhibitor 1
sVCAM1: Soluble vascular cell adhesion protein 1
SBP: Systolic blood pressure
T2D: Type 2 diabetes
