# Supplementary material for: Inflammatory proteins associated with Alzheimer’s disease reduced by a GLP1 receptor agonist: a post hoc analysis of the EXSCEL randomized placebo controlled trial
Source: Alzheimers Res Ther. 2024 Oct 2;16:212. doi: 10.1186/s13195-024-01573-x (PMC11448378; doi:10.1186/s13195-024-01573-x)
Supplement: Supplementary file 1 — Supplementary Table S1 [file 13195_2024_1573_MOESM1_ESM.docx]

|  | **Whole cohort** | | **AD biomarker cohort** | |
| --- | --- | --- | --- | --- |
|  | Overall | Missing | Overall | Missing |
| N | 14752 |  | 3973 |  |
| Analysis Age (mean (SD)) | 61.88 (9.40) | 0 | 62.20 (9.40) | 0 |
| Sex = M (%) | 9149 (62.0) | 0 | 2404 (60.5) | 0 |
| Pooled Race Group (%) |  | 0 |  | 0 |
| Asian | 1452 ( 9.8) |  | 251 ( 6.3) |  |
| Black | 878 ( 6.0) |  | 57 ( 1.4) |  |
| Hispanic | 1134 ( 7.7) |  | 319 ( 8.0) |  |
| Other | 108 ( 0.7) |  | 17 ( 0.4) |  |
| White | 11175 (75.8) |  | 3329 (83.8) |  |
| Geographic Region 1 (%) |  | 0 |  | 0 |
| Asia Pacific | 1529 (10.4) |  | 342 ( 8.6) |  |
| Europe | 6788 (46.0) |  | 2422 (61.0) |  |
| Latin America | 2727 (18.5) |  | 304 ( 7.7) |  |
| North America | 3708 (25.1) |  | 905 (22.8) |  |
| Smoking Status at BL (%) |  | 0 |  | 0 |
| Current | 1721 (11.7) |  | 412 (10.4) |  |
| Former | 5791 (39.3) |  | 1552 (39.1) |  |
| Never | 7233 (49.1) |  | 2009 (50.6) |  |
| Prior CV Event (for stratification) (%) | 10782 (73.1) | 0 | 2821 (71.0) | 0 |
| Duration of Diabetes at BL, yrs (mean (SD)) | 13.08 (8.27) | 0.4 | 12.93 (8.18) | 0.3 |
| Weight at BL, kg (mean (SD)) | 92.33 (21.45) | 0.3 | 95.19 (20.93) | 0.3 |
| Body Mass Index (mean (SD)) | 32.68 (6.40) | 1 | 33.42 (6.30) | 1.2 |
| Systolic BP at BL, mmHg (mean (SD)) | 135.46 (16.88) | 0.2 | 135.19 (15.77) | 0.1 |
| Diastolic BP at BL, mmHg (mean (SD)) | 78.11 (10.27) | 0.2 | 78.27 (10.12) | 0.1 |
| HbA1c at BL (%) (mean (SD)) | 8.12 (0.96) | 0.5 | 8.16 (0.94) | 0.4 |
| History of Heart Failure (%) | 2389 (16.2) | 0 | 841 (21.2) | 0 |
| History of Cardiovascular Disease (%) | 7555 (51.2) | 0 | 2000 (50.3) | 0 |
| Hyperlipidaemia (%) | 11651 (79.0) | 0 | 3171 (79.8) | 0 |
| Hypertension (%) | 12377 (83.9) | 0 | 3440 (86.6) | 0 |
| History of Cerebrovascular Disease (%) | 2450 (16.6) | 0 | 662 (16.7) | 0 |
| History of Peripheral Arterial Disease (%) | 2624 (17.8) | 0 | 620 (15.6) | 0 |
| Micro/macro-albuminuria at BL (%) | 2356 (21.9) | 27 | 665 (23.3) | 28.3 |
| Biguanides Therapy at BL (%) | 11295 (76.6) | 0 | 2916 (73.4) | 0 |
| Metformin Therapy at BL (%) | 11294 (76.6) | 0 | 2915 (73.4) | 0 |
| Sulfonylurea Therapy at BL (%) | 5401 (36.6) | 0 | 1424 (35.8) | 0 |
| Thiazolidinedione Therapy at BL (%) | 579 (3.9) | 0 | 165 (4.2) | 0 |
| Non-sulfonylurea Therapy at BL (%) | 202 (1.4) | 0 | 46 (1.2) | 0 |
| Alpha-glucosidase Therapy at BL (%) | 300 (2.0) | 0 | 45 (1.1) | 0 |
| GLP-1 Analogues Therapy at BL (%) | 2 (0.0) | 0 | 1 (0.0) | 0 |
| SGLT-2 Inhibitors Therapy at BL (%) | 77 (0.9) | 42.1 | 5 (0.4) | 64.2 |
| Insulin Therapy at BL (%) | 6836 (46.3) | 0 | 1881 (47.3) | 0 |
| Other Therapy at BL (%) | 58 (0.7) | 42.6 | 6 (0.4) | 64.6 |
| Cholesterol, mg/dl (mean (SD)) | 175.32 (126.98) | 12.8 | 176.99 (52.38) | 9.7 |
| HDL, mg/dl (mean (SD)) | 44.00 (25.51) | 18.1 | 44.32 (13.86) | 16.9 |
| LDL, mg/dl (mean (SD)) | 95.02 (55.89) | 23.5 | 96.39 (42.23) | 23.6 |
| Triglycerides, mg/dl (mean (SD)) | 197.52 (343.57) | 14.1 | 195.33 (140.98) | 11.9 |
| Atrial fibrillation/atrial flutter (%) | 999 (6.8) | 0 | 334 (8.4) | 0 |
