# Supplementary material for: Inflammatory proteins associated with Alzheimer’s disease reduced by a GLP1 receptor agonist: a post hoc analysis of the EXSCEL randomized placebo controlled trial
Source: Alzheimers Res Ther. 2024 Oct 2;16:212. doi: 10.1186/s13195-024-01573-x (PMC11448378; doi:10.1186/s13195-024-01573-x)
Supplement: Supplementary file 2 — Supplementary Table S2 [file 13195_2024_1573_MOESM2_ESM.docx]

Table S2: List of proteins available in EXSCEL per pathway.

| **UniProt** | **SeqID1** | **Target Full Name** | **Target** | **Module** |
| --- | --- | --- | --- | --- |
| Q01968 | SL10011_65 | Inositol polyphosphate 5-phosphatase OCRL-1 | OCRL | M2 |
| O43623 | SL10014_31 | Zinc finger protein SNAI2 | SLUG | M2 |
| P11473 | SL10023_32 | Vitamin D3 receptor | VDR | M2 |
| Q86XE5 | SL10024_44 | 4-hydroxy-2-oxoglutarate aldolase, mitochondrial | HOGA1 | M2 |
| Q13115 | SL10035_6 | Dual specificity protein phosphatase 4 | DUS4 | M2 |
| Q96PQ1 | SL10037_98 | Sialic acid-binding Ig-like lectin 12 | SIG12 | M2 |
| P19878 | SL10047_12 | Neutrophil cytosol factor 2 | NCF-2 | M2 |
| P54274 | SL10049_112 | Telomeric repeat-binding factor 1 | TERF1 | M2 |
| Q9NW38 | SL10063_10 | E3 ubiquitin-protein ligase FANCL | FANCL | M2 |
| O95817 | SL10078_5 | BAG family molecular chaperone regulator 3 | BAG3 | M2 |
| Q9UPW6 | SL10081_17 | DNA-binding protein SATB2 | SATB2 | M2 |
| P49675 | SL10085_25 | Steroidogenic acute regulatory protein, mitochondrial | STAR | M2 |
| P35520 | SL10086_39 | Cystathionine beta-synthase | CBS | M2 |
| P02489 | SL10087_10 | Alpha-crystallin A chain | Alpha crystallin A chain | M2 |
| Q8N2W9 | SL10342_55 | E3 SUMO-protein ligase PIAS4 | PIAS4 | M2 |
| Q13651 | SL10344_334 | Interleukin-10 receptor subunit alpha | IL-10 Ra | M2 |
| P05412 | SL10356_21 | Transcription factor AP-1 | c-Jun | M2 |
| Q9ULT6 | SL10390_21 | E3 ubiquitin-protein ligase ZNRF3 | ZNRF3 | M2 |
| O43286 | SL10425_3 | Beta-1,4-galactosyltransferase 5 | B4GT5 | M2 |
| P15509 | SL10438_19 | Granulocyte-macrophage colony-stimulating factor receptor subunit alpha | CSF2R | M2 |
| Q9H6B4 | SL10440_26 | CXADR-like membrane protein | ACAM | M2 |
| Q02818 | SL10451_11 | Nucleobindin-1 | NUCB1 | M2 |
| Q6UY09 | SL10453_7 | Carcinoembryonic antigen-related cell adhesion molecule 20 | CEA20 | M2 |
| Q9Y5Q6 | SL10462_14 | Insulin-like peptide INSL5 | INSL5 | M2 |
| O15173 | SL10506_53 | Membrane-associated progesterone receptor component 2 | PGRC2 | M2 |
| P32927 | SL10512_13 | Cytokine receptor common subunit beta | IL3RB | M2 |
| Q9BRK3 | SL10521_10 | Matrix-remodeling-associated protein 8 | MXRA8 | M2 |
| Q08211 | SL10527_22 | ATP-dependent RNA helicase A | DHX9 | M2 |
| Q8NFQ8 | SL10553_8 | Torsin-1A-interacting protein 2 | TOIP2 | M2 |
| Q8N6K0 | SL10557_6 | Testis-expressed sequence 29 protein | TEX29 | M2 |
| Q9NQX7 | SL10560_1 | Integral membrane protein 2C | ITM2C | M2 |
| Q7Z3D4 | SL10563_13 | LysM and putative peptidoglycan-binding domain-containing protein 3 | LYSM3 | M2 |
| O94933 | SL10565_19 | SLIT and NTRK-like protein 3 | SLIK3 | M2 |
| O60676 | SL10572_65 | Cystatin-8 | CST8 | M2 |
| Q9UHX1 | SL10575_31 | Poly(U)-binding-splicing factor PUF60 | PUF60 | M2 |
| Q6UW10 | SL10580_14 | Surfactant-associated protein 2 | SFTA2 | M2 |
| O43181 | SL10584_7 | NADH dehydrogenase [ubiquinone] iron-sulfur protein 4, mitochondrial | NDUS4 | M2 |
| P15941 | SL10623_19 | Mucin-1 | MUC1 | M2 |
| Q9UJJ9 | SL10666_7 | N-acetylglucosamine-1-phosphotransferase subunit gamma | GNPTG | M2 |
| Q9Y320 | SL10675_223 | Thioredoxin-related transmembrane protein 2 | TMX2 | M2 |
| O95168 | SL10677_9 | NADH dehydrogenase [ubiquinone] 1 beta subcomplex subunit 4 | NDUB4 | M2 |
| Q07954 | SL10699_52 | Prolow-density lipoprotein receptor-related protein 1 | LRP1 | M2 |
| Q5DX21 | SL10700_10 | Immunoglobulin superfamily member 11 | IGS11 | M2 |
| O43555 | SL10708_3 | Progonadoliberin-2 | GON2 | M2 |
| P0DMV8 | SL10721_76 | Heat shock 70 kDa protein 1A | HSP 70 | M2 |
| P30740 | SL10737_96 | Leukocyte elastase inhibitor | Serpin B1 | M2 |
| P0DMV8 | SL10749_18 | Heat shock 70 kDa protein 1A | HSP 70 | M2 |
| P48058 | SL10760_107 | Glutamate receptor 4 | GRIA4 | M2 |
| Q15363 | SL10761_5 | Transmembrane emp24 domain-containing protein 2 | TMED2 | M2 |
| Q8N6G5 | SL10772_21 | Chondroitin sulfate N-acetylgalactosaminyltransferase 2 | CGAT2 | M2 |
| Q496F6 | SL10798_4 | CMRF35-like molecule 2 | CLM2 | M2 |
| P0DMV8 | SL10803_22 | Heat shock 70 kDa protein 1A | HSP 70 | M2 |
| Q9UKJ1 | SL10816_150 | Paired immunoglobulin-like type 2 receptor alpha isoform FDF03-M14 | PILRA isoform FDF03-M14 | M2 |
| Q6UXN7 | SL10817_26 | TOMM20-like protein 1 | TO20L | M2 |
| Q9P109 | SL10842_7 | Beta-1,3-galactosyl-O-glycosyl-glycoprotein beta-1,6-N-acetylglucosaminyltransferase 4 | GCNT4 | M2 |
| Q6UWM7 | SL10890_135 | Lactase-like protein | LCTL | M2 |
| Q99650 | SL10892_8 | Oncostatin-M-specific receptor subunit beta | OSMR | M2 |
| Q86T26 | SL10895_28 | Transmembrane protease serine 11B | TM11B | M2 |
| Q53RT3 | SL10902_53 | Retroviral-like aspartic protease 1 | APRV1 | M2 |
| O75871 | SL10910_6 | Carcinoembryonic antigen-related cell adhesion molecule 4 | CEAM4 | M2 |
| Q9BXU9 | SL10933_107 | Calcium-binding protein 8 | CABP8 | M2 |
| P19256 | SL10938_13 | Lymphocyte function-associated antigen 3 | sLFA-3 | M2 |
| P05387 | SL10949_59 | 60S acidic ribosomal protein P2 | RLA2 | M2 |
| Q8IUN9 | SL10955_4 | C-type lectin domain family 10 member A | CLC10 | M2 |
| Q5S007 | SL10990_21 | Leucine-rich repeat serine/threonine-protein kinase 2 | LRRK2 | M2 |
| Q05315 | SL11094_104 | Galectin-10 | LPPL | M2 |
| Q8TB22 | SL11117_2 | Spermatogenesis-associated protein 20 | SPT20 | M2 |
| P32927 | SL11137_43 | Cytokine receptor common subunit beta | IL3RB | M2 |
| O95841 | SL11142_11 | Angiopoietin-related protein 1 | ANGL1 | M2 |
| Q6ZVL6 | SL11145_72 | UPF0606 protein KIAA1549L | K154L | M2 |
| Q9UKR3 | SL11152_46 | Kallikrein-13 | kallikrein 13 | M2 |
| P0DMV8 | SL11157_35 | Heat shock 70 kDa protein 1A | HSP 70 | M2 |
| Q13613 | SL11167_6 | Myotubularin-related protein 1 | MTMR1 | M2 |
| P21333 | SL11171_25 | Filamin-A | filamin A | M2 |
| Q92922 | SL11180_17 | SWI/SNF complex subunit SMARCC1 | SMRC1 | M2 |
| P30793 | SL11185_145 | GTP cyclohydrolase 1 | GCH1 | M2 |
| Q96BA8 | SL11198_37 | Cyclic AMP-responsive element-binding protein 3-like protein 1 | CR3L1 | M2 |
| Q99593 | SL11202_70 | T-box transcription factor TBX5 | TBX5 | M2 |
| P78310 | SL11204_80 | Coxsackievirus and adenovirus receptor | CXAR | M2 |
| P21757 | SL11207_3 | Macrophage scavenger receptor types I and II | Macrophage scavenger receptor | M2 |
| Q9UBS3 | SL11214_40 | DnaJ homolog subfamily B member 9 | DNJB9 | M2 |
| Q7Z5B4 | SL11228_37 | Protein RIC-3 | RIC3 | M2 |
| Q8N159 | SL11247_20 | N-acetylglutamate synthase, mitochondrial | NAGS | M2 |
| O95428 | SL11254_13 | Papilin | PPN | M2 |
| P00352 | SL11265_8 | Retinal dehydrogenase 1 | Retinal dehydrogenase 1 | M2 |
| Q8WYQ3 | SL11270_17 | Coiled-coil-helix-coiled-coil-helix domain-containing protein 10, mitochondrial | CHC10 | M2 |
| Q14451 | SL11281_6 | Growth factor receptor-bound protein 7 | GRB7 | M2 |
| Q96JK2 | SL11283_13 | DDB1- and CUL4-associated factor 5 | DCAF5 | M2 |
| Q9BQE4 | SL11286_78 | Selenoprotein S | SELS | M2 |
| Q6AZY7 | SL11292_13 | Scavenger receptor class A member 3 | SCAR3 | M2 |
| Q6UXK5 | SL11293_14 | Leucine-rich repeat neuronal protein 1 | LRRN1 | M2 |
| Q04721 | SL11297_54 | Neurogenic locus notch homolog protein 2 | NOTC2 | M2 |
| Q99523 | SL11300_32 | Sortilin | SORT | M2 |
| Q92752 | SL11302_237 | Tenascin-R | TENR | M2 |
| Q9Y3Z3 | SL11303_7 | Deoxynucleoside triphosphate triphosphohydrolase SAMHD1 | SAMH1 | M2 |
| P54278 | SL11312_40 | Mismatch repair endonuclease PMS2 | PMS2 | M2 |
| A1KZ92 | SL11324_3 | Peroxidasin-like protein | PXDNL | M2 |
| P78543 | SL11325_8 | Protein BTG2 | BTG2 | M2 |
| P49760 | SL11327_56 | Dual specificity protein kinase CLK2 | CLK2 | M2 |
| Q9BQ65 | SL11328_9 | U6 snRNA phosphodiesterase | USB1 | M2 |
| O75022 | SL11334_7 | Leukocyte immunoglobulin-like receptor subfamily B member 3 | LIRB3 | M2 |
| P37837 | SL11347_9 | Transaldolase | Transaldolase | M2 |
| O15460 | SL11348_132 | Prolyl 4-hydroxylase subunit alpha-2 | P4HA2 | M2 |
| Q96P66 | SL11371_1 | Probable G-protein coupled receptor 101 | GP101 | M2 |
| P17022 | SL11372_2 | Zinc finger protein 18 | ZNF18 | M2 |
| Q9BX46 | SL11380_84 | RNA-binding protein 24 | RBM24 | M2 |
| P08729 | SL11383_41 | Keratin, type II cytoskeletal 7 | Keratin 7 | M2 |
| Q9UKA2 | SL11416_23 | F-box/LRR-repeat protein 4 | FBXL4 | M2 |
| P16930 | SL11424_4 | Fumarylacetoacetase | FAAA | M2 |
| Q96T51 | SL11425_31 | RUN and FYVE domain-containing protein 1 | RUFY1 | M2 |
| P07910 | SL11429_80 | Heterogeneous nuclear ribonucleoproteins C1/C2 | hnRNP C1/C2 | M2 |
| Q86Y01 | SL11430_49 | E3 ubiquitin-protein ligase DTX1 | DTX1 | M2 |
| Q8IUC4 | SL11439_88 | Rhophilin-2 | RHPN2 | M2 |
| Q96LZ2 | SL11456_2 | Melanoma-associated antigen B10 | MAGBA | M2 |
| Q14376 | SL11457_53 | UDP-glucose 4-epimerase | GALE | M2 |
| P55199 | SL11459_81 | RNA polymerase II elongation factor ELL | ELL | M2 |
| Q01201 | SL11464_9 | Transcription factor RelB | RELB | M2 |
| Q8IZ08 | SL11465_4 | Probable G-protein coupled receptor 135 | GP135 | M2 |
| O14791 | SL11510_31 | Apolipoprotein L1 | Apo L1 | M2 |
| P28907 | SL11513_92 | ADP-ribosyl cyclase/cyclic ADP-ribose hydrolase 1 | CD38 | M2 |
| P07148 | SL11516_7 | Fatty acid-binding protein, liver | FABPL | M2 |
| P56282 | SL11562_9 | DNA polymerase epsilon subunit 2 | DPOE2 | M2 |
| Q9UHQ4 | SL11570_94 | B-cell receptor-associated protein 29 | BAP29 | M2 |
| Q8IWI9 | SL11587_5 | MAX gene-associated protein | MGAP | M2 |
| Q07325 | SL11593_21 | C-X-C motif chemokine 9 | MIG | M2 |
| P51815 | SL11596_47 | Zinc finger protein 75D | ZN75D | M2 |
| Q14562 | SL11601_26 | ATP-dependent RNA helicase DHX8 | DHX8 | M2 |
| P20701 | SL11617_1 | Integrin alpha-L | LFA-1 alpha-L chain | M2 |
| Q8IZ57 | SL11654_77 | Neurensin-1 | NRSN1 | M2 |
| O14512 | SL11657_86 | Suppressor of cytokine signaling 7 | SOCS7 | M2 |
| P57771 | SL11666_72 | Regulator of G-protein signaling 8 | RGS8 | M2 |
| Q63HR2 | SL11667_29 | Tensin-2 | TENC1 | M2 |
| P55201 | SL11671_19 | Peregrin | BRPF1 | M2 |
| Q96L93 | SL11672_17 | Kinesin-like protein KIF16B | KI16B | M2 |
| Q9UKL4 | SL11678_105 | Gap junction delta-2 protein | CXD2 | M2 |
| Q9H3M7 | SL11682_7 | Thioredoxin-interacting protein | TXNIP | M2 |
| Q9UJX3 | SL11690_47 | Anaphase-promoting complex subunit 7 | APC7 | M2 |
| P29373 | SL11696_7 | Cellular retinoic acid-binding protein 2 | RABP2 | M2 |
| A6NDA9 | SL11716_28 | Leucine-rich repeat, immunoglobulin-like domain and transmembrane domain-containing protein 2 | LRIT2 | M2 |
| Q68E01 | SL11934_9 | Integrator complex subunit 3 | INT3 | M2 |
| Q07960 | SL11955_1 | Rho GTPase-activating protein 1 | RHG01 | M2 |
| P29762 | SL11967_23 | Cellular retinoic acid-binding protein 1 | RABP1 | M2 |
| Q9HD43 | SL11988_24 | Receptor-type tyrosine-protein phosphatase H | PTPRH | M2 |
| Q07157 | SL12001_7 | Tight junction protein ZO-1 | ZO1 | M2 |
| P17661 | SL12030_82 | Desmin | Desmin | M2 |
| P34931 | SL12041_33 | Heat shock 70 kDa protein 1-like | HS71L | M2 |
| Q9BSQ5 | SL12347_29 | Cerebral cavernous malformations 2 protein | CCM2 | M2 |
| P15036 | SL12350_86 | Protein C-ets-2 | ETS2 | M2 |
| P07320 | SL12366_16 | Gamma-crystallin D | CRGD | M2 |
| Q9H9Q2 | SL12384_92 | COP9 signalosome complex subunit 7b | CSN7B | M2 |
| P20807 | SL12385_4 | Calpain-3 | CAN3 | M2 |
| P50479 | SL12387_7 | PDZ and LIM domain protein 4 | PDLI4 | M2 |
| Q9Y5N6 | SL12389_4 | Origin recognition complex subunit 6 | ORC6 | M2 |
| Q9H5P4 | SL12391_27 | PDZ domain-containing protein 7 | PDZD7 | M2 |
| Q9NPD8 | SL12400_25 | Ubiquitin-conjugating enzyme E2 T | UBE2T | M2 |
| Q96FJ0 | SL12401_3 | AMSH-like protease | STALP | M2 |
| Q96DA2 | SL12403_30 | Ras-related protein Rab-39B | RB39B | M2 |
| Q9Y3C4 | SL12417_46 | EKC/KEOPS complex subunit TPRKB | TPRKB | M2 |
| Q8N335 | SL12420_10 | Glycerol-3-phosphate dehydrogenase 1-like protein | GPD1L | M2 |
| Q9NQ94 | SL12423_38 | APOBEC1 complementation factor | A1CF | M2 |
| P30305 | SL12427_8 | M-phase inducer phosphatase 2 | MPIP2 | M2 |
| Q9BRX2 | SL12431_13 | Protein pelota homolog | PELO | M2 |
| P78417 | SL12436_84 | Glutathione S-transferase omega-1 | GST omega-1 | M2 |
| Q6PHR2 | SL12437_18 | Serine/threonine-protein kinase ULK3 | ULK3 | M2 |
| Q00978 | SL12439_67 | Interferon regulatory factor 9 | ISGF3 | M2 |
| O00212 | SL12442_4 | Rho-related GTP-binding protein RhoD | RHOD | M2 |
| Q96NW4 | SL12445_50 | Ankyrin repeat domain-containing protein 27 | ANR27 | M2 |
| O14867 | SL12451_62 | Transcription regulator protein BACH1 | BACH1 | M2 |
| Q86Y97 | SL12452_32 | Histone-lysine N-methyltransferase SUV420H2 | SV422 | M2 |
| Q9NPC7 | SL12455_48 | Myoneurin | MYNN | M2 |
| Q9P2N7 | SL12463_7 | Kelch-like protein 13 | KLH13 | M2 |
| Q9NXV2 | SL12473_48 | BTB/POZ domain-containing protein KCTD5 | KCTD5 | M2 |
| Q9NQ88 | SL12476_50 | Fructose-2,6-bisphosphatase TIGAR | TIGAR | M2 |
| Q96G74 | SL12480_9 | OTU domain-containing protein 5 | OTUD5 | M2 |
| P48739 | SL12484_67 | Phosphatidylinositol transfer protein beta isoform | PIPNB | M2 |
| Q9UHA4 | SL12490_92 | Ragulator complex protein LAMTOR3 | LTOR3 | M2 |
| O75347 | SL12501_10 | Tubulin-specific chaperone A | TBCA | M2 |
| Q96JW4 | SL12511_83 | Solute carrier family 41 member 2 | S41A2 | M2 |
| Q9UBP6 | SL12514_16 | tRNA (guanine-N(7)-)-methyltransferase | METTL1 | M2 |
| Q9BZX2 | SL12515_45 | Uridine-cytidine kinase 2 | UCK2 | M2 |
| Q15561 | SL12516_13 | Transcriptional enhancer factor TEF-3 | TEAD4 | M2 |
| B6SEH8 | SL12531_5 | Endogenous retrovirus group V member 1 Env polyprotein | ERVV1 | M2 |
| Q99418 | SL12533_135 | Cytohesin-2 | CYH2 | M2 |
| Q13137 | SL12534_10 | Calcium-binding and coiled-coil domain-containing protein 2 | CACO2 | M2 |
| P39748 | SL12577_100 | Flap endonuclease 1 | FLAP endonuclease-1 | M2 |
| P28074 | SL12580_7 | Proteasome subunit beta type-5 | PSB5 | M2 |
| O14732 | SL12581_39 | Inositol monophosphatase 2 | IMPA2 | M2 |
| P07992 | SL12585_39 | DNA excision repair protein ERCC-1 | ERCC1 | M2 |
| Q96GD3 | SL12604_16 | Polycomb protein SCMH1 | SCMH1 | M2 |
| Q9UHY1 | SL12616_45 | Nuclear receptor-binding protein | NRBP | M2 |
| Q04828 | SL12618_50 | Aldo-keto reductase family 1 member C1 | Aldo-keto reductase 1C1 | M2 |
| P53365 | SL12630_8 | Arfaptin-2 | ARFP2 | M2 |
| Q15119 | SL12651_21 | [Pyruvate dehydrogenase (acetyl-transferring)] kinase isozyme 2, mitochondrial | PDK2 | M2 |
| Q13630 | SL12657_2 | GDP-L-fucose synthase | FCL | M2 |
| O75351 | SL12668_7 | Vacuolar protein sorting-associated protein 4B | VPS4B | M2 |
| Q76N89 | SL12669_30 | E3 ubiquitin-protein ligase HECW1 | HECW1 | M2 |
| Q9BPU6 | SL12683_156 | Dihydropyrimidinase-related protein 5 | DPYL5 | M2 |
| Q9Y6U3 | SL12684_5 | Adseverin | ADSV | M2 |
| P49795 | SL12713_365 | Regulator of G-protein signaling 19 | RGS19 | M2 |
| Q9Y473 | SL12716_3 | Zinc finger protein 175 | ZN175 | M2 |
| Q86VP1 | SL12721_4 | Tax1-binding protein 1 | TAXB1 | M2 |
| Q9P2B2 | SL12727_7 | Prostaglandin F2 receptor negative regulator | FPRP | M2 |
| A6NDV4 | SL12742_160 | Transmembrane protein 8B | TMM8B | M2 |
| Q9UKY1 | SL12751_26 | Zinc fingers and homeoboxes protein 1 | ZHX1 | M2 |
| Q9Y534 | SL12754_14 | Cold shock domain-containing protein C2 | CSDC2 | M2 |
| O43424 | SL12758_47 | Glutamate receptor ionotropic, delta-2 | GRID2 | M2 |
| Q6NX45 | SL12760_34 | Zinc finger protein 774 | ZN774 | M2 |
| Q86XI6 | SL12768_3 | Protein phosphatase 1 regulatory subunit 3B | PPR3B | M2 |
| O15347 | SL12775_6 | High mobility group protein B3 | HMGB3 | M2 |
| P52741 | SL12787_47 | Zinc finger protein 134 | ZN134 | M2 |
| Q96J94 | SL12793_4 | Piwi-like protein 1 | PIWL1 | M2 |
| Q969W8 | SL12795_2 | Zinc finger protein 566 | ZN566 | M2 |
| Q2Y0W8 | SL12798_46 | Electroneutral sodium bicarbonate exchanger 1 | S4A8 | M2 |
| Q86UD4 | SL12803_9 | Zinc finger protein 329 | ZN329 | M2 |
| Q09FC8 | SL12811_55 | Zinc finger protein 415 | ZN415 | M2 |
| Q96LT9 | SL12814_17 | RNA-binding protein 40 | RBM40 | M2 |
| P41252 | SL12815_9 | Isoleucine--tRNA ligase, cytoplasmic | SYIC | M2 |
| O00213 | SL12822_34 | Amyloid beta A4 precursor protein-binding family B member 1 | APBB1 | M2 |
| P49796 | SL12827_37 | Regulator of G-protein signaling 3 | RGS3 | M2 |
| Q92974 | SL12848_9 | Rho guanine nucleotide exchange factor 2 | ARHG2 | M2 |
| Q96Q45 | SL12856_14 | Transmembrane protein 237 | TM237 | M2 |
| Q9UBL0 | SL12860_7 | cAMP-regulated phosphoprotein 21 | cAMP-regulated phosphoprotein 21 | M2 |
| Q14511 | SL12862_14 | Enhancer of filamentation 1 | CASL | M2 |
| Q0D2K0 | SL12864_9 | Magnesium transporter NIPA4 | NIPA4 | M2 |
| Q8WUN7 | SL12875_28 | Ubiquitin domain-containing protein 2 | UBTD2 | M2 |
| Q8IY33 | SL12891_1 | MICAL-like protein 2 | MILK2 | M2 |
| O60494 | SL12904_180 | cubilin | cubilin | M2 |
| Q04695 | SL12923_51 | Keratin, type I cytoskeletal 17 | Keratin 17 | M2 |
| Q8TC92 | SL12933_17 | Ecto-NOX disulfide-thiol exchanger 1 | ENOX1 | M2 |
| Q9UII4 | SL12934_1 | E3 ISG15--protein ligase HERC5 | HERC5 | M2 |
| O15160 | SL12939_1 | DNA-directed RNA polymerases I and III subunit RPAC1 | RPAC1 | M2 |
| P14927 | SL12957_62 | Cytochrome b-c1 complex subunit 7 | UCR6 | M2 |
| P35557 | SL12960_9 | Glucokinase | HXK4 | M2 |
| Q8IWT3 | SL12991_49 | Cullin-9 | CUL9 | M2 |
| Q6JVE6 | SL13007_66 | Epididymal-specific lipocalin-10 | LCN10 | M2 |
| O60260 | SL13013_41 | E3 ubiquitin-protein ligase parkin | PRKN2 | M2 |
| O14683 | SL13022_20 | Tumor protein p53-inducible protein 11 | P5I11 | M2 |
| Q8WU20 | SL13025_4 | Fibroblast growth factor receptor substrate 2 | FRS2 | M2 |
| Q14457 | SL13032_1 | Beclin-1 | BECN1 | M2 |
| Q6PL18 | SL13043_157 | ATPase family AAA domain-containing protein 2 | ATAD2 | M2 |
| A5PKW4 | SL13055_53 | PH and SEC7 domain-containing protein 1 | PSD1 | M2 |
| Q969G6 | SL13059_33 | Riboflavin kinase | RIFK | M2 |
| Q8WVN6 | SL13093_6 | Secreted and transmembrane protein 1 | SECTM1 | M2 |
| Q92843 | SL13097_11 | Bcl-2-like protein 2 | Apoptosis regulator Bcl-W | M2 |
| Q96BQ1 | SL13102_1 | Protein FAM3D | FAM3D | M2 |
| P98172 | SL13104_32 | Ephrin-B1 | EFNB1 | M2 |
| O95274 | SL13107_9 | Ly6/PLAUR domain-containing protein 3 | LYPD3 | M2 |
| Q9H4F8 | SL13118_5 | SPARC-related modular calcium-binding protein 1 | SMOC1 | M2 |
| P04004 | SL13125_45 | Vitronectin | Vitronectin | M2 |
| P19367 | SL13131_5 | Hexokinase-1 | HXK1 | M2 |
| O43761 | SL13375_48 | Synaptogyrin-3 | SNG3 | M2 |
| A6NC05 | SL13378_80 | Glutaredoxin-like protein C5orf63 | YD286 | M2 |
| Q969K3 | SL13386_248 | E3 ubiquitin-protein ligase RNF34 | RNF34 | M2 |
| Q16206 | SL13422_66 | Ecto-NOX disulfide-thiol exchanger 2 | ENOX2 | M2 |
| Q9H1F0 | SL13429_3 | WAP four-disulfide core domain protein 10A | WF10A | M2 |
| Q155Q3 | SL13441_30 | Dixin | DIXC1 | M2 |
| Q9H6D8 | SL13451_2 | Fibronectin type III domain-containing protein 4 | FNDC4 | M2 |
| Q96JN8 | SL13468_5 | Neuralized-like protein 4 | NEUL4 | M2 |
| Q03431 | SL13470_43 | Parathyroid hormone/parathyroid hormone-related peptide receptor | PTH1R | M2 |
| A8MVW0 | SL13479_8 | Protein FAM171A2 | F1712 | M2 |
| Q13563 | SL13486_9 | Polycystin-2 | PKD2 | M2 |
| Q8TDS4 | SL13495_48 | Hydroxycarboxylic acid receptor 2 | HCAR2 | M2 |
| Q01581 | SL13496_19 | Hydroxymethylglutaryl-CoA synthase, cytoplasmic | HMCS1 | M2 |
| Q9UGB7 | SL13498_1 | Inositol oxygenase | MIOX | M2 |
| Q8TBE7 | SL13501_10 | Solute carrier family 35 member G2 | S35G2 | M2 |
| Q86WA9 | SL13502_2 | Sodium-independent sulfate anion transporter | S2611 | M2 |
| Q15628 | SL13507_51 | Tumor necrosis factor receptor type 1-associated DEATH domain protein | TRADD | M2 |
| Q8TAC9 | SL13509_5 | Secretory carrier-associated membrane protein 5 | SCAM5 | M2 |
| O43390 | SL13512_28 | Heterogeneous nuclear ribonucleoprotein R | HNRPR | M2 |
| Q15286 | SL13514_121 | Ras-related protein Rab-35 | Rab-1C | M2 |
| Q96P16 | SL13515_8 | Regulation of nuclear pre-mRNA domain-containing protein 1A | RPR1A | M2 |
| Q5J8M3 | SL13516_46 | ER membrane protein complex subunit 4 | TMM85 | M2 |
| Q3KNS1 | SL13517_3 | Patched domain-containing protein 3 | PTHD3 | M2 |
| Q86VW2 | SL13519_112 | Rho guanine nucleotide exchange factor 25 | ARHGP | M2 |
| Q8TDN1 | SL13525_17 | Potassium voltage-gated channel subfamily G member 4 | KCNG4 | M2 |
| P05455 | SL13526_5 | Lupus La protein | LA | M2 |
| Q9UKP6 | SL13530_5 | Urotensin-2 receptor | UR2R | M2 |
| Q8N9A8 | SL13532_25 | Nuclear envelope phosphatase-regulatory subunit 1 | NEPR1 | M2 |
| P54296 | SL13534_20 | Myomesin-2 | MYOM2 | M2 |
| Q92952 | SL13539_131 | Small conductance calcium-activated potassium channel protein 1 | KCNN1 | M2 |
| Q8NDV2 | SL13540_1 | G-protein coupled receptor 26 | GPR26 | M2 |
| Q5VZY2 | SL13548_53 | Phosphatidate phosphatase PPAPDC1A | PPC1A | M2 |
| Q9UKF5 | SL13549_15 | Disintegrin and metalloproteinase domain-containing protein 29 | ADA29 | M2 |
| Q9BWE0 | SL13554_78 | Replication initiator 1 | REPI1 | M2 |
| Q9NQS3 | SL13557_3 | Nectin-3 | Nectin 3 | M2 |
| Q92730 | SL13574_50 | Rho-related GTP-binding protein Rho6 | RND1 | M2 |
| P26368 | SL13577_25 | Splicing factor U2AF 65 kDa subunit | U2AF2 | M2 |
| P49642 | SL13591_31 | DNA primase small subunit | PRI1 | M2 |
| O00194 | SL13596_3 | Ras-related protein Rab-27B | RB27B | M2 |
| Q7Z6M1 | SL13599_15 | Rab9 effector protein with kelch motifs | RABEK | M2 |
| Q8TAC1 | SL13603_7 | Rieske domain-containing protein | RFESD | M2 |
| O76050 | SL13604_27 | E3 ubiquitin-protein ligase NEURL1 | NEUL1 | M2 |
| Q9UJ72 | SL13605_16 | Annexin A10 | ANX10 | M2 |
| P43363 | SL13610_9 | Melanoma-associated antigen 10 | MAGE-10 | M2 |
| Q9Y6K8 | SL13613_23 | Adenylate kinase isoenzyme 5 | Adenylate kinase isoenzyme 5 | M2 |
| Q9Y6D9 | SL13618_15 | Mitotic spindle assembly checkpoint protein MAD1 | MD1L1 | M2 |
| O43681 | SL13620_10 | ATPase ASNA1 | ASNA | M2 |
| O14686 | SL13623_4 | Histone-lysine N-methyltransferase 2D | MLL2 | M2 |
| P05455 | SL13625_19 | Lupus La protein | LA | M2 |
| O00625 | SL13634_209 | Pirin | PIR | M2 |
| Q92620 | SL13645_14 | Pre-mRNA-splicing factor ATP-dependent RNA helicase PRP16 | PRP16 | M2 |
| Q9NZI2 | SL13650_11 | Kv channel-interacting protein 1 | KCIP1 | M2 |
| Q9BQ69 | SL13653_335 | O-acetyl-ADP-ribose deacetylase MACROD1 | MACD1 | M2 |
| Q9NS85 | SL13666_222 | Carbonic anhydrase-related protein 10 | Carbonic Anhydrase X | M2 |
| P22607 | SL13669_6 | Fibroblast growth factor receptor 3 | FGFR-3 | M2 |
| P17066 | SL13672_3 | Heat shock 70 kDa protein 6 | HSP76 | M2 |
| Q8WWF8 | SL13688_2 | Calcyphosin-like protein | CAPSL | M2 |
| P21695 | SL13697_51 | Glycerol-3-phosphate dehydrogenase [NAD(+)], cytoplasmic | GPDA | M2 |
| Q8IZA0 | SL13698_28 | Dyslexia-associated protein KIAA0319-like protein | K319L | M2 |
| P54868 | SL13704_5 | Hydroxymethylglutaryl-CoA synthase, mitochondrial | HMCS2 | M2 |
| Q15485 | SL13717_15 | Ficolin-2 | FCN2 | M2 |
| P24158 | SL13720_95 | Myeloblastin | Proteinase-3 | M2 |
| P33681 | SL13726_4 | T-lymphocyte activation antigen CD80 | B7 | M2 |
| O43791 | SL13727_44 | Speckle-type POZ protein | SPOP | M2 |
| P30040 | SL13728_19 | Endoplasmic reticulum resident protein 29 | ERP29 | M2 |
| Q92765 | SL13740_51 | Secreted frizzled-related protein 3 | sFRP-3 | M2 |
| Q13563 | SL13745_10 | Polycystin-2 | PKD2 | M2 |
| Q9H9A7 | SL13926_1 | RecQ-mediated genome instability protein 1 | RMI1 | M2 |
| Q14155 | SL13932_45 | Rho guanine nucleotide exchange factor 7 | COOL-1 | M2 |
| Q96BD6 | SL13942_140 | SPRY domain-containing SOCS box protein 1 | SPSB1 | M2 |
| O75899 | SL13948_50 | Gamma-aminobutyric acid type B receptor subunit 2 | GABR2 | M2 |
| Q96P47 | SL13960_15 | Arf-GAP with GTPase, ANK repeat and PH domain-containing protein 3 | AGAP3 | M2 |
| Q5VTT5 | SL13966_30 | Myomesin-3 | MYOM3 | M2 |
| Q99728 | SL13977_28 | BRCA1-associated RING domain protein 1 | BARD1 | M2 |
| Q9UHL0 | SL13984_23 | ATP-dependent RNA helicase DDX25 | DDX25 | M2 |
| P11498 | SL13990_1 | Pyruvate carboxylase, mitochondrial | pyruvate carboxylase | M2 |
| Q9BZ29 | SL14002_18 | Dedicator of cytokinesis protein 9 | DOCK9 | M2 |
| Q9P2D1 | SL14005_2 | Chromodomain-helicase-DNA-binding protein 7 | CHD7 | M2 |
| Q14749 | SL14006_36 | Glycine N-methyltransferase | GNMT | M2 |
| Q92817 | SL14019_73 | Envoplakin | EVPL | M2 |
| Q92838 | SL14024_196 | Ectodysplasin-A, secreted form | EDA | M2 |
| O00443 | SL14028_22 | Phosphatidylinositol 4-phosphate 3-kinase C2 domain-containing subunit alpha | P3C2A | M2 |
| P09758 | SL14034_22 | Tumor-associated calcium signal transducer 2 | GA733-1 protein | M2 |
| Q15075 | SL14043_12 | Early endosome antigen 1 | EEA1 | M2 |
| Q15596 | SL14045_12 | Nuclear receptor coactivator 2 | NCOA2 | M2 |
| Q99958 | SL14051_54 | Forkhead box protein C2 | FOXC2 | M2 |
| Q13261 | SL14054_17 | Interleukin-15 receptor subunit alpha | IL-15 Ra | M2 |
| P01215 | SL14056_4 | Glycoprotein hormones alpha chain | Glycoprotein hormones a-chain | M2 |
| O14788 | SL14061_48 | Tumor necrosis factor ligand superfamily member 11 | sRANKL | M2 |
| P13725 | SL14063_17 | Oncostatin-M | OSM | M2 |
| P98077 | SL14074_2 | SHC-transforming protein 2 | SHC2 | M2 |
| P49771 | SL14093_10 | Fms-related tyrosine kinase 3 ligand | Flt3 ligand | M2 |
| P26718 | SL14095_1 | NKG2-D type II integral membrane protein | NKG2D | M2 |
| Q969Z4 | SL14112_40 | Tumor necrosis factor receptor superfamily member 19L | RELT | M2 |
| P26447 | SL14116_129 | Protein S100-A4 | S100A4 | M2 |
| Q68DV7 | SL14120_2 | E3 ubiquitin-protein ligase RNF43 | RNF43 | M2 |
| Q9UBN6 | SL14121_24 | Tumor necrosis factor receptor superfamily member 10D | TRAIL R4 | M2 |
| Q9ULT6 | SL14122_132 | E3 ubiquitin-protein ligase ZNRF3 | ZNRF3 | M2 |
| P01566 | SL14128_121 | Interferon alpha-10 | IFN10 | M2 |
| Q9NPY3 | SL14136_234 | Complement component C1q receptor | C1QR1 | M2 |
| Q8WWG1 | SL14139_16 | Neuregulin-4 | NRG4 | M2 |
| Q9NZH7 | SL14149_9 | Interleukin-36 beta | IL-1F8 | M2 |
| Q9UHA7 | SL14150_7 | Interleukin-36 alpha | IL-1F6 | M2 |
| P05161 | SL14151_4 | Ubiquitin-like protein ISG15 | UCRP | M2 |
| Q8WZ73 | SL14186_13 | E3 ubiquitin-protein ligase rififylin | RFFL | M2 |
| O75626 | SL14197_2 | PR domain zinc finger protein 1 | PRDM1 | M2 |
| O00213 | SL14206_28 | Amyloid beta A4 precursor protein-binding family B member 1 | APBB1 | M2 |
| Q96R05 | SL14208_3 | Retinoid-binding protein 7 | RET7 | M2 |
| O95396 | SL14229_5 | Adenylyltransferase and sulfurtransferase MOCS3 | MOCS3 | M2 |
| P0DMV8 | SL14237_1 | Heat shock 70 kDa protein 1A | HSP 70 | M2 |
| Q9UNH6 | SL14245_195 | Sorting nexin-7 | SNX7 | M2 |
| Q9UJV3 | SL14249_68 | Probable E3 ubiquitin-protein ligase MID2 | TRIM1 | M2 |
| Q9BYN0 | SL14268_4 | Sulfiredoxin-1 | SRXN1 | M2 |
| P31943 | SL14309_8 | Heterogeneous nuclear ribonucleoprotein H | HNRH1 | M2 |
| Q13356 | SL14314_6 | Peptidyl-prolyl cis-trans isomerase-like 2 | PPIL2 | M2 |
| P35243 | SL14334_3 | Recoverin | RECO | M2 |
| O14793 | SL14583_49 | Growth/differentiation factor 8 | Myostatin | M2 |
| O95390 | SL14587_16 | Growth/differentiation factor 11 | GDF-11 | M2 |
| Q9H3T2 | SL14597_5 | Semaphorin-6C, cytoplasmic | SEM6C | M2 |
| Q9NY15 | SL14599_18 | Stabilin-1 | STAB1 | M2 |
| P49711 | SL14624_51 | Transcriptional repressor CTCF | CTCF | M2 |
| O15217 | SL14645_253 | Glutathione S-transferase A4 | GSTA4 | M2 |
| O76064 | SL14663_44 | E3 ubiquitin-protein ligase RNF8 | RNF8 | M2 |
| P17655 | SL14684_17 | Calpain-2 catalytic subunit | CAN2 | M2 |
| P31751 | SL14685_17 | RAC-beta serine/threonine-protein kinase | PKB beta | M2 |
| P55735 | SL14689_3 | Protein SEC13 homolog | SEC13 | M2 |
| Q8N554 | SL14692_3 | Zinc finger protein 276 | ZN276 | M2 |
| O75462 | SL14747_9 | Cytokine receptor-like factor 1 | CRLF1 | M2 |
| P33763 | SL15303_63 | Protein S100-A5 | S100A5 | M2 |
| P01591 | SL15306_20 | Immunoglobulin J chain | IgJ | M2 |
| Q6Q788 | SL15363_32 | Apolipoprotein A-V | Apo A-V | M2 |
| P09488 | SL15395_15 | Glutathione S-transferase Mu 1 | GST M1-1 | M2 |
| P41134 | SL15402_2 | DNA-binding protein inhibitor ID-1 | ID-1 | M2 |
| P05014 | SL15405_23 | Interferon alpha-4 | IFNA4 | M2 |
| P21589 | SL15452_5 | 5'-Nucleotidase | 5'-Nucleotidase | M2 |
| P49913 | SL15481_45 | Antibacterial protein LL-37 | LL-37 | M2 |
| P21757 | SL15533_97 | Macrophage scavenger receptor types I and II | Macrophage scavenger receptor | M2 |
| Q9P0G3 | SL15544_25 | Kallikrein-14 | kallikrein 14 | M2 |
| P63098 | SL15545_13 | Calcineurin subunit B type 1 | Calcineurin B a | M2 |
| O75022 | SL15615_8 | Leukocyte immunoglobulin-like receptor subfamily B member 3 | LIRB3 | M2 |
| Q14982 | SL15622_13 | Opioid-binding protein/cell adhesion molecule | OBCAM | M2 |
| Q15116 | SL15623_1 | Programmed cell death protein 1 | PD-1 | M2 |
| P02753 | SL15633_6 | Retinol-binding protein 4 | RBP | M2 |
| P43251 | SL15644_1 | Biotinidase | Biotinidase | M2 |
| P17676 | SL15675_3 | CCAAT/enhancer-binding protein beta | CEBPB | M2 |
| Q9BXR6 | SL16055_3 | Complement factor H-related protein 5 | complement factor H-related 5 | M2 |
| Q9Y5W5 | SL16070_7 | Wnt inhibitory factor 1 | WIF-1 | M2 |
| Q96DR5 | SL16302_11 | BPI fold-containing family A member 2 | SPLC2 | M2 |
| P55285 | SL16312_45 | Cadherin-6 | Cadherin-6 | M2 |
| P17948 | SL16315_105 | Vascular endothelial growth factor receptor 1 | VEGF sR1 | M2 |
| P0C862 | SL16605_2 | Complement C1q and tumor necrosis factor-related protein 9A | C1T9A | M2 |
| P0DMV8 | SL16780_6 | Heat shock 70 kDa protein 1A | HSP 70 | M2 |
| P35080 | SL16923_20 | Profilin-2 | Profilin II | M2 |
| P55075 | SL17166_4 | Fibroblast growth factor 8 isoform F | FGF-8F | M2 |
| P09923 | SL17441_4 | Intestinal-type alkaline phosphatase | Alkaline phosphatase, intestine | M2 |
| Q8NBJ4 | SL17456_53 | Golgi membrane protein 1 | GOLM1 | M2 |
| P10914 | SL17462_19 | Interferon regulatory factor 1 | IRF1 | M2 |
| P09105 | SL18198_51 | Hemoglobin subunit theta-1 | HBAT | M2 |
| P30085 | SL18243_9 | UMP-CMP kinase | Cytidylate kinase | M2 |
| P48960 | SL18881_7 | CD97 antigen | CD97 | M2 |
| O96017 | SL19328_51 | Serine/threonine-protein kinase Chk2 | Chk2 | M2 |
| Q9H422 | SL19329_31 | Homeodomain-interacting protein kinase 3 | HIPK3 | M2 |
| Q86UE6 | SL19360_22 | Leucine-rich repeat transmembrane neuronal protein 1 | LRRT1 | M2 |
| O15232 | SL19361_78 | Matrilin-3 | MATN3 | M2 |
| P12004 | SL19364_163 | Proliferating cell nuclear antigen | PCNA | M2 |
| O95897 | SL19377_14 | Noelin-2 | NOE2 | M2 |
| Q16623 | SL19553_14 | Syntaxin-1A | STX1a | M2 |
| P00533 | SL19567_1 | Epidermal growth factor receptor variant III | EGFRvIII | M2 |
| P55075 | SL19570_12 | Fibroblast growth factor 8 | FGF-8 | M2 |
| P24593 | SL19581_15 | Insulin-like growth factor-binding protein 5 | IGFBP-5 | M2 |
| P31371 | SL19584_33 | Fibroblast growth factor 9 | FGF9 | M2 |
| Q92994 | SL19596_18 | Transcription factor IIIB 90 kDa subunit | BRF-1 | M2 |
| P08865 | SL19623_26 | 40S ribosomal protein SA | 40S ribosomal protein SA | M2 |
| P06850 | SL19637_9 | Corticoliberin | CRH | M2 |
| P03951 | SL2190_55 | Coagulation Factor XI | Coagulation Factor XI | M2 |
| P00750 | SL2212_69 | Tissue-type plasminogen activator | tPA | M2 |
| P01031 | SL2381_52 | Complement C5 | C5 | M2 |
| P02649 | SL2418_55 | Apolipoprotein E | Apo E | M2 |
| P55075 | SL2443_10 | Fibroblast growth factor 8 isoform B | FGF-8B | M2 |
| Q9NZK7 | SL2447_7 | Group IIE secretory phospholipase A2 | GIIE | M2 |
| P02743 | SL2474_54 | Serum amyloid P-component | SAP | M2 |
| P10721 | SL2475_1 | Mast/stem cell growth factor receptor Kit | SCF sR | M2 |
| Q15768 | SL2514_65 | Ephrin-B3 | Ephrin-B3 | M2 |
| P09429 | SL2524_56 | High mobility group protein B1 | HMG-1 | M2 |
| P01236 | SL2585_2 | Prolactin | PRL | M2 |
| P28908 | SL2605_49 | Tumor necrosis factor receptor superfamily member 8 | CD30 | M2 |
| Q06418 | SL2611_72 | Tyrosine-protein kinase receptor TYRO3 | Dtk | M2 |
| P04626 | SL2616_23 | Receptor tyrosine-protein kinase erbB-2 | ERBB2 | M2 |
| Q15303 | SL2618_10 | Receptor tyrosine-protein kinase erbB-4 | ERBB4 | M2 |
| P09758 | SL2619_72 | Tumor-associated calcium signal transducer 2 | GA733-1 protein | M2 |
| Q08334 | SL2631_50 | Interleukin-10 receptor subunit beta | IL-10 Rb | M2 |
| P42701 | SL2632_5 | Interleukin-12 receptor subunit beta-1 | IL-12 Rb1 | M2 |
| Q6UX15 | SL2635_61 | Layilin | Layilin | M2 |
| P36941 | SL2636_10 | Tumor necrosis factor receptor superfamily member 3 | Lymphotoxin b R | M2 |
| Q04912 | SL2640_3 | Macrophage-stimulating protein receptor | MSP R | M2 |
| P22223 | SL2643_57 | Cadherin-3 | P-Cadherin | M2 |
| P32942 | SL2649_77 | Intercellular adhesion molecule 3 | sICAM-3 | M2 |
| P00533 | SL2677_1 | Epidermal growth factor receptor | ERBB1 | M2 |
| P01024 | SL2683_1 | Complement C3b, inactivated | iC3b | M2 |
| P13725 | SL2693_20 | Oncostatin-M | OSM | M2 |
| O60542 | SL2696_87 | Persephin | Persephin | M2 |
| O14836 | SL2704_74 | Tumor necrosis factor receptor superfamily member 13B | TACI | M2 |
| O15444 | SL2705_5 | C-C motif chemokine 25 | TECK | M2 |
| Q9UNG2 | SL2708_54 | Tumor necrosis factor ligand superfamily member 18 | TNFSF18 | M2 |
| Q99062 | SL2719_3 | Granulocyte colony-stimulating factor receptor | G-CSF-R | M2 |
| Q9NZH6 | SL2723_9 | Interleukin-37 | IL-1F7 | M2 |
| P48745 | SL2737_22 | Protein NOV homolog | NovH | M2 |
| P02647 | SL2750_3 | Apolipoprotein A-I | Apo A-I | M2 |
| P01024 | SL2754_50 | Complement C3 | C3 | M2 |
| O95990 | SL2760_2 | Protein FAM107A | DRR1 | M2 |
| P22362 | SL2770_51 | C-C motif chemokine 1 | I-309 | M2 |
| Q14005 | SL2774_10 | Interleukin-16 | IL-16 | M2 |
| P36896 | SL2806_49 | Activin receptor type-1B | Activin RIB | M2 |
| O75173 | SL2809_25 | A disintegrin and metalloproteinase with thrombospondin motifs 4 | ADAMTS-4 | M2 |
| Q92838 | SL2826_53 | Ectodysplasin-A, secreted form | EDA | M2 |
| P78423 | SL2827_23 | Fractalkine | Fractalkine/CX3CL-1 | M2 |
| O43278 | SL2828_82 | Kunitz-type protease inhibitor 1 | HAI-1 | M2 |
| Q9UBX7 | SL2831_29 | Kallikrein-11 | Kallikrein 11 | M2 |
| P23510 | SL2839_2 | Tumor necrosis factor ligand superfamily member 4 | OX40 Ligand | M2 |
| P55008 | SL2849_49 | Allograft inflammatory factor 1 | AIF1 | M2 |
| P01031 | SL2851_63 | C5a anaphylatoxin | C5a | M2 |
| O14757 | SL2853_68 | Serine/threonine-protein kinase Chk1 | CHK1 | M2 |
| Q9BY41 | SL2859_69 | Histone deacetylase 8 | HDAC8 | M2 |
| Q06609 | SL2871_73 | DNA repair protein RAD51 homolog 1 | RAD51 | M2 |
| P11387 | SL2876_74 | DNA topoisomerase 1 | Topoisomerase I | M2 |
| P10643 | SL2888_49 | Complement component C7 | C7 | M2 |
| Q16619 | SL2889_37 | Cardiotrophin-1 | Cardiotrophin-1 | M2 |
| Q5T4W7 | SL2939_10 | Artemin | Artemin | M2 |
| P08684 | SL2943_5 | Cytochrome P450 3A4 | Cytochrome P450 3A4 | M2 |
| P00746 | SL2946_52 | Complement factor D | Factor D | M2 |
| O15496 | SL2949_6 | Group 10 secretory phospholipase A2 | GX | M2 |
| P05019 | SL2952_75 | Insulin-like growth factor I | IGF-I | M2 |
| P12272 | SL2962_50 | Parathyroid hormone-related protein | PTHrP | M2 |
| Q9Y240 | SL2966_65 | Stem cell growth factor-beta | SCGF-beta | M2 |
| P19320 | SL2967_8 | Vascular cell adhesion protein 1 | VCAM-1 | M2 |
| Q02413 | SL2976_58 | Desmoglein-1 | Desmoglein-1 | M2 |
| Q9HB29 | SL2994_71 | Interleukin-1 receptor-like 2 | IL-1Rrp2 | M2 |
| P18031 | SL3005_5 | Tyrosine-protein phosphatase non-receptor type 1 | PTP-1B | M2 |
| Q9Y336 | SL3007_7 | Sialic acid-binding Ig-like lectin 9 | Siglec-9 | M2 |
| P08697 | SL3024_18 | Alpha-2-antiplasmin | a2-Antiplasmin | M2 |
| Q9H2X3 | SL3030_3 | C-type lectin domain family 4 member M | DC-SIGNR | M2 |
| P10147 | SL3040_59 | C-C motif chemokine 3 | MIP-1a | M2 |
| Q9Y275 | SL3059_50 | Tumor necrosis factor ligand superfamily member 13B | BAFF | M2 |
| P02748 | SL3060_43 | Complement component C9 | C9 | M2 |
| P35225 | SL3072_4 | Interleukin-13 | IL-13 | M2 |
| P49763 | SL3078_1 | Placenta growth factor | PlGF | M2 |
| Q9BZM6 | SL3081_70 | NKG2D ligand 1 | ULBP-1 | M2 |
| O14965 | SL3091_70 | Aurora kinase A | Aurora kinase A | M2 |
| Q9UNA0 | SL3168_8 | A disintegrin and metalloproteinase with thrombospondin motifs 5 | ADAMTS-5 | M2 |
| P35475 | SL3169_70 | Alpha-L-iduronidase | IDUA | M2 |
| P15848 | SL3172_28 | Arylsulfatase B | ARSB | M2 |
| P53634 | SL3178_5 | Dipeptidyl peptidase 1 | CATC | M2 |
| P49961 | SL3182_38 | Ectonucleoside triphosphate diphosphohydrolase 1 | CD39 | M2 |
| P08709 | SL3184_25 | Coagulation factor VII | Coagulation Factor VII | M2 |
| Q96KP4 | SL3192_3 | Cytosolic non-specific dipeptidase | Glutamate carboxypeptidase | M2 |
| P10915 | SL3196_6 | Hyaluronan and proteoglycan link protein 1 | HPLN1 | M2 |
| Q9UKR0 | SL3199_54 | Kallikrein-12 | kallikrein 12 | M2 |
| P09960 | SL3204_2 | Leukotriene A-4 hydrolase | LKHA4 | M2 |
| Q9Y5Y7 | SL3206_4 | Lymphatic vessel endothelial hyaluronic acid receptor 1 | LYVE1 | M2 |
| Q9NQ76 | SL3209_69 | Matrix extracellular phosphoglycoprotein | MEPE | M2 |
| P13686 | SL3232_28 | Tartrate-resistant acid phosphatase type 5 | TrATPase | M2 |
| P00736 | SL3285_23 | Complement C1r subcomponent | C1r | M2 |
| O94779 | SL3299_29 | Contactin-5 | Contactin-5 | M2 |
| Q15485 | SL3313_21 | Ficolin-2 | FCN2 | M2 |
| P05546 | SL3316_58 | Heparin cofactor 2 | Heparin cofactor II | M2 |
| O75594 | SL3329_14 | Peptidoglycan recognition protein 1 | PGRP-S | M2 |
| Q03154 | SL3343_1 | Aminoacylase-1 | Aminoacylase-1 | M2 |
| Q96GD4 | SL3346_72 | Aurora kinase B | AURKB | M2 |
| Q9UJ71 | SL3361_26 | C-type lectin domain family 4 member K | CLC4K | M2 |
| Q8NFM7 | SL3376_49 | Interleukin-17 receptor D | IL-17 RD | M2 |
| O75914 | SL3387_1 | Serine/threonine-protein kinase PAK 3 | PAK3 | M2 |
| P48736 | SL3391_10 | Phosphatidylinositol 4,5-bisphosphate 3-kinase catalytic subunit gamma isoform | PK3CG | M2 |
| P53350 | SL3394_81 | Serine/threonine-protein kinase PLK1 | PLK-1 | M2 |
| Q8WWQ8 | SL3399_31 | Stabilin-2 | STAB2 | M2 |
| Q9NRR2 | SL3404_51 | Tryptase gamma | TPSG1 | M2 |
| Q16548 | SL3413_50 | Bcl-2-related protein A1 | BFL1 | M2 |
| P21815 | SL3415_61 | Bone sialoprotein 2 | BSP | M2 |
| Q8IU85 | SL3418_12 | Calcium/calmodulin-dependent protein kinase type 1D | CAMK1D | M2 |
| P32971 | SL3421_54 | Tumor necrosis factor ligand superfamily member 8 | CD30 Ligand | M2 |
| P21709 | SL3431_54 | Ephrin type-A receptor 1 | EphA1 | M2 |
| Q13261 | SL3445_53 | Interleukin-15 receptor subunit alpha | IL-15 Ra | M2 |
| P10145 | SL3447_64 | Interleukin-8 | IL-8 | M2 |
| Q92876 | SL3450_4 | Kallikrein-6 | Kallikrein 6 | M2 |
| O75716 | SL3471_49 | Serine/threonine-protein kinase 16 | STK16 | M2 |
| O15392 | SL3472_40 | Baculoviral IAP repeat-containing protein 5 | Survivin | M2 |
| P01019 | SL3484_60 | Angiotensinogen | Angiotensinogen | M2 |
| P26441 | SL3489_9 | Ciliary neurotrophic factor | CNTF | M2 |
| O60258 | SL3494_71 | Fibroblast growth factor 17 | FGF-17 | M2 |
| P01563 | SL3497_13 | Interferon alpha-2 | IFN-aA | M2 |
| Q9UHF5 | SL3499_77 | Interleukin-17B | IL-17B | M2 |
| P10600 | SL3520_58 | Transforming growth factor beta-3 | TGF-b3 | M2 |
| P20711 | SL3538_26 | Aromatic-L-amino-acid decarboxylase | dopa decarboxylase | M2 |
| P02765 | SL3581_53 | Alpha-2-HS-glycoprotein | a2-HS-Glycoprotein | M2 |
| O95393 | SL3587_53 | Bone morphogenetic protein 10 | BMP10 | M2 |
| Q8N6P7 | SL3620_67 | Interleukin-22 receptor subunit alpha-1 | IL22RA1 | M2 |
| Q99538 | SL3622_33 | Legumain | LGMN | M2 |
| P30533 | SL3640_14 | alpha-2-macroglobulin receptor-associated protein | RAP | M2 |
| P01023 | SL3708_62 | Alpha-2-macroglobulin | a2-Macroglobulin | M2 |
| P00747 | SL3710_49 | Angiostatin | Angiostatin | M2 |
| P09683 | SL3728_52 | Secretin | Secretin | M2 |
| Q13443 | SL3795_6 | Disintegrin and metalloproteinase domain-containing protein 9 | ADAM 9 | M2 |
| P19022 | SL3797_1 | Cadherin-2 | Cadherin-2 | M2 |
| P12277 | SL3800_71 | Creatine kinase B-type | CK-BB | M2 |
| Q9GZV9 | SL3807_1 | Fibroblast growth factor 23 | FGF23 | M2 |
| P22607 | SL3809_1 | Fibroblast growth factor receptor 3 | FGFR-3 | M2 |
| Q8IW41 | SL3821_28 | MAP kinase-activated protein kinase 5 | MAPK5 | M2 |
| P43403 | SL3837_6 | Tyrosine-protein kinase ZAP-70 | ZAP70 | M2 |
| O95571 | SL3847_56 | Persulfide dioxygenase ETHE1, mitochondrial | ETHE1 | M2 |
| P07202 | SL3873_51 | Thyroid peroxidase | TMA | M2 |
| P63167 | SL3881_49 | Dynein light chain 1, cytoplasmic | DLC8 | M2 |
| Q13421 | SL3893_64 | Mesothelin | Mesothelin | M2 |
| P0DMV8 | SL4124_24 | Heat shock 70 kDa protein 1A | HSP 70 | M2 |
| P13671 | SL4127_75 | Complement component C6 | C6 | M2 |
| O00175 | SL4128_27 | C-C motif chemokine 24 | Eotaxin-2 | M2 |
| P10144 | SL4133_54 | Granzyme B | Granzyme B | M2 |
| P00747 | SL4150_75 | Plasmin | Plasmin | M2 |
| P00747 | SL4151_6 | Plasminogen | Plasminogen | M2 |
| Q9UIC8 | SL4237_70 | Leucine carboxyl methyltransferase 1 | LCMT1 | M2 |
| P04183 | SL4301_58 | Thymidine kinase, cytosolic | Thymidine kinase | M2 |
| Q8N3X6 | SL4304_18 | Ligand-dependent nuclear receptor corepressor-like protein | transcription factor MLR1, isoform CRA_b | M2 |
| O43320 | SL4393_3 | Fibroblast growth factor 16 | FGF-16 | M2 |
| P55075 | SL4394_71 | Fibroblast growth factor 8 isoform A | FGF-8A | M2 |
| O43184 | SL4420_7 | Disintegrin and metalloproteinase domain-containing protein 12 | ADAM12 | M2 |
| Q9GZX3 | SL4429_51 | Carbohydrate sulfotransferase 6 | CHST6 | M2 |
| O75355 | SL4436_1 | Ectonucleoside triphosphate diphosphohydrolase 3 | ENTP3 | M2 |
| O75356 | SL4437_56 | Ectonucleoside triphosphate diphosphohydrolase 5 | ENTP5 | M2 |
| Q96P31 | SL4440_15 | Fc receptor-like protein 3 | FCRL3 | M2 |
| Q16549 | SL4459_68 | Proprotein convertase subtilisin/kexin type 7 | PCSK7 | M2 |
| Q14515 | SL4467_49 | SPARC-like protein 1 | SPARCL1 | M2 |
| Q9NRA0 | SL4468_21 | Sphingosine kinase 2 | SPHK2 | M2 |
| P07951 | SL4472_5 | Tropomyosin beta chain | Tropomyosin 2 | M2 |
| P43403 | SL4476_22 | Tyrosine-protein kinase ZAP-70 | ZAP70 | M2 |
| P01024 | SL4480_59 | Complement C3b | C3b | M2 |
| P13591 | SL4498_62 | Neural cell adhesion molecule 1, 120 kDa isoform | NCAM-120 | M2 |
| Q9Y240 | SL4500_50 | Stem cell growth factor-alpha | SCGF-alpha | M2 |
| P45973 | SL4540_11 | Chromobox protein homolog 5 | CBX5 | M2 |
| P21217 | SL4548_4 | Galactoside 3(4)-L-fucosyltransferase | Fucosyltransferase 3 | M2 |
| P58417 | SL4562_1 | Neurexophilin-1 | NXPH1 | M2 |
| P01298 | SL4588_1 | Pancreatic hormone | PH | M2 |
| P05231 | SL4673_13 | Interleukin-6 | IL-6 | M2 |
| P01374 | SL4703_87 | Lymphotoxin-alpha | TNF-b | M2 |
| P05090 | SL4712_28 | Apolipoprotein D | Apo D | M2 |
| P08700 | SL4717_55 | Interleukin-3 | IL-3 | M2 |
| P43652 | SL4763_31 | Afamin | Afamin | M2 |
| Q92484 | SL4771_10 | Acid sphingomyelinase-like phosphodiesterase 3a | ASM3A | M2 |
| Q13873 | SL4862_63 | Bone morphogenetic protein receptor type-2 | BMP RII | M2 |
| P00740 | SL4876_32 | Coagulation factor IX | Coagulation Factor IX | M2 |
| P01308 | SL4883_56 | Insulin | Insulin | M2 |
| P01275 | SL4891_50 | Glucagon | Glucagon | M2 |
| P01024 | SL4900_8 | C3a anaphylatoxin | C3a | M2 |
| O00214 | SL4909_68 | Galectin-8 | Galectin-8 | M2 |
| P17174 | SL4912_17 | Aspartate aminotransferase, cytoplasmic | GOT1 | M2 |
| O95994 | SL4959_2 | Anterior gradient protein 2 homolog | AGR2 | M2 |
| P04083 | SL4960_72 | Annexin A1 | annexin I | M2 |
| Q07021 | SL4967_1 | Complement component 1 Q subcomponent-binding protein, mitochondrial | C1QBP | M2 |
| Q14574 | SL4981_6 | Desmocollin-3 | DSC3 | M2 |
| Q01469 | SL4985_11 | Fatty acid-binding protein, epidermal | FABPE | M2 |
| Q05397 | SL4986_59 | Focal adhesion kinase 1 | FAK1 | M2 |
| P24071 | SL4987_17 | Immunoglobulin alpha Fc receptor | FCAR | M2 |
| P02679 | SL4989_7 | Fibrinogen gamma chain | Fibrinogen g-chain dimer | M2 |
| Q16772 | SL4993_16 | Glutathione S-transferase A3 | GSTA3 | M2 |
| P04196 | SL4996_66 | Histidine-rich glycoprotein | HRG | M2 |
| P50281 | SL5002_76 | Matrix metalloproteinase-14 | MMP-14 | M2 |
| P04179 | SL5008_51 | Superoxide dismutase [Mn], mitochondrial | Mn SOD | M2 |
| P06400 | SL5024_67 | Retinoblastoma-associated protein | Rb | M2 |
| Q13813 | SL5031_10 | Spectrin alpha chain, non-erythrocytic 1 | SPTA2 | M2 |
| P09493 | SL5033_27 | Tropomyosin alpha-1 chain | Tropomyosin 1 alpha chain | M2 |
| O95407 | SL5070_76 | Tumor necrosis factor receptor superfamily member 6B | DcR3 | M2 |
| Q9NP60 | SL5082_51 | X-linked interleukin-1 receptor accessory protein-like 2 | IL-1 sR9 | M2 |
| Q9UHF4 | SL5085_18 | Interleukin-20 receptor subunit alpha | IL-20 Ra | M2 |
| Q86YT9 | SL5094_62 | Junctional adhesion molecule-like | JAML1 | M2 |
| Q99706 | SL5095_21 | Killer cell immunoglobulin-like receptor 2DL4 | KI2L4 | M2 |
| P43630 | SL5096_51 | Killer cell immunoglobulin-like receptor 3DL2 | KI3L2 | M2 |
| Q9NZS2 | SL5098_79 | Killer cell lectin-like receptor subfamily F member 1 | KLRF1 | M2 |
| Q29980 | SL5102_55 | MHC class I polypeptide-related sequence B | MICB | M2 |
| Q8TD46 | SL5103_30 | Cell surface glycoprotein CD200 receptor 1 | MO2R1 | M2 |
| Q04721 | SL5106_52 | Neurogenic locus notch homolog protein 2 | NOTC2 | M2 |
| P16471 | SL5114_65 | Prolactin receptor | Prolactin Receptor | M2 |
| Q96MS0 | SL5117_14 | Roundabout homolog 3 | ROBO3 | M2 |
| P37173 | SL5133_17 | TGF-beta receptor type-2 | TGF-b R II | M2 |
| Q14397 | SL5223_59 | Glucokinase regulatory protein | GCKR | M2 |
| Q15118 | SL5227_60 | [Pyruvate dehydrogenase (acetyl-transferring)] kinase isozyme 1, mitochondrial | PDK1 | M2 |
| P04035 | SL5230_99 | 3-hydroxy-3-methylglutaryl-coenzyme A reductase | HMGR | M2 |
| P20393 | SL5236_2 | Nuclear receptor subfamily 1 group D member 1 | NR1D1 | M2 |
| O94768 | SL5249_31 | Serine/threonine-protein kinase 17B | DRAK2 | M2 |
| P54750 | SL5253_1 | Calcium/calmodulin-dependent 3',5'-cyclic nucleotide phosphodiesterase 1A | PDE1A | M2 |
| P51512 | SL5268_49 | Matrix metalloproteinase-16 | MMP-16 | M2 |
| P00740 | SL5307_12 | Coagulation factor IXab | Coagulation Factor IXab | M2 |
| P16870 | SL5343_74 | Carboxypeptidase E | CBPE | M2 |
| Q8WWK9 | SL5345_51 | Cytoskeleton-associated protein 2 | CKAP2 | M2 |
| P14635 | SL5347_59 | G2/mitotic-specific cyclin-B1 | Cyclin B1 | M2 |
| P18510 | SL5353_89 | Interleukin-1 receptor antagonist protein | IL-1Ra | M2 |
| Q8N0W4 | SL5357_60 | Neuroligin-4, X-linked | NLGNX | M2 |
| P11309 | SL5359_65 | Serine/threonine-protein kinase pim-1 | PIM1 | M2 |
| P31751 | SL5360_9 | RAC-beta serine/threonine-protein kinase | PKB beta | M2 |
| P48357 | SL5400_52 | Leptin receptor, soluble | sLeptin R | M2 |
| P48788 | SL5440_26 | Troponin I, fast skeletal muscle | Troponin I, skeletal, fast twitch | M2 |
| P07306 | SL5452_71 | Asialoglycoprotein receptor 1 | ASGR1 | M2 |
| Q96KN2 | SL5456_59 | Beta-Ala-His dipeptidase | CNDP1 | M2 |
| Q96B86 | SL5483_1 | Repulsive guidance molecule A | RGMA | M2 |
| Q9NQ25 | SL5487_7 | SLAM family member 7 | SLAF7 | M2 |
| P62306 | SL5494_52 | Small nuclear ribonucleoprotein F | RUXF | M2 |
| Q08830 | SL5581_28 | Fibrinogen-like protein 1 | FGL1 | M2 |
| Q96PD5 | SL5601_2 | N-acetylmuramoyl-L-alanine amidase | PGRP-L | M2 |
| Q5GAN6 | SL5602_62 | Inactive ribonuclease-like protein 10 | RNS10 | M2 |
| O00587 | SL5605_77 | Beta-1,3-N-acetylglucosaminyltransferase manic fringe | MFNG | M2 |
| Q5VUB5 | SL5610_32 | Protein FAM171A1 | CJ038 | M2 |
| Q9GZY6 | SL5613_75 | Linker for activation of T-cells family member 2 | NTAL | M2 |
| P06850 | SL5614_44 | Corticoliberin | CRH | M2 |
| Q8TDQ1 | SL5623_11 | CMRF35-like molecule 1 | CLM1 | M2 |
| Q9NS98 | SL5628_21 | Semaphorin-3G | SEM3G | M2 |
| P26715 | SL5629_58 | NKG2-A/NKG2-B type II integral membrane protein | NKG2A | M2 |
| A4D1T9 | SL5653_23 | Probable inactive serine protease 37 | PRS37 | M2 |
| P08294 | SL5660_51 | Extracellular superoxide dismutase [Cu-Zn] | SOD3 | M2 |
| P10720 | SL5663_18 | Platelet factor 4 variant | PF4V | M2 |
| Q8N104 | SL5664_57 | Beta-defensin 106 | D106A | M2 |
| Q13214 | SL5667_3 | Semaphorin-3B | SEM3B | M2 |
| Q6EMK4 | SL5682_13 | Vasorin | VASN | M2 |
| P05013 | SL5714_88 | Interferon alpha-6 | IFNA6 | M2 |
| P42785 | SL5722_78 | Lysosomal Pro-X carboxypeptidase | Prolylcarboxypeptidase | M2 |
| P51460 | SL5723_4 | Insulin-like 3 | INSL3 | M2 |
| Q6UWP8 | SL5724_58 | Suprabasin | SBSN | M2 |
| Q6PDA7 | SL5726_49 | Sperm-associated antigen 11A | SG11A | M2 |
| P60985 | SL5739_75 | Keratinocyte differentiation-associated protein | KTDAP | M2 |
| Q9Y6N7 | SL5740_17 | Roundabout homolog 1 | ROBO1 | M2 |
| O43692 | SL5745_64 | Peptidase inhibitor 15 | PI15 | M2 |
| Q9BXP8 | SL5756_66 | Pappalysin-2 | PAPP2 | M2 |
| Q9UKQ9 | SL5758_49 | Kallikrein-9 | kallikrein 9 | M2 |
| P02771 | SL5792_8 | alpha-Fetoprotein | AFP | M2 |
| P01024 | SL5803_24 | Complement C3d fragment | C3d | M2 |
| P39905 | SL5822_22 | Glial cell line-derived neurotrophic factor | GDNF | M2 |
| P05089 | SL5867_60 | Arginase-1 | ARGI1 | M2 |
| P07492 | SL5897_58 | Gastrin-releasing peptide | Gastrin-releasing peptide | M2 |
| O95389 | SL5927_4 | WNT1-inducible-signaling pathway protein 3 | WISP-3 | M2 |
| P01375 | SL5936_53 | Tumor necrosis factor | TNF-a | M2 |
| P40225 | SL5947_90 | Thrombopoietin | Tpo | M2 |
| Q6E0U4 | SL5963_9 | Dermokine | Dermokine | M2 |
| P30042 | SL5981_6 | ES1 protein homolog, mitochondrial | ES1 | M2 |
| O95157 | SL6054_6 | Neurexophilin-3 | NXPH3 | M2 |
| Q8TE99 | SL6079_59 | 2-phosphoxylose phosphatase 1 | ACPL2 | M2 |
| P04637 | SL6123_69 | Cellular tumor antigen p53 | p53 | M2 |
| P04637 | SL6168_11 | Cellular tumor antigen p53 R175H mutant | p53 R175H | M2 |
| P01569 | SL6210_100 | Interferon alpha-5 | IFNA5 | M2 |
| Q16661 | SL6223_5 | Guanylate cyclase activator 2B | GUC2B | M2 |
| O76061 | SL6231_46 | Stanniocalcin-2 | STC2 | M2 |
| Q92692 | SL6245_4 | Nectin-2 | Nectin-2 | M2 |
| Q9NRM6 | SL6262_14 | Interleukin-17 receptor B | IL-17B R | M2 |
| P09958 | SL6276_16 | Furin | Furin | M2 |
| Q8IX19 | SL6283_60 | Mast cell-expressed membrane protein 1 | MCEM1 | M2 |
| Q6P4A8 | SL6315_58 | Phospholipase B-like 1 | PLBL1 | M2 |
| Q92874 | SL6324_11 | Deoxyribonuclease-1-like 2 | DNSL2 | M2 |
| P36268 | SL6334_9 | Inactive gamma-glutamyltranspeptidase 2 | GGT2 | M2 |
| P40259 | SL6351_55 | B-cell antigen receptor complex-associated protein beta chain | CD79B | M2 |
| Q6MZM0 | SL6354_13 | Hephaestin-like protein 1 | HPHL1 | M2 |
| Q7Z7B8 | SL6360_7 | Beta-defensin 128 | DB128 | M2 |
| Q9H1Z8 | SL6362_6 | Augurin | AUGN | M2 |
| O75398 | SL6369_82 | Deformed epidermal autoregulatory factor 1 homolog | DEAF1 | M2 |
| P80370 | SL6373_54 | Protein delta homolog 1 | DLK1 | M2 |
| Q8NBI6 | SL6375_75 | Xyloside xylosyltransferase 1 | XXLT1 | M2 |
| Q86SI9 | SL6378_2 | Protein CEI | CEI | M2 |
| Q96T91 | SL6395_58 | Glycoprotein hormone alpha-2 | GPHA2 | M2 |
| Q8IZN7 | SL6399_52 | Beta-defensin 107 | D107A | M2 |
| Q9UKJ1 | SL6402_8 | Paired immunoglobulin-like type 2 receptor alpha isoform FDF03-deltaTM | PILRA isoform FDF03-deltaTM | M2 |
| Q14641 | SL6410_26 | Early placenta insulin-like peptide | INSL4 | M2 |
| Q30KP9 | SL6411_58 | Beta-defensin 135 | DB135 | M2 |
| Q86UD1 | SL6414_8 | Out at first protein homolog | OAF | M2 |
| Q86XP6 | SL6416_8 | Gastrokine-2 | GKN2 | M2 |
| Q5GAN3 | SL6424_2 | Probable inactive ribonuclease-like protein 13 | RNS13 | M2 |
| Q8N3H0 | SL6430_36 | Protein FAM19A2 | F19A2 | M2 |
| Q9UHG3 | SL6431_68 | Prenylcysteine oxidase 1 | PCYOX | M2 |
| Q96MK3 | SL6433_57 | Pseudokinase FAM20A | FA20A | M2 |
| Q13361 | SL6440_31 | Microfibrillar-associated protein 5 | MFAP5 | M2 |
| P26022 | SL6447_73 | Pentraxin-related protein PTX3 | PTX3 | M2 |
| Q99985 | SL6448_36 | Semaphorin-3C | Sema E | M2 |
| Q00889 | SL6456_17 | Pregnancy-specific beta-1-glycoprotein 6 | PSG6 | M2 |
| Q86UU9 | SL6468_37 | Tachykinin-4 | TKN4 | M2 |
| O95868 | SL6469_62 | Lymphocyte antigen 6 complex locus protein G6d | LY66D | M2 |
| Q9NP55 | SL6473_55 | BPI fold-containing family A member 1 | PLUNC | M2 |
| Q66K79 | SL6493_9 | Carboxypeptidase Z | CBPZ | M2 |
| P80370 | SL6496_60 | Protein delta homolog 1 | DLK1 | M2 |
| O15537 | SL6497_10 | Retinoschisin | XLRS1 | M2 |
| Q8TEB7 | SL6510_56 | E3 ubiquitin-protein ligase RNF128 | RN128 | M2 |
| Q96LR4 | SL6511_17 | Protein FAM19A4 | F19A4 | M2 |
| Q9UBU3 | SL6518_85 | Appetite-regulating hormone | ghrelin | M2 |
| Q7L0X0 | SL6527_1 | TLR4 interactor with leucine rich repeats | TRIL | M2 |
| Q9UBQ6 | SL6528_95 | Exostosin-like 2 | EXTL2 | M2 |
| Q8IYS2 | SL6538_90 | Uncharacterized protein KIAA2013 | K2013 | M2 |
| P81277 | SL6543_182 | Prolactin-releasing peptide | PRRP | M2 |
| Q92832 | SL6544_33 | Protein kinase C-binding protein NELL1 | NELL1 | M2 |
| P04156 | SL6545_58 | Major prion protein | PRIO | M2 |
| O60235 | SL6547_83 | Transmembrane protease serine 11D | TM11D | M2 |
| Q8IW75 | SL6551_94 | Serpin A12 | Vaspin | M2 |
| Q9UJA9 | SL6556_5 | Ectonucleotide pyrophosphatase/phosphodiesterase family member 5 | ENPP5 | M2 |
| Q9Y6Z7 | SL6558_5 | Collectin-10 | COL10 | M2 |
| P0DMV8 | SL6563_78 | Heat shock 70 kDa protein 1A | HSP 70 | M2 |
| Q8N4C9 | SL6571_75 | Uncharacterized protein C17orf78 | CQ078 | M2 |
| Q86VH4 | SL6572_10 | Leucine-rich repeat transmembrane neuronal protein 4 | LRRT4 | M2 |
| Q93070 | SL6576_1 | Ecto-ADP-ribosyltransferase 4 | ART4 | M2 |
| Q9H2S6 | SL6578_29 | Tenomodulin | TNMD | M2 |
| Q9NPB0 | SL6594_64 | SAYSvFN domain-containing protein 1 | SMDC1 | M2 |
| Q8TBP5 | SL6597_24 | Membrane protein FAM174A | TM157 | M2 |
| Q8J025 | SL6599_5 | Protein APCDD1 | APCD1 | M2 |
| Q8NC24 | SL6600_70 | RELT-like protein 2 | RELL2 | M2 |
| Q8TB73 | SL6604_59 | Protein NDNF | CD031 | M2 |
| Q6DN72 | SL6617_12 | Fc receptor-like protein 6 | FCRL6 | M2 |
| Q96FE5 | SL6620_82 | Leucine-rich repeat and immunoglobulin-like domain-containing nogo receptor-interacting protein 1 | LIGO1 | M2 |
| P54315 | SL6627_25 | Inactive pancreatic lipase-related protein 1 | LIPR1 | M2 |
| B0FP48 | SL6633_43 | Uroplakin-3b-like protein | UPK3L | M2 |
| Q08722 | SL6653_58 | Leukocyte surface antigen CD47 | CD47 | M2 |
| P02786 | SL6895_1 | Transferrin receptor protein 1 | TR | M2 |
| Q86XE3 | SL6912_6 | Calcium uptake protein 3, mitochondrial | EFHA2 | M2 |
| Q6UWJ8 | SL6915_2 | CD164 sialomucin-like 2 protein | C16L2 | M2 |
| P06307 | SL6918_183 | Cholecystokinin | CCKN | M2 |
| Q6UXV0 | SL6920_1 | GDNF family receptor alpha-like | GFRAL | M2 |
| O60512 | SL6921_24 | Beta-1,4-galactosyltransferase 3 | B4GT3 | M2 |
| P08648 | SL6932_42 | Integrin alpha-5 | ITA5 | M2 |
| Q9BQ48 | SL6933_20 | 39S ribosomal protein L34, mitochondrial | RM34 | M2 |
| Q9HDC5 | SL6940_18 | Junctophilin-1 | JPH1 | M2 |
| Q8NBK3 | SL6941_11 | Sulfatase-modifying factor 1 | SUMF1 | M2 |
| Q13596 | SL6955_68 | Sorting nexin-1 | SNX1 | M2 |
| Q6UXB1 | SL6961_14 | Insulin growth factor-like family member 3 | IGFL3 | M2 |
| Q9UHC6 | SL6965_19 | Contactin-associated protein-like 2 | CNTP2 | M2 |
| P61266 | SL6966_144 | Syntaxin-1B | STX1B | M2 |
| Q8TCV5 | SL6969_14 | WAP four-disulfide core domain protein 5 | WFDC5 | M2 |
| P04233 | SL6974_6 | HLA class II histocompatibility antigen gamma chain | HG2A | M2 |
| O95295 | SL6975_52 | SNARE-associated protein Snapin | SNAPN | M2 |
| Q969P0 | SL6984_6 | Immunoglobulin superfamily member 8 | IGSF8 | M2 |
| Q9Y662 | SL6986_17 | Heparan sulfate glucosamine 3-O-sulfotransferase 3B1 | HS3SB | M2 |
| Q9UK00 | SL6994_19 | Uncharacterized protein C3orf18 | CC018 | M2 |
| O75023 | SL7015_8 | Leukocyte immunoglobulin-like receptor subfamily B member 5 | LIRB5 | M2 |
| Q9UJK0 | SL7018_10 | Ribosome biogenesis protein TSR3 homolog | TSR3 | M2 |
| O75077 | SL7049_2 | Disintegrin and metalloproteinase domain-containing protein 23 | ADAM 23 | M2 |
| Q96HE7 | SL7060_2 | ERO1-like protein alpha | ERO1A | M2 |
| Q9BV40 | SL7064_2 | Vesicle-associated membrane protein 8 | VAMP8 | M2 |
| Q96NA8 | SL7069_9 | t-SNARE domain-containing protein 1 | TSNA1 | M2 |
| O00481 | SL7081_2 | Butyrophilin subfamily 3 member A1 | BT3A1 | M2 |
| Q6ZMB0 | SL7082_2 | UDP-GlcNAc:betaGal beta-1,3-N-acetylglucosaminyltransferase 6 | B3GN6 | M2 |
| Q8NBI3 | SL7084_1 | Draxin | DRAXI | M2 |
| Q8N807 | SL7092_7 | Protein disulfide-isomerase-like protein of the testis | PDILT | M2 |
| Q9Y6F8 | SL7097_8 | Testis-specific chromodomain protein Y 1 | CDY1 | M2 |
| Q8N475 | SL7099_33 | Follistatin-related protein 5 | FSTL5 | M2 |
| Q8IYS5 | SL7116_31 | Osteoclast-associated immunoglobulin-like receptor | OSCAR | M2 |
| O75830 | SL7117_21 | Serpin I2 | SPI2 | M2 |
| O43581 | SL7121_2 | Synaptotagmin-7 | SYT7 | M2 |
| Q9HBE4 | SL7124_18 | Interleukin-21 | IL-21 | M2 |
| Q86Z23 | SL7132_55 | Complement C1q-like protein 4 | C1QL4 | M2 |
| Q06033 | SL7145_1 | Inter-alpha-trypsin inhibitor heavy chain H3 | ITIH3 | M2 |
| Q9NPE2 | SL7153_66 | Neugrin | NGRN | M2 |
| Q9UN74 | SL7157_22 | Protocadherin alpha-4 | PCDA4 | M2 |
| Q9NTK1 | SL7178_59 | Protein DEPP | DEPP | M2 |
| Q6UXH0 | SL7183_102 | Angiopoietin-like protein 8 | TD26 | M2 |
| Q7LGC8 | SL7189_55 | Carbohydrate sulfotransferase 3 | CHST3 | M2 |
| P05000 | SL7196_21 | Interferon omega-1 | IFN-w | M2 |
| Q9H3T2 | SL7202_107 | Semaphorin-6C | SEMA6C | M2 |
| P0DMV8 | SL7219_152 | Heat shock 70 kDa protein 1A | HSP 70 | M2 |
| Q8N4V1 | SL7225_51 | Membrane magnesium transporter 1 | MMGT1 | M2 |
| Q6ZMR5 | SL7231_37 | Transmembrane protease serine 11A | TM11A | M2 |
| Q8IYK4 | SL7234_12 | Procollagen galactosyltransferase 2 | GT252 | M2 |
| Q8IW00 | SL7242_14 | V-set and transmembrane domain-containing protein 4 | CJ072 | M2 |
| Q5T7V8 | SL7247_1 | RAB6-interacting golgin | GORAB | M2 |
| P61366 | SL7265_32 | Osteocrin | OSTN | M2 |
| O15270 | SL7267_2 | Serine palmitoyltransferase 2 | LCB2 | M2 |
| Q01484 | SL7624_19 | Ankyrin-2 | ANK2 | M2 |
| Q12907 | SL7638_30 | Vesicular integral-membrane protein VIP36 | Lectin, mannose-binding 2 | M2 |
| Q00872 | SL7648_9 | Myosin-binding protein C, slow-type | MYPC1 | M2 |
| O75379 | SL7732_45 | Vesicle-associated membrane protein 4 | VAMP4 | M2 |
| P32856 | SL7738_299 | Syntaxin-2 | STX2 | M2 |
| Q8N7C7 | SL7742_11 | RING finger protein 148 | RN148 | M2 |
| Q9NZ45 | SL7745_3 | CDGSH iron-sulfur domain-containing protein 1 | ZCD1 | M2 |
| Q9NX14 | SL7747_47 | NADH dehydrogenase [ubiquinone] 1 beta subcomplex subunit 11, mitochondrial | NDUBB | M2 |
| Q96HF1 | SL7751_121 | Secreted frizzled-related protein 2 | SARP-1 | M2 |
| Q8WXI8 | SL7752_31 | C-type lectin domain family 4 member D | CLC4D | M2 |
| P18084 | SL7755_37 | Integrin beta-5 | ITB5 | M2 |
| Q6UWE3 | SL7767_1 | Colipase-like protein 2 | CF126 | M2 |
| P43627 | SL7773_20 | Killer cell immunoglobulin-like receptor 2DL2 | KI2L2 | M2 |
| Q9NPF2 | SL7779_86 | Carbohydrate sulfotransferase 11 | CHSTB | M2 |
| Q15768 | SL7785_1 | Ephrin-B3 | Ephrin-B3 | M2 |
| Q9P0B6 | SL7797_11 | Coiled-coil domain-containing protein 167 | CC167 | M2 |
| O43916 | SL7803_4 | Carbohydrate sulfotransferase 1 | CHST1 | M2 |
| O95249 | SL7805_52 | Golgi SNAP receptor complex member 1 | GOS-28 | M2 |
| O94923 | SL7808_5 | D-glucuronyl C5-epimerase | GLCE | M2 |
| Q8WX77 | SL7815_49 | Insulin-like growth factor-binding protein-like 1 | IBPL1 | M2 |
| Q6ZN66 | SL7818_101 | Guanylate-binding protein 6 | GBP6 | M2 |
| Q13901 | SL7821_6 | Nuclear nucleic acid-binding protein C1D | C1D | M2 |
| Q9NWW9 | SL7822_11 | HRAS-like suppressor 2 | HRSL2 | M2 |
| Q9UJ37 | SL7823_22 | Alpha-N-acetylgalactosaminide alpha-2,6-sialyltransferase 2 | SIA7B | M2 |
| Q99954 | SL7838_27 | Submaxillary gland androgen-regulated protein 3A | SMR3A | M2 |
| Q9UQ72 | SL7846_44 | Pregnancy-specific beta-1-glycoprotein 11 | PSG11 | M2 |
| Q16769 | SL7849_3 | Glutaminyl-peptide cyclotransferase | Glutaminyl cyclase | M2 |
| Q96KJ9 | SL7850_1 | Cytochrome c oxidase subunit 4 isoform 2, mitochondrial | COX42 | M2 |
| Q86W74 | SL7851_30 | Ankyrin repeat domain-containing protein 46 | ANR46 | M2 |
| Q68D85 | SL7854_38 | Natural cytotoxicity triggering receptor 3 ligand 1 | NR3L1 | M2 |
| Q8WW52 | SL7856_51 | Protein FAM151A | F151A | M2 |
| Q5GLZ8 | SL7860_9 | Probable E3 ubiquitin-protein ligase HERC4 | HERC4 | M2 |
| Q9NYZ4 | SL7864_3 | Sialic acid-binding Ig-like lectin 8 | SIGL8 | M2 |
| Q9NSC7 | SL7867_154 | Alpha-N-acetylgalactosaminide alpha-2,6-sialyltransferase 1 | SIA7A | M2 |
| Q96PF2 | SL7873_32 | Testis-specific serine/threonine-protein kinase 2 | TSSK2 | M2 |
| P08684 | SL7879_12 | Cytochrome P450 3A4 | Cytochrome P450 3A4 | M2 |
| Q8N5Y8 | SL7881_244 | Mono [ADP-ribose] polymerase PARP16 | PAR16 | M2 |
| Q9Y2R0 | SL7888_58 | Cytochrome c oxidase assembly factor 3 homolog, mitochondrial | CCD56 | M2 |
| Q8N608 | SL7890_68 | Inactive dipeptidyl peptidase 10 | DPP10 | M2 |
| A2RU67 | SL7892_132 | Protein FAM234B | K1467 | M2 |
| Q8TCY5 | SL7895_108 | Melanocortin-2 receptor accessory protein | MRAP | M2 |
| Q8TC05 | SL7898_29 | Nuclear protein MDM1 | MDM1 | M2 |
| Q9P244 | SL7910_41 | Leucine-rich repeat and fibronectin type III domain-containing protein 1 | LRFN1 | M2 |
| P31151 | SL7916_10 | Protein S100-A7 | S100A7 | M2 |
| P09693 | SL7924_7 | T-cell surface glycoprotein CD3 gamma chain | CD3G | M2 |
| A6NFE2 | SL7925_18 | Single-pass membrane and coiled-coil domain-containing protein 2 | CL070 | M2 |
| Q9Y3B2 | SL7930_3 | Exosome complex component CSL4 | EXOS1 | M2 |
| Q8NBV8 | SL7932_23 | Synaptotagmin-8 | SYT8 | M2 |
| Q9ULP0 | SL7934_11 | Protein NDRG4 | NDRG4 | M2 |
| Q86UP6 | SL7943_16 | CUB and zona pellucida-like domain-containing protein 1 | CUZD1 | M2 |
| Q8N743 | SL7944_1 | Killer cell immunoglobulin-like receptor 3DL3 | KI3L3 | M2 |
| Q96N21 | SL7947_19 | AP-4 complex accessory subunit tepsin | AP4AT | M2 |
| Q6UXG8 | SL7950_142 | Butyrophilin-like protein 9 | BTNL9 | M2 |
| P19827 | SL7955_195 | Inter-alpha-trypsin inhibitor heavy chain H1 | ITI heavy chain H1 | M2 |
| Q9ULB5 | SL7959_34 | Cadherin-7 | CADH7 | M2 |
| P0C7L1 | SL7962_11 | Serine protease inhibitor Kazal-type 8 | ISK8 | M2 |
| Q96L58 | SL7981_230 | Beta-1,3-galactosyltransferase 6 | B3GT6 | M2 |
| Q9Y5F6 | SL7983_1 | Protocadherin gamma-C5 | PCDGM | M2 |
| Q6HA08 | SL7993_23 | Astacin-like metalloendopeptidase | ASTL | M2 |
| P49961 | SL7999_23 | Ectonucleoside triphosphate diphosphohydrolase 1 | CD39 | M2 |
| Q8N109 | SL8000_17 | Killer cell immunoglobulin-like receptor 2DL5A | KI2LA | M2 |
| Q9H3S3 | SL8002_27 | Transmembrane protease serine 5 | Spinesin | M2 |
| P07858 | SL8007_19 | Cathepsin B | Cathepsin B | M2 |
| O00198 | SL8008_28 | Activator of apoptosis harakiri | HRK | M2 |
| Q9NNZ3 | SL8016_19 | DnaJ homolog subfamily C member 4 | DNJC4 | M2 |
| Q96CU9 | SL8017_23 | FAD-dependent oxidoreductase domain-containing protein 1 | FXRD1 | M2 |
| Q6P1L8 | SL8021_59 | 39S ribosomal protein L14, mitochondrial | RM14 | M2 |
| P01225 | SL8036_75 | Follitropin subunit beta | FSHB | M2 |
| P55808 | SL8044_90 | Glycoprotein Xg | XG | M2 |
| Q8N743 | SL8045_3 | Killer cell immunoglobulin-like receptor 3DL3 | KI3L3 | M2 |
| P09382 | SL8046_9 | Galectin-1 | Galectin-1 | M2 |
| Q8TBM8 | SL8053_16 | DnaJ homolog subfamily B member 14 | DJB14 | M2 |
| P98153 | SL8055_33 | Integral membrane protein DGCR2/IDD | IDD | M2 |
| P40225 | SL8059_1 | Thrombopoietin | Tpo | M2 |
| Q6P995 | SL8061_102 | Protein FAM171B | F171B | M2 |
| Q9NVM1 | SL8062_15 | Protein eva-1 homolog B | F176B | M2 |
| P0C8F1 | SL8065_245 | Prostate and testis expressed protein 4 | PATE4 | M2 |
| Q86SS6 | SL8066_38 | Synaptotagmin-9 | SYT9 | M2 |
| Q9Y6H6 | SL8067_21 | Potassium voltage-gated channel subfamily E member 3 | KCNE3 | M2 |
| Q6NSJ0 | SL8068_43 | Uncharacterized family 31 glucosidase KIAA1161 | K1161 | M2 |
| Q9Y5E2 | SL8071_114 | Protocadherin beta-7 | PCDB7 | M2 |
| O43567 | SL8087_250 | E3 ubiquitin-protein ligase RNF13 | RNF13 | M2 |
| Q8IVY1 | SL8088_56 | Type III endosome membrane protein TEMP | CA210 | M2 |
| P22736 | SL8089_173 | Nuclear receptor subfamily 4 group A member 1 | NR4A1 | M2 |
| Q6NSJ0 | SL8093_13 | Uncharacterized family 31 glucosidase KIAA1161 | K1161 | M2 |
| P24557 | SL8098_37 | Thromboxane-A synthase | THAS | M2 |
| Q13651 | SL8104_21 | Interleukin-10 receptor subunit alpha | IL-10 Ra | M2 |
| P43307 | SL8106_15 | Translocon-associated protein subunit alpha | SSRA | M2 |
| Q8WY98 | SL8107_12 | Transmembrane protein 234 | TM234 | M2 |
| Q6ZTQ4 | SL8222_49 | Cadherin-related family member 3 | CDHR3 | M2 |
| P17948 | SL8231_122 | Vascular endothelial growth factor receptor 1 | VEGF sR1 | M2 |
| Q6P988 | SL8252_2 | Palmitoleoyl-protein carboxylesterase NOTUM | NOTUM | M2 |
| Q8WZA1 | SL8253_2 | Protein O-linked-mannose beta-1,2-N-acetylglucosaminyltransferase 1 | PMGT1 | M2 |
| P27105 | SL8261_51 | Erythrocyte band 7 integral membrane protein | STOM | M2 |
| Q8TAI1 | SL8263_64 | TYMS opposite strand protein | CR056 | M2 |
| P59901 | SL8299_66 | Leukocyte immunoglobulin-like receptor subfamily A member 4 | LIRA4 | M2 |
| O00300 | SL8304_50 | Tumor necrosis factor receptor superfamily member 11B | OPG | M2 |
| O14668 | SL8306_54 | Transmembrane gamma-carboxyglutamic acid protein 1 | TMG1 | M2 |
| Q8N690 | SL8315_5 | Beta-defensin 119 | DB119 | M2 |
| Q7Z699 | SL8318_13 | Sprouty-related, EVH1 domain-containing protein 1 | SPRE1 | M2 |
| Q8N446 | SL8321_27 | Zinc finger protein 843 | ZN843 | M2 |
| P08319 | SL8325_37 | Alcohol dehydrogenase 4 | ADH4 | M2 |
| Q8IYS0 | SL8336_267 | GRAM domain-containing protein 1C | GRM1C | M2 |
| Q30KQ9 | SL8340_9 | Beta-defensin 110 | DB110 | M2 |
| Q96PQ1 | SL8352_26 | Sialic acid-binding Ig-like lectin 12 | SIG12 | M2 |
| P01178 | SL8356_88 | Oxytocin-neurophysin 1 | NEU1 | M2 |
| Q9H7X2 | SL8366_19 | Uncharacterized protein C1orf115 | CA115 | M2 |
| P08138 | SL8374_5 | Tumor necrosis factor receptor superfamily member 16 | NGF R | M2 |
| O95751 | SL8378_3 | Protein LDOC1 | LDOC1 | M2 |
| P80370 | SL8380_244 | Protein delta homolog 1 | DLK1 | M2 |
| Q8IW41 | SL8382_47 | MAP kinase-activated protein kinase 5 | MAPK5 | M2 |
| Q9UBP0 | SL8388_24 | Spastin | Spastin | M2 |
| Q0VAA2 | SL8389_8 | Leucine-rich repeat-containing protein 74A | CN16B | M2 |
| P24310 | SL8390_25 | Cytochrome c oxidase subunit 7A1, mitochondrial | CX7A1 | M2 |
| Q30KQ5 | SL8391_12 | Beta-defensin 115 | DB115 | M2 |
| P10153 | SL8394_56 | Non-secretory ribonuclease | RNase 2 | M2 |
| O94768 | SL8399_6 | Serine/threonine-protein kinase 17B | DRAK2 | M2 |
| P07492 | SL8400_74 | Gastrin-releasing peptide | Gastrin-releasing peptide | M2 |
| Q9H3K6 | SL8404_102 | BolA-like protein 2 | BolA-like protein 2 | M2 |
| P05019 | SL8406_17 | Insulin-like growth factor I | IGF-I | M2 |
| Q9UBU3 | SL8447_11 | Appetite-regulating hormone | ghrelin | M2 |
| P09238 | SL8479_4 | Stromelysin-2 | MMP-10 | M2 |
| P41159 | SL8484_24 | Leptin | Leptin | M2 |
| Q6E0U4 | SL8535_102 | Dermokine | Dermokine | M2 |
| Q9H6L5 | SL8556_5 | Protein FAM134B | F134B | M2 |
| Q07954 | SL8601_167 | Low-density lipoprotein receptor-related protein 1, soluble | sLRP1 | M2 |
| Q8TEB7 | SL8633_18 | E3 ubiquitin-protein ligase RNF128 | RN128 | M2 |
| Q86VH4 | SL8646_61 | Leucine-rich repeat transmembrane neuronal protein 4 | LRRT4 | M2 |
| Q9NNZ3 | SL8653_132 | DnaJ homolog subfamily C member 4 | DNJC4 | M2 |
| Q9NRN5 | SL8660_5 | Olfactomedin-like protein 3 | OLFL3 | M2 |
| O15173 | SL8681_93 | Membrane-associated progesterone receptor component 2 | PGRC2 | M2 |
| Q8NCW6 | SL8700_325 | Polypeptide N-acetylgalactosaminyltransferase 11 | GLT11 | M2 |
| P04233 | SL8748_45 | HLA class II histocompatibility antigen gamma chain | HG2A | M2 |
| P15813 | SL8749_194 | Antigen-presenting glycoprotein CD1d | CD1D | M2 |
| Q9BTE7 | SL8760_10 | DCN1-like protein 5 | DCNL5 | M2 |
| P42892 | SL8767_44 | Endothelin-converting enzyme 1 | Endothelin-converting enzyme 1 | M2 |
| Q9NT22 | SL8773_172 | EMILIN-3 | EMIL3 | M2 |
| Q8N5W8 | SL8775_61 | Protein FAM24B | FA24B | M2 |
| O75477 | SL8776_10 | Erlin-1 | ERLN1 | M2 |
| Q9P2E7 | SL8780_2 | Protocadherin-10 | PCD10 | M2 |
| Q96C12 | SL8785_1 | Armadillo repeat-containing protein 5 | ARMC5 | M2 |
| Q6P995 | SL8786_6 | Protein FAM171B | F171B | M2 |
| P16444 | SL8794_13 | Dipeptidase 1 | DPEP1 | M2 |
| P02786 | SL8795_48 | Transferrin receptor protein 1 | TR | M2 |
| Q9P218 | SL8804_39 | Collagen alpha-1(XX) chain | COKA1 | M2 |
| Q13596 | SL8807_13 | Sorting nexin-1 | SNX1 | M2 |
| P10599 | SL8813_160 | Thioredoxin | Thioredoxin | M2 |
| P38484 | SL8818_13 | Interferon gamma receptor 2 | INGR2 | M2 |
| Q9UKJ1 | SL8825_4 | Paired immunoglobulin-like type 2 receptor alpha | PILRA | M2 |
| Q10589 | SL8832_55 | Bone marrow stromal antigen 2 | BST-2 | M2 |
| O75431 | SL8839_4 | Metaxin-2 | Metaxin-2 | M2 |
| P09871 | SL8840_61 | Complement C1s subcomponent | C1s | M2 |
| Q8IYS0 | SL8842_16 | GRAM domain-containing protein 1C | GRM1C | M2 |
| Q9Y2G1 | SL8843_34 | Myelin regulatory factor | MRF | M2 |
| Q8TAQ9 | SL8852_10 | SUN domain-containing protein 3 | SUN3 | M2 |
| O75503 | SL8874_53 | Ceroid-lipofuscinosis neuronal protein 5 | CLN5 | M2 |
| P58401 | SL8876_51 | Neurexin-2-beta | NRX2B | M2 |
| P58658 | SL8877_22 | Protein eva-1 homolog C | F176C | M2 |
| A6NGZ8 | SL8888_33 | Small integral membrane protein 9 | CX068 | M2 |
| Q9ULG6 | SL8889_5 | Cell cycle progression protein 1 | CCPG1 | M2 |
| Q14DG7 | SL8890_9 | Transmembrane protein 132B | T132B | M2 |
| P09669 | SL8903_1 | Cytochrome c oxidase subunit 6C | COX6C | M2 |
| Q13586 | SL8916_32 | Stromal interaction molecule 1 | STIM1 | M2 |
| Q9NYX4 | SL8924_55 | Neuron-specific vesicular protein calcyon | CALY | M2 |
| Q7LG56 | SL8925_25 | Ribonucleoside-diphosphate reductase subunit M2 B | RIR2B | M2 |
| A6H8Y1 | SL8929_7 | Transcription factor TFIIIB component B'' homolog | BDP1 | M2 |
| P27469 | SL8931_124 | G0/G1 switch protein 2 | G0S2 | M2 |
| O75354 | SL8932_1 | Ectonucleoside triphosphate diphosphohydrolase 6 | ENTP6 | M2 |
| Q9H013 | SL8948_13 | Disintegrin and metalloproteinase domain-containing protein 19 | ADA19 | M2 |
| Q6UVK1 | SL8951_162 | Chondroitin sulfate proteoglycan 4 | CSPG4 | M2 |
| Q68CQ7 | SL8955_60 | Glycosyltransferase 8 domain-containing protein 1 | GL8D1 | M2 |
| Q9BV47 | SL8967_6 | Dual specificity protein phosphatase 26 | DUS26 | M2 |
| Q8IVH8 | SL8978_30 | Mitogen-activated protein kinase kinase kinase kinase 3 | M4K3 | M2 |
| Q8NBJ4 | SL8983_7 | Golgi membrane protein 1 | GOLM1 | M2 |
| Q96JX3 | SL8985_13 | Protein SERAC1 | SRAC1 | M2 |
| Q8IWY4 | SL8989_40 | Signal peptide, CUB and EGF-like domain-containing protein 1 | SCUB1 | M2 |
| Q86U17 | SL9002_36 | Serpin A11 | SPA11 | M2 |
| O14668 | SL9008_6 | Transmembrane gamma-carboxyglutamic acid protein 1 | TMG1 | M2 |
| O94906 | SL9012_1 | Pre-mRNA-processing factor 6 | PRP6 | M2 |
| Q9Y2Y8 | SL9015_1 | Proteoglycan 3 | PRG3 | M2 |
| P55055 | SL9016_12 | Oxysterols receptor LXR-beta | NR1H2 | M2 |
| Q9P2E7 | SL9018_38 | Protocadherin-10 | PCD10 | M2 |
| Q8NFZ4 | SL9027_10 | Neuroligin-2 | NLGN2 | M2 |
| Q9P2W9 | SL9037_1 | Syntaxin-18 | STX18 | M2 |
| O76095 | SL9038_12 | Protein JTB | JTB | M2 |
| Q9UBB9 | SL9043_7 | Tuftelin-interacting protein 11 | TFP11 | M2 |
| P52943 | SL9053_16 | Cysteine-rich protein 2 | CRIP2 | M2 |
| P0CAP1 | SL9055_81 | Myocardial zonula adherens protein | MYZAP | M2 |
| Q92565 | SL9059_14 | Rap guanine nucleotide exchange factor 5 | RPGF5 | M2 |
| Q96IV6 | SL9074_6 | Fatty acid hydroxylase domain-containing protein 2 | CE004 | M2 |
| P01210 | SL9076_25 | Proenkephalin-A | PENK | M2 |
| Q99726 | SL9081_39 | Zinc transporter 3 | ZNT3 | M2 |
| Q5TEA6 | SL9082_25 | Protein sel-1 homolog 2 | SE1L2 | M2 |
| Q8WXH2 | SL9089_77 | Junctophilin-3 | JPH3 | M2 |
| O95841 | SL9092_33 | Angiopoietin-related protein 1 | ANGL1 | M2 |
| O00445 | SL9099_19 | Synaptotagmin-5 | SYT5 | M2 |
| Q5DID0 | SL9114_84 | Uromodulin-like 1 | UROL1 | M2 |
| A8MVW5 | SL9116_28 | HEPACAM family member 2 | HECA2 | M2 |
| P22894 | SL9172_69 | Neutrophil collagenase | MMP-8 | M2 |
| O60469 | SL9175_48 | Down syndrome cell adhesion molecule | DSCAM | M2 |
| P15941 | SL9176_3 | Mucin-1 | MUC1 | M2 |
| P38484 | SL9180_6 | Interferon gamma receptor 2 | INGR2 | M2 |
| Q07954 | SL9182_3 | Low-density lipoprotein receptor-related protein 1, soluble | sLRP1 | M2 |
| Q07325 | SL9188_119 | C-X-C motif chemokine 9 | MIG | M2 |
| P47929 | SL9196_8 | Galectin-7 | Galectin-7 | M2 |
| O00182 | SL9197_4 | Galectin-9 | LEG9 | M2 |
| P60604 | SL9199_6 | Ubiquitin-conjugating enzyme E2 G2 | UB2G2 | M2 |
| O95825 | SL9207_60 | Quinone oxidoreductase-like protein 1 | QORL1 | M2 |
| Q9UBX1 | SL9212_22 | Cathepsin F | CATF | M2 |
| Q9UHP3 | SL9215_117 | Ubiquitin carboxyl-terminal hydrolase 25 | UBP25 | M2 |
| P54753 | SL9220_7 | Ephrin type-B receptor 3 | EPHB3 | M2 |
| Q71RG4 | SL9226_6 | Transmembrane and ubiquitin-like domain-containing protein 2 | TMUB2 | M2 |
| P11117 | SL9237_54 | Lysosomal acid phosphatase | Lysosomal acid phosphatase | M2 |
| Q9P1W8 | SL9241_40 | Signal-regulatory protein gamma | SIRPG | M2 |
| Q9Y2E5 | SL9251_28 | Epididymis-specific alpha-mannosidase | MA2B2 | M2 |
| Q9P0M4 | SL9255_5 | Interleukin-17C | IL-17C | M2 |
| Q9UI42 | SL9267_2 | Carboxypeptidase A4 | CBPA4 | M2 |
| Q13586 | SL9271_101 | Stromal interaction molecule 1 | STIM1 | M2 |
| Q7Z7M8 | SL9297_12 | UDP-GlcNAc:betaGal beta-1,3-N-acetylglucosaminyltransferase 8 | B3GN8 | M2 |
| Q8TAE8 | SL9302_90 | Growth arrest and DNA damage-inducible proteins-interacting protein 1 | G45IP | M2 |
| P10966 | SL9310_2 | T-cell surface glycoprotein CD8 beta chain | CD8B | M2 |
| P25311 | SL9312_8 | Zinc-alpha-2-glycoprotein | AZGP1 | M2 |
| Q15238 | SL9314_9 | Pregnancy-specific beta-1-glycoprotein 5 | PSG5 | M2 |
| Q8N386 | SL9317_4 | Leucine-rich repeat-containing protein 25 | LRC25 | M2 |
| Q00887 | SL9335_28 | Pregnancy-specific beta-1-glycoprotein 9 | PSG9 | M2 |
| P20366 | SL9337_43 | Protachykinin-1 | TKN1 | M2 |
| Q6MZW2 | SL9350_3 | Follistatin-related protein 4 | FSTL4 | M2 |
| Q8WUE5 | SL9363_11 | Cancer/testis antigen 55 | CX048 | M2 |
| Q9HBE5 | SL9366_54 | Interleukin-21 receptor | IL-21 sR | M2 |
| Q92820 | SL9370_69 | Gamma-glutamyl hydrolase | GGH | M2 |
| Q8N539 | SL9378_6 | Fibrinogen C domain-containing protein 1 | FBCD1 | M2 |
| Q96HG1 | SL9379_248 | Small integral membrane protein 10 | CX069 | M2 |
| Q15782 | SL9383_24 | Chitinase-3-like protein 2 | CH3L2 | M2 |
| P49913 | SL9384_17 | Cathelicidin antimicrobial peptide | Cathelicidin peptide | M2 |
| P10253 | SL9385_4 | Lysosomal alpha-glucosidase | GAA | M2 |
| Q9UBC7 | SL9398_30 | Galanin-like peptide | GALP | M2 |
| P14384 | SL9416_77 | Carboxypeptidase M | CBPM | M2 |
| Q14129 | SL9444_70 | Protein DGCR6 | DGCR6 | M2 |
| P51636 | SL9457_3 | Caveolin-2 | CAV2 | M2 |
| Q12907 | SL9468_8 | Vesicular integral-membrane protein VIP36 | Lectin, mannose-binding 2 | M2 |
| Q8N687 | SL9486_13 | Beta-defensin 125 | DB125 | M2 |
| Q8N8J7 | SL9490_3 | Uncharacterized protein C4orf32 | CD032 | M2 |
| O14791 | SL9506_10 | Apolipoprotein L1 | Apo L1 | M2 |
| Q9NQX7 | SL9523_34 | Integral membrane protein 2C | ITM2C | M2 |
| Q6PKC3 | SL9573_108 | Thioredoxin domain-containing protein 11 | TXD11 | M2 |
| Q8N9I0 | SL9577_26 | Synaptotagmin-2 | SYT2 | M2 |
| O94906 | SL9581_4 | Pre-mRNA-processing factor 6 | PRP6 | M2 |
| Q9H6B4 | SL9585_80 | CXADR-like membrane protein | ACAM | M2 |
| O14548 | SL9590_10 | Cytochrome c oxidase subunit 7A-related protein, mitochondrial | COX7R | M2 |
| P51512 | SL9719_145 | Matrix metalloproteinase-16 | MMP-16 | M2 |
| Q8N2G8 | SL9730_22 | GH3 domain-containing protein | GHDC | M2 |
| P40200 | SL9735_44 | T-cell surface protein tactile | TACT | M2 |
| Q0D2K3 | SL9767_22 | Protein ripply1 | RIPP1 | M2 |
| Q8NFZ4 | SL9772_153 | Neuroligin-2 | NLGN2 | M2 |
| Q8NC42 | SL9773_15 | E3 ubiquitin-protein ligase RNF149 | RN149 | M2 |
| Q9ULK6 | SL9774_59 | RING finger protein 150 | RN150 | M2 |
| P46695 | SL9786_310 | Radiation-inducible immediate-early gene IEX-1 | IEX1 | M2 |
| O95169 | SL9800_20 | NADH dehydrogenase [ubiquinone] 1 beta subcomplex subunit 8, mitochondrial | NDUB8 | M2 |
| Q07960 | SL9815_5 | Rho GTPase-activating protein 1 | RHG01 | M2 |
| Q9UKW4 | SL9830_109 | Guanine nucleotide exchange factor VAV3 | VAV3 | M2 |
| P15559 | SL9837_60 | NAD(P)H dehydrogenase [quinone] 1 | NAD(P)H dehydrogenase | M2 |
| P09913 | SL9853_3 | Interferon-induced protein with tetratricopeptide repeats 2 | IFIT2 | M2 |
| P63146 | SL9865_40 | Ubiquitin-conjugating enzyme E2 B | UBE2B | M2 |
| O00757 | SL9867_23 | Fructose-1,6-bisphosphatase isozyme 2 | F16P2 | M2 |
| P26232 | SL9872_23 | Catenin alpha-2 | CTNA2 | M2 |
| P42772 | SL9874_28 | Cyclin-dependent kinase 4 inhibitor B | p15-INK4b | M2 |
| Q9UM54 | SL9894_13 | Unconventional myosin-VI | MYO6 | M2 |
| Q92889 | SL9895_77 | DNA repair endonuclease XPF | XPF | M2 |
| P55316 | SL9896_21 | Forkhead box protein G1 | FOXGB | M2 |
| P12036 | SL9900_36 | Neurofilament heavy polypeptide | NFH | M2 |
| Q8IZW8 | SL9927_96 | Tensin-4 | TENS4 | M2 |
| O75829 | SL9928_125 | Leukocyte cell-derived chemotaxin 1 | LECT1 | M2 |
| O75899 | SL9930_48 | Gamma-aminobutyric acid type B receptor subunit 2 | GABR2 | M2 |
| Q8TBB1 | SL9936_27 | E3 ubiquitin-protein ligase LNX | LNX1 | M2 |
| Q9Y5F3 | SL9941_70 | Protocadherin beta-1 | PCDB1 | M2 |
| Q9UKA2 | SL9951_36 | F-box/LRR-repeat protein 4 | FBXL4 | M2 |
| Q92617 | SL9954_2 | Nuclear pore complex-interacting protein family member B3 | NPPL3 | M2 |
| Q8N7C7 | SL9955_40 | RING finger protein 148 | RN148 | M2 |
| Q9Y508 | SL9957_9 | E3 ubiquitin-protein ligase RNF114 | RN114 | M2 |
| Q9UK28 | SL9959_60 | Transmembrane protein 59-like | TM59L | M2 |
| Q9HBB8 | SL9962_1 | Cadherin-related family member 5 | MUCDL | M2 |
| P0CAP1 | SL9964_10 | Myocardial zonula adherens protein | MYZAP | M2 |
| A2RU67 | SL9981_18 | Protein FAM234B | K1467 | M2 |
| Q96EC8 | SL9984_12 | Protein YIPF6 | YIPF6 | M2 |
| O43296 | SL9993_11 | Zinc finger protein 264 | ZN264 | M2 |
| P29459 | SL10367_62 | Interleukin-12 | IL-12 | M2 |
| P07357 | SL2429_27 | Complement component C8 | C8 | M2 |
| O75462 | SL2607_54 | Cytokine receptor-like factor 1:Cardiotrophin-like cytokine factor 1 Complex | CLF-1/CLC Complex | M2 |
| O95390 | SL2765_4 | Growth/differentiation factor 11/8 | GDF-11/8 | M2 |
| P02671 | SL2796_62 | Fibrinogen | Fibrinogen | M2 |
| Q8NEV9 | SL2829_19 | Interleukin-27 | IL-27 | M2 |
| P01215 | SL2953_31 | Luteinizing hormone | Luteinizing hormone | M2 |
| P01215 | SL3032_11 | Follicle stimulating hormone | FSH | M2 |
| P24941 | SL3357_67 | Cyclin-dependent kinase 2:Cyclin-A2 complex | CDK2/cyclin A | M2 |
| Q00535 | SL3358_51 | Cyclin-dependent kinase 5:Cyclin-dependent kinase 5 activator 1 complex | CDK5/p35 | M2 |
| P01374 | SL3505_6 | Lymphotoxin alpha1:beta2 | Lymphotoxin a1/b2 | M2 |
| P01374 | SL3506_49 | Lymphotoxin alpha2:beta1 | Lymphotoxin a2/b1 | M2 |
| P01215 | SL3521_16 | Thyroid Stimulating Hormone | TSH | M2 |
| P12277 | SL3714_49 | Creatine kinase M-type:Creatine kinase B-type heterodimer | CK-MB | M2 |
| P01031 | SL4482_66 | Complement C5b-C6 complex | C5b, 6 Complex | M2 |
| P02671 | SL4907_56 | D-dimer | D-dimer | M2 |
| P01215 | SL4914_10 | Human Chorionic Gonadotropin | HCG | M2 |
| O14793 | SL2765_4 | Growth/differentiation factor 11/8 | GDF-11/8 | M2 |
| P01225 | SL3032_11 | Follicle stimulating hormone | FSH | M2 |
| P14635 | SL3422_4 | Cyclin-dependent kinase 1:G2/mitotic-specific cyclin-B1 complex | CDK1/cyclin B | M2 |
| P06732 | SL3714_49 | Creatine kinase M-type:Creatine kinase B-type heterodimer | CK-MB | M2 |
| P13671 | SL4482_66 | Complement C5b-C6 complex | C5b, 6 Complex | M2 |
| P63098 | SL4903_72 | Calcineurin | Calcineurin | M2 |
| P18084 | SL4917_62 | Integrin alpha-V: beta-5 complex | Integrin aVb5 | M2 |
| P02679 | SL2796_62 | Fibrinogen | Fibrinogen | M2 |
| P02679 | SL4907_56 | D-dimer | D-dimer | M2 |
| Q6ZMJ2 | SL10419_1 | Scavenger receptor class A member 5 | SCAR5 | M3 |
| P04843 | SL10490_3 | Dolichyl-diphosphooligosaccharide--protein glycosyltransferase subunit 1 | RPN1 | M3 |
| P12111 | SL10511_10 | Collagen alpha-3(VI) chain | Collagen alpha-3(VI) | M3 |
| P32927 | SL10512_13 | Cytokine receptor common subunit beta | IL3RB | M3 |
| Q9Y2W7 | SL10513_13 | Calsenilin | CSEN | M3 |
| P41222 | SL10514_5 | Prostaglandin-H2 D-isomerase | PGD2 synthase | M3 |
| Q9BRK3 | SL10521_10 | Matrix-remodeling-associated protein 8 | MXRA8 | M3 |
| P55001 | SL10569_28 | Microfibrillar-associated protein 2 | MFAP2 | M3 |
| P61769 | SL10574_10 | Beta-2-microglobulin | b2-Microglobulin | M3 |
| Q2UY09 | SL10702_1 | Collagen alpha-1(XXVIII) chain | COSA1 | M3 |
| Q16401 | SL10716_35 | 26S proteasome non-ATPase regulatory subunit 5 | PSMD5 | M3 |
| Q9UBX8 | SL10832_24 | Beta-1,4-galactosyltransferase 6 | B4GT6 | M3 |
| Q13018 | SL10916_44 | Secretory phospholipase A2 receptor | PLA2R | M3 |
| Q8WVF2 | SL10977_55 | Unique cartilage matrix-associated protein | UCMA | M3 |
| P36222 | SL11104_13 | Chitinase-3-like protein 1 | YKL-40 | M3 |
| Q4LDE5 | SL11109_56 | Sushi, von Willebrand factor type A, EGF and pentraxin domain-containing protein 1 | SVEP1 | M3 |
| P32927 | SL11137_43 | Cytokine receptor common subunit beta | IL3RB | M3 |
| P02452 | SL11140_56 | Collagen alpha-1(I) chain | CO1A1 | M3 |
| O95841 | SL11142_11 | Angiopoietin-related protein 1 | ANGL1 | M3 |
| Q4LDE5 | SL11178_21 | Sushi, von Willebrand factor type A, EGF and pentraxin domain-containing protein 1 | SVEP1 | M3 |
| Q9GZM7 | SL11192_168 | Tubulointerstitial nephritis antigen-like | TINAL | M3 |
| P12111 | SL11196_31 | Collagen alpha-3(VI) chain | Collagen alpha-3(VI) | M3 |
| Q9UK23 | SL11208_15 | N-acetylglucosamine-1-phosphodiester alpha-N-acetylglucosaminidase | NAGPA | M3 |
| Q8TEY5 | SL11308_8 | Cyclic AMP-responsive element-binding protein 3-like protein 4 | CR3L4 | M3 |
| Q8WZ42 | SL11352_42 | Titin | TITIN | M3 |
| Q14508 | SL11388_75 | WAP four-disulfide core domain protein 2 | HE4 | M3 |
| Q16352 | SL11436_6 | Alpha-internexin | AINX | M3 |
| O43251 | SL11462_8 | RNA binding protein fox-1 homolog 2 | RBM9 | M3 |
| Q8TDQ0 | SL11481_25 | Hepatitis A virus cellular receptor 2 | TIMD3 | M3 |
| P13987 | SL11514_196 | CD59 glycoprotein | CD59 | M3 |
| Q8IZN3 | SL11677_17 | Probable palmitoyltransferase ZDHHC14 | ZDH14 | M3 |
| P31025 | SL11708_2 | Lipocalin-1 | LCN1 | M3 |
| Q13790 | SL12370_30 | Apolipoprotein F | Apo F | M3 |
| O95704 | SL12784_10 | Amyloid beta A4 precursor protein-binding family B member 3 | APBB3 | M3 |
| Q8NDI1 | SL12813_18 | EH domain-binding protein 1 | EHBP1 | M3 |
| Q14995 | SL12885_42 | Nuclear receptor subfamily 1 group D member 2 | NR1D2 | M3 |
| Q9BUZ4 | SL13041_47 | TNF receptor-associated factor 4 | TRAF4 | M3 |
| Q9BXY4 | SL13094_75 | R-spondin-3 | RSPO3 | M3 |
| Q7Z3B1 | SL13109_82 | Neuronal growth regulator 1 | NEGR1 | M3 |
| Q12841 | SL13112_179 | Follistatin-related protein 1 | FSTL1 | M3 |
| P51884 | SL13114_50 | Lumican | Lumican | M3 |
| Q9H4F8 | SL13118_5 | SPARC-related modular calcium-binding protein 1 | SMOC1 | M3 |
| O43155 | SL13122_19 | Leucine-rich repeat transmembrane protein FLRT2 | FLRT2 | M3 |
| Q6UXK2 | SL13124_20 | Immunoglobulin superfamily containing leucine-rich repeat protein 2 | ISLR2 | M3 |
| Q02487 | SL13126_52 | Desmocollin-2 | DSC2 | M3 |
| Q8N2S1 | SL13133_73 | Latent-transforming growth factor beta-binding protein 4 | LTBP4 | M3 |
| P15291 | SL13381_49 | Beta-1,4-galactosyltransferase 1 | B4GT1 | M3 |
| P29120 | SL13388_57 | Neuroendocrine convertase 1 | NEC1 | M3 |
| Q9NR34 | SL13427_66 | Mannosyl-oligosaccharide 1,2-alpha-mannosidase IC | MA1C1 | M3 |
| P02452 | SL13484_69 | Collagen alpha-1(I) chain | CO1A1 | M3 |
| P00451 | SL13499_30 | Coagulation Factor VIII | Coagulation Factor VIII | M3 |
| Q96MM7 | SL13524_25 | Heparan-sulfate 6-O-sulfotransferase 2 | H6ST2 | M3 |
| O95704 | SL13589_10 | Amyloid beta A4 precursor protein-binding family B member 3 | APBB3 | M3 |
| P22607 | SL13669_6 | Fibroblast growth factor receptor 3 | FGFR-3 | M3 |
| P07333 | SL13682_47 | Macrophage colony-stimulating factor 1 receptor | M-CSF R | M3 |
| P08476 | SL13738_8 | Inhibin beta A chain | Inhibin bA chain | M3 |
| Q92765 | SL13740_51 | Secreted frizzled-related protein 3 | sFRP-3 | M3 |
| P26951 | SL13744_37 | Interleukin-3 receptor subunit alpha | IL-3 Ra | M3 |
| Q07011 | SL14025_18 | Tumor necrosis factor receptor superfamily member 9 | 4-1BB | M3 |
| Q9NPH3 | SL14048_7 | Interleukin-1 Receptor accessory protein | IL-1 R AcP | M3 |
| Q13261 | SL14054_17 | Interleukin-15 receptor subunit alpha | IL-15 Ra | M3 |
| P24592 | SL14088_38 | Insulin-like growth factor-binding protein 6 | IGFBP-6 | M3 |
| P26992 | SL14101_2 | Ciliary neurotrophic factor receptor subunit alpha | CNTFR alpha | M3 |
| Q969Z4 | SL14112_40 | Tumor necrosis factor receptor superfamily member 19L | RELT | M3 |
| Q8IYJ0 | SL14114_18 | PILR alpha-associated neural protein | PIANP | M3 |
| O43921 | SL14124_6 | Ephrin-A2 | Ephrin-A2 | M3 |
| P01574 | SL14127_240 | Interferon beta | IFN-b | M3 |
| P01566 | SL14128_121 | Interferon alpha-10 | IFN10 | M3 |
| P27930 | SL14133_93 | Interleukin-1 receptor type 2 | IL-1 sRII | M3 |
| P52797 | SL14153_8 | Ephrin-A3 | Ephrin-A3 | M3 |
| Q16667 | SL14178_18 | Cyclin-dependent kinase inhibitor 3 | CDKN3 | M3 |
| O14793 | SL14583_49 | Growth/differentiation factor 8 | Myostatin | M3 |
| P52758 | SL14636_25 | Ribonuclease UK114 | Ribonuclease UK114 | M3 |
| O75462 | SL14747_9 | Cytokine receptor-like factor 1 | CRLF1 | M3 |
| Q2TAL6 | SL15308_108 | Brorin | VWC2 | M3 |
| P15086 | SL15375_49 | Carboxypeptidase B | Carboxypeptidase B1 | M3 |
| P15090 | SL15386_7 | Fatty acid-binding protein, adipocyte | FABPA | M3 |
| O60462 | SL15387_44 | Neuropilin-2 | Neuropilin-2 | M3 |
| Q8IZJ1 | SL15394_79 | Netrin receptor UNC5B | UNC5B | M3 |
| Q01995 | SL15640_54 | Transgelin | TAGL | M3 |
| P11717 | SL16057_6 | Cation-independent mannose-6-phosphate receptor | IGF-II receptor | M3 |
| Q8WU39 | SL16322_10 | Marginal zone B- and B1-cell-specific protein | PACAP | M3 |
| P16860 | SL16751_15 | Natriuretic peptides B | BNP | M3 |
| Q8N6G6 | SL16890_37 | ADAMTS-like protein 1 | ATL1 | M3 |
| P08263 | SL17138_8 | Glutathione S-transferase A1 | GST A1-1 | M3 |
| Q16663 | SL18289_16 | C-C motif chemokine 15 | MIP-5 | M3 |
| O95881 | SL19334_62 | Thioredoxin domain-containing protein 12 | TXD12 | M3 |
| Q9Y661 | SL19370_30 | Heparan sulfate glucosamine 3-O-sulfotransferase 4 | HS3S4 | M3 |
| P15692 | SL19437_61 | Isoform L-VEGF165 | L-VEGF165 | M3 |
| P08476 | SL19622_7 | Activin A | Activin A | M3 |
| P39060 | SL2201_17 | Endostatin | Endostatin | M3 |
| Q9H2A7 | SL2436_49 | C-X-C motif chemokine 16 | CXCL16, soluble | M3 |
| O00451 | SL2515_14 | GDNF family receptor alpha-2 | GFRa-2 | M3 |
| P01189 | SL2558_51 | Beta-endorphin | b-Endorphin | M3 |
| P05156 | SL2567_5 | Complement factor I | Factor I | M3 |
| Q01973 | SL2590_69 | Inactive tyrosine-protein kinase transmembrane receptor ROR1 | ROR1 | M3 |
| P15692 | SL2597_8 | Vascular endothelial growth factor A | VEGF | M3 |
| O15123 | SL2602_2 | Angiopoietin-2 | Angiopoietin-2 | M3 |
| P01034 | SL2609_59 | Cystatin-C | Cystatin C | M3 |
| P52798 | SL2614_28 | Ephrin-A4 | Ephrin-A4 | M3 |
| P40189 | SL2620_4 | Interleukin-6 receptor subunit beta | gp130, soluble | M3 |
| Q03405 | SL2652_15 | Urokinase plasminogen activator surface receptor | suPAR | M3 |
| P19438 | SL2654_19 | Tumor necrosis factor receptor superfamily member 1A | TNF sR-I | M3 |
| Q02223 | SL2665_26 | Tumor necrosis factor receptor superfamily member 17 | BCMA | M3 |
| Q16674 | SL2687_2 | Melanoma-derived growth regulatory protein | MIA | M3 |
| P05543 | SL2706_69 | Thyroxine-binding globulin | Thyroxine-Binding Globulin | M3 |
| Q9NP95 | SL2763_66 | Fibroblast growth factor 20 | FGF-20 | M3 |
| P08833 | SL2771_35 | Insulin-like growth factor-binding protein 1 | IGFBP-1 | M3 |
| P09237 | SL2789_26 | Matrilysin | MMP-7 | M3 |
| P33151 | SL2819_23 | Cadherin-5 | Cadherin-5 | M3 |
| O60259 | SL2834_54 | Kallikrein-8 | kallikrein 8 | M3 |
| P08581 | SL2837_3 | Hepatocyte growth factor receptor | Met | M3 |
| P35590 | SL2844_53 | Tyrosine-protein kinase receptor Tie-1, soluble | sTie-1 | M3 |
| P55773 | SL2913_1 | C-C motif chemokine 23 | MPIF-1 | M3 |
| Q96F46 | SL2992_59 | Interleukin-17 receptor A | IL-17 sR | M3 |
| Q13449 | SL2999_6 | Limbic system-associated membrane protein | LSAMP | M3 |
| O14931 | SL3003_29 | Natural cytotoxicity triggering receptor 3 | NKp30 | M3 |
| Q03167 | SL3009_3 | Transforming growth factor beta receptor type 3 | TGF-b R III | M3 |
| P55773 | SL3028_36 | Ck-beta-8-1 | Ck-b-8-1 | M3 |
| Q9NNX6 | SL3029_52 | CD209 antigen | DC-SIGN | M3 |
| P55774 | SL3044_3 | C-C motif chemokine 18 | PARC | M3 |
| P21246 | SL3045_72 | Pleiotrophin | PTN | M3 |
| Q9HD89 | SL3046_31 | Resistin | resistin | M3 |
| P07477 | SL3049_61 | Trypsin-1 | Trypsin | M3 |
| P24394 | SL3055_54 | Interleukin-4 receptor subunit alpha | IL-4 sR | M3 |
| P02748 | SL3060_43 | Complement component C9 | C9 | M3 |
| O95998 | SL3073_51 | Interleukin-18-binding protein | IL-18 BPa | M3 |
| P00742 | SL3077_66 | Coagulation factor Xa | Coagulation Factor Xa | M3 |
| Q9HAV5 | SL3083_71 | Tumor necrosis factor receptor superfamily member 27 | XEDAR | M3 |
| P20333 | SL3152_57 | Tumor necrosis factor receptor superfamily member 1B | TNF sR-II | M3 |
| Q02083 | SL3173_49 | N-acylethanolamine-hydrolyzing acid amidase | ASAHL | M3 |
| P06681 | SL3186_2 | Complement C2 | C2 | M3 |
| Q8N474 | SL3221_54 | Secreted frizzled-related protein 1 | SARP-2 | M3 |
| Q76M96 | SL3234_23 | Coiled-coil domain-containing protein 80 | URB | M3 |
| Q8TEU8 | SL3235_50 | WAP, Kazal, immunoglobulin, Kunitz and NTR domain-containing protein 2 | WFKN2 | M3 |
| Q15582 | SL3283_21 | Transforming growth factor-beta-induced protein ig-h3 | BGH3 | M3 |
| P06734 | SL3291_30 | Low affinity immunoglobulin epsilon Fc receptor | CD23 | M3 |
| Q02246 | SL3296_92 | Contactin-2 | CNTN2 | M3 |
| Q8IWV2 | SL3298_52 | Contactin-4 | Contactin-4 | M3 |
| P31994 | SL3310_62 | Low affinity immunoglobulin gamma Fc region receptor II-b | FCG2B | M3 |
| O75015 | SL3311_27 | Low affinity immunoglobulin gamma Fc region receptor III-B | FCG3B | M3 |
| P56159 | SL3314_74 | GDNF family receptor alpha-1 | GFRa-1 | M3 |
| Q14114 | SL3323_37 | Low-density lipoprotein receptor-related protein 8 | LRP8 | M3 |
| O00339 | SL3325_2 | Matrilin-2 | MATN2 | M3 |
| Q6NW40 | SL3331_8 | RGM domain family member B | RGMB | M3 |
| P10646 | SL3336_50 | Tissue factor pathway inhibitor | TFPI | M3 |
| P35443 | SL3340_53 | Thrombospondin-4 | TSP4 | M3 |
| P23280 | SL3352_80 | Carbonic anhydrase 6 | Carbonic anhydrase 6 | M3 |
| Q9BU40 | SL3362_61 | Chordin-like protein 1 | CRDL1 | M3 |
| P00797 | SL3396_54 | Renin | Renin | M3 |
| P20231 | SL3403_1 | Tryptase beta-2 | TPSB2 | M3 |
| P21815 | SL3415_61 | Bone sialoprotein 2 | BSP | M3 |
| O95633 | SL3438_10 | Follistatin-related protein 3 | FSTL3 | M3 |
| Q13261 | SL3445_53 | Interleukin-15 receptor subunit alpha | IL-15 Ra | M3 |
| P06213 | SL3448_13 | Insulin receptor | IR | M3 |
| Q15063 | SL3457_57 | Periostin | Periostin | M3 |
| P16581 | SL3470_1 | E-selectin | sE-Selectin | M3 |
| P61769 | SL3485_28 | Beta-2-microglobulin | b2-Microglobulin | M3 |
| O43927 | SL3487_32 | C-X-C motif chemokine 13 | BLC | M3 |
| P81172 | SL3504_58 | Hepcidin | LEAP-1 | M3 |
| P48061 | SL3516_60 | Stromal cell-derived factor 1 | SDF-1 | M3 |
| Q15848 | SL3554_24 | Adiponectin | Adiponectin | M3 |
| Q13231 | SL3600_2 | Chitotriosidase-1 | Chitotriosidase-1 | M3 |
| Q9UBP4 | SL3607_71 | Dickkopf-related protein 3 | DKK3 | M3 |
| P16860 | SL3723_1 | Brain natriuretic peptide 32 | BNP-32 | M3 |
| P10082 | SL3727_35 | Peptide YY | PYY | M3 |
| P43489 | SL3730_81 | Tumor necrosis factor receptor superfamily member 4 | TNR4 | M3 |
| Q02763 | SL3773_15 | Angiopoietin-1 receptor, soluble | sTie-2 | M3 |
| P28325 | SL3803_10 | Cystatin-D | CYTD | M3 |
| P21802 | SL3808_76 | Fibroblast growth factor receptor 2 | FGFR-2 | M3 |
| P22607 | SL3809_1 | Fibroblast growth factor receptor 3 | FGFR-3 | M3 |
| Q99665 | SL3815_14 | Interleukin-12 receptor subunit beta-2 | IL-12 RB2 | M3 |
| Q13882 | SL3832_51 | Protein-tyrosine kinase 6 | PTK6 | M3 |
| Q15109 | SL4125_52 | Advanced glycosylation end product-specific receptor, soluble | sRAGE | M3 |
| P00751 | SL4129_72 | Complement factor B | Factor B | M3 |
| P02778 | SL4141_79 | C-X-C motif chemokine 10 | IP-10 | M3 |
| P20783 | SL4145_58 | Neurotrophin-3 | Neurotrophin-3 | M3 |
| Q13219 | SL4148_49 | Pappalysin-1 | PAPP-A | M3 |
| P00749 | SL4158_54 | Urokinase-type plasminogen activator | uPA | M3 |
| P08069 | SL4232_19 | Insulin-like growth factor 1 receptor | IGF-I sR | M3 |
| Q01638 | SL4234_8 | Interleukin-1 receptor-like 1 | IL-1 R4 | M3 |
| P32004 | SL4246_40 | Neural cell adhesion molecule L1 | NCAM-L1 | M3 |
| Q9HCB6 | SL4297_62 | Spondin-1 | Spondin-1 | M3 |
| P02741 | SL4337_49 | C-reactive protein | CRP | M3 |
| Q99988 | SL4374_45 | Growth/differentiation factor 15 | MIC-1 | M3 |
| Q8IU54 | SL4396_54 | Interferon lambda-1 | IFN-lambda 1 | M3 |
| P03973 | SL4413_3 | Antileukoproteinase | SLPI | M3 |
| Q9BZZ2 | SL4464_10 | Sialoadhesin | Sialoadhesin | M3 |
| Q7LFX5 | SL4469_78 | Carbohydrate sulfotransferase 15 | ST4S6 | M3 |
| P13591 | SL4498_62 | Neural cell adhesion molecule 1, 120 kDa isoform | NCAM-120 | M3 |
| Q9GZN4 | SL4534_10 | Brain-specific serine protease 4 | BSSP4 | M3 |
| Q4KMG0 | SL4541_49 | Cell adhesion molecule-related/down-regulated by oncogenes | CDON | M3 |
| Q9UHX3 | SL4546_27 | Adhesion G protein-coupled receptor E2 | EMR2 | M3 |
| Q07654 | SL4721_54 | Trefoil factor 3 | TFF3 | M3 |
| P22079 | SL4801_13 | Lactoperoxidase | PERL | M3 |
| P14151 | SL4831_4 | L-Selectin | sL-Selectin | M3 |
| P29317 | SL4834_61 | Ephrin type-A receptor 2 | Epithelial cell kinase | M3 |
| P09919 | SL4840_73 | Granulocyte colony-stimulating factor | G-CSF | M3 |
| Q16620 | SL4866_59 | BDNF/NT-3 growth factors receptor | TrkB | M3 |
| P15692 | SL4867_15 | Vascular endothelial growth factor A, isoform 121 | VEGF121 | M3 |
| P00742 | SL4878_3 | Coagulation Factor X | Coagulation Factor X | M3 |
| P01189 | SL4890_10 | Corticotropin | ACTH | M3 |
| O15467 | SL4913_78 | C-C motif chemokine 16 | HCC-4 | M3 |
| P45452 | SL4925_54 | Collagenase 3 | MMP-13 | M3 |
| P04278 | SL4929_55 | Sex hormone-binding globulin | SHBG | M3 |
| Q49AH0 | SL4962_52 | Cerebral dopamine neurotrophic factor | ARMEL | M3 |
| Q07507 | SL4979_34 | Dermatopontin | DERM | M3 |
| P19957 | SL4982_54 | Elafin | Elafin | M3 |
| P07359 | SL4990_87 | Platelet glycoprotein Ib alpha chain | GP1BA | M3 |
| Q12884 | SL5029_3 | Prolyl endopeptidase FAP | SEPR | M3 |
| P07478 | SL5034_79 | Trypsin-2 | Trypsin 2 | M3 |
| P04818 | SL5035_7 | Thymidylate synthase | TS | M3 |
| Q01151 | SL5065_8 | CD83 antigen | CD83 | M3 |
| Q08708 | SL5066_134 | CMRF35-like molecule 6 | CLM6 | M3 |
| O15197 | SL5078_82 | Ephrin type-B receptor 6 | EphB6 | M3 |
| P78504 | SL5092_51 | Protein jagged-1 | JAG1 | M3 |
| Q86YT9 | SL5094_62 | Junctional adhesion molecule-like | JAML1 | M3 |
| Q8TD46 | SL5103_30 | Cell surface glycoprotein CD200 receptor 1 | MO2R1 | M3 |
| P46531 | SL5107_7 | Neurogenic locus notch homolog protein 1 | Notch 1 | M3 |
| Q92823 | SL5109_24 | Neuronal cell adhesion molecule | Nr-CAM | M3 |
| P58400 | SL5110_84 | Neurexin-1-beta | NRX1B | M3 |
| Q9HDB5 | SL5111_15 | Neurexin-3-beta | NRX3B | M3 |
| P41217 | SL5112_73 | OX-2 membrane glycoprotein | OX2G | M3 |
| Q9HCK4 | SL5116_62 | Roundabout homolog 2 | ROBO2 | M3 |
| Q9NS68 | SL5131_15 | Tumor necrosis factor receptor superfamily member 19 | TAJ | M3 |
| Q8TDQ0 | SL5134_52 | Hepatitis A virus cellular receptor 2 | TIMD3 | M3 |
| O95185 | SL5139_32 | Netrin receptor UNC5C | UNC5H3 | M3 |
| Q8NBP7 | SL5231_79 | Proprotein convertase subtilisin/kexin type 9 | PCSK9 | M3 |
| O00548 | SL5349_69 | Delta-like protein 1 | DLL1 | M3 |
| Q99983 | SL5358_3 | Osteomodulin | OMD | M3 |
| O15041 | SL5363_51 | Semaphorin-3E | Semaphorin 3E | M3 |
| P25445 | SL5392_73 | Tumor necrosis factor receptor superfamily member 6 | Fas, soluble | M3 |
| P48357 | SL5400_52 | Leptin receptor, soluble | sLeptin R | M3 |
| O75509 | SL5404_53 | Tumor necrosis factor receptor superfamily member 21 | DR6 | M3 |
| P05413 | SL5437_63 | Fatty acid-binding protein, heart | FABP | M3 |
| Q13740 | SL5451_1 | CD166 antigen | ALCAM | M3 |
| P01037 | SL5459_33 | Cystatin-SN | CYTN | M3 |
| P54826 | SL5463_22 | Growth arrest-specific protein 1 | GAS1 | M3 |
| O60243 | SL5465_32 | Heparan-sulfate 6-O-sulfotransferase 1 | H6ST1 | M3 |
| P11362 | SL5532_53 | Fibroblast growth factor receptor 1 | bFGF-R | M3 |
| O14763 | SL5534_49 | Tumor necrosis factor receptor superfamily member 10B | TRAIL R2 | M3 |
| O14786 | SL5542_22 | Neuropilin-1 | NRP1 | M3 |
| Q9H772 | SL5598_3 | Gremlin-2 | GREM2 | M3 |
| Q7Z5A7 | SL5609_92 | Protein FAM19A5 | F19A5 | M3 |
| P58499 | SL5618_50 | Protein FAM3B | FAM3B | M3 |
| Q9NS62 | SL5621_64 | Thrombospondin type-1 domain-containing protein 1 | THSD1 | M3 |
| P12872 | SL5631_83 | Promotilin | MOTI | M3 |
| Q9Y2I2 | SL5637_81 | Netrin-G1 | NTNG1 | M3 |
| P34096 | SL5644_60 | Ribonuclease 4 | RNAS4 | M3 |
| Q93091 | SL5646_20 | Ribonuclease K6 | RNAS6 | M3 |
| P21128 | SL5656_53 | Poly(U)-specific endoribonuclease | PP11 | M3 |
| Q9NPH6 | SL5680_54 | Odorant-binding protein 2b | OBP2B | M3 |
| P22105 | SL5698_60 | Tenascin-X | Tenascin-X | M3 |
| Q96EU7 | SL5735_54 | C1GALT1-specific chaperone 1 | C1GLC | M3 |
| Q92854 | SL5737_61 | Semaphorin-4D | SEM4D | M3 |
| Q9BUN1 | SL5744_12 | Protein MENT | CA056 | M3 |
| P13385 | SL5810_25 | Teratocarcinoma-derived growth factor 1 | Cripto | M3 |
| P42702 | SL5837_49 | Leukemia inhibitory factor receptor | LIF sR | M3 |
| Q99435 | SL6022_57 | Protein kinase C-binding protein NELL2 | NELL2 | M3 |
| P24387 | SL6039_24 | Corticotropin-releasing factor-binding protein | CRHBP | M3 |
| Q9NZK5 | SL6077_63 | Adenosine deaminase CECR1 | CECR1 | M3 |
| Q6WN34 | SL6086_15 | Chordin-like protein 2 | CRDL2 | M3 |
| Q9BRR6 | SL6221_1 | ADP-dependent glucokinase | ADPGK | M3 |
| Q6UXI7 | SL6234_74 | Vitrin | VITRN | M3 |
| Q9BXJ1 | SL6304_8 | Complement C1q tumor necrosis factor-related protein 1 | C1QT1 | M3 |
| Q8NAT1 | SL6359_50 | Protein O-linked-mannose beta-1,4-N-acetylglucosaminyltransferase 2 | AGO61 | M3 |
| Q9BX59 | SL6364_7 | Tapasin-related protein | TPSNR | M3 |
| P80370 | SL6373_54 | Protein delta homolog 1 | DLK1 | M3 |
| Q86TH1 | SL6379_62 | ADAMTS-like protein 2 | ATL2 | M3 |
| Q6PCB0 | SL6385_63 | von Willebrand factor A domain-containing protein 1 | VWA1 | M3 |
| O00515 | SL6407_63 | Ladinin-1 | LAD1 | M3 |
| P22792 | SL6415_90 | Carboxypeptidase N subunit 2 | CPN2 | M3 |
| Q9BXN1 | SL6451_64 | Asporin | ASPN | M3 |
| P04843 | SL6458_6 | Dolichyl-diphosphooligosaccharide--protein glycosyltransferase subunit 1 | RPN1 | M3 |
| Q99727 | SL6462_12 | Metalloproteinase inhibitor 4 | TIMP-4 | M3 |
| A6NGN9 | SL6478_2 | IgLON family member 5 | IGLO5 | M3 |
| P80370 | SL6496_60 | Protein delta homolog 1 | DLK1 | M3 |
| Q9Y4K0 | SL6504_65 | Lysyl oxidase homolog 2 | Lysyl oxidase-like protein 2 | M3 |
| P49755 | SL6506_54 | Transmembrane emp24 domain-containing protein 10 | TMEDA | M3 |
| Q9UL16 | SL6553_68 | Cilia- and flagella-associated protein 45 | CCD19 | M3 |
| Q8TF66 | SL6557_50 | Leucine-rich repeat-containing protein 15 | LRC15 | M3 |
| O60667 | SL6574_11 | Fas apoptotic inhibitory molecule 3 | FAIM3 | M3 |
| P0CG48 | SL6647_55 | PolyUbiquitin K63-linked | PolyUbiquitin K63 | M3 |
| O95631 | SL6649_51 | Netrin-1 | NET1 | M3 |
| O43300 | SL6904_14 | Leucine-rich repeat transmembrane neuronal protein 2 | LRRT2 | M3 |
| Q10469 | SL6909_40 | Alpha-1,6-mannosyl-glycoprotein 2-beta-N-acetylglucosaminyltransferase | MGAT2 | M3 |
| P52848 | SL6927_7 | Bifunctional heparan sulfate N-deacetylase/N-sulfotransferase 1 | NDST1 | M3 |
| Q9Y274 | SL6947_4 | Type 2 lactosamine alpha-2,3-sialyltransferase | SIA10 | M3 |
| Q7Z3B1 | SL7050_5 | Neuronal growth regulator 1 | NEGR1 | M3 |
| Q8WYQ4 | SL7073_69 | Uncharacterized protein C22orf15 | CV015 | M3 |
| O95460 | SL7083_74 | Matrilin-4 | MATN4 | M3 |
| P08217 | SL7140_1 | Chymotrypsin-like elastase family member 2A | ELA2A | M3 |
| Q9BQD7 | SL7148_42 | Protein FAM173A | F173A | M3 |
| O94856 | SL7179_69 | Neurofascin | NFASC | M3 |
| Q8IU57 | SL7192_37 | Interferon lambda receptor 1 | CRF2-12 | M3 |
| O75063 | SL7198_197 | Glycosaminoglycan xylosylkinase | FA20B | M3 |
| P14415 | SL7218_87 | Sodium/potassium-transporting ATPase subunit beta-2 | AT1B2 | M3 |
| Q969X2 | SL7228_2 | Alpha-N-acetylgalactosaminide alpha-2,6-sialyltransferase 6 | SIA7F | M3 |
| Q9H665 | SL7244_16 | IGF-like family receptor 1 | TM149 | M3 |
| Q12907 | SL7638_30 | Vesicular integral-membrane protein VIP36 | Lectin, mannose-binding 2 | M3 |
| P16860 | SL7655_11 | N-terminal pro-BNP | N-terminal pro-BNP | M3 |
| P78504 | SL7754_11 | Protein jagged-1 | JAG1 | M3 |
| Q9Y259 | SL7761_125 | Choline/ethanolamine kinase | CHKB | M3 |
| Q8IZJ1 | SL7776_20 | Netrin receptor UNC5B | UNC5B | M3 |
| P56851 | SL7782_34 | Epididymal secretory protein E3-beta | EP3B | M3 |
| Q9BXJ0 | SL7810_20 | Complement C1q tumor necrosis factor-related protein 5 | C1QTNF5 | M3 |
| Q9C098 | SL7826_1 | Serine/threonine-protein kinase DCLK3 | DCLK3 | M3 |
| Q01974 | SL7861_9 | Tyrosine-protein kinase transmembrane receptor ROR2 | ROR2 | M3 |
| Q24JP5 | SL7871_16 | Transmembrane protein 132A | T132A | M3 |
| P04745 | SL7918_114 | Alpha-amylase 1 | Amylase, alpha 1A | M3 |
| Q86VR8 | SL7921_65 | Four-jointed box protein 1 | FJX1 | M3 |
| Q9H2E6 | SL7945_10 | Semaphorin-6A | Semaphorin-6A | M3 |
| Q8WXD2 | SL7957_2 | Secretogranin-3 | SCG3 | M3 |
| Q13508 | SL7970_315 | Ecto-ADP-ribosyltransferase 3 | NAR3 | M3 |
| Q9NY97 | SL7980_72 | N-acetyllactosaminide beta-1,3-N-acetylglucosaminyltransferase 2 | B3GN2 | M3 |
| Q9NXW2 | SL8006_12 | DnaJ homolog subfamily B member 12 | DJB12 | M3 |
| Q6ZNF0 | SL8011_96 | Iron/zinc purple acid phosphatase-like protein | PAPL | M3 |
| Q5SRI9 | SL8014_359 | Glycoprotein endo-alpha-1,2-mannosidase | MANEA | M3 |
| A6NNA5 | SL8034_6 | Dorsal root ganglia homeobox protein | DRGX | M3 |
| P49747 | SL8043_153 | Cartilage oligomeric matrix protein | COMP | M3 |
| O14511 | SL8060_7 | Pro-neuregulin-2, membrane-bound isoform | NRG2 | M3 |
| Q6P995 | SL8061_102 | Protein FAM171B | F171B | M3 |
| Q8N140 | SL8079_39 | EP300-interacting inhibitor of differentiation 3 | EID3 | M3 |
| Q9BUD6 | SL8099_42 | Spondin-2 | SPON2 | M3 |
| P29323 | SL8225_86 | Ephrin type-B receptor 2 | EPHB2 | M3 |
| Q12913 | SL8250_2 | Receptor-type tyrosine-protein phosphatase eta | PTPRJ | M3 |
| Q5VY43 | SL8275_31 | Platelet endothelial aggregation receptor 1 | PEAR1 | M3 |
| P18509 | SL8285_64 | Pituitary adenylate cyclase-activating polypeptide | PACA | M3 |
| Q14956 | SL8289_8 | Transmembrane glycoprotein NMB | GPNMB | M3 |
| O00300 | SL8304_50 | Tumor necrosis factor receptor superfamily member 11B | OPG | M3 |
| Q96DA0 | SL8310_6 | Zymogen granule protein 16 homolog B | U773 | M3 |
| Q07654 | SL8323_163 | Trefoil factor 3 | TFF3 | M3 |
| Q6IA17 | SL8326_63 | Single Ig IL-1-related receptor | SIGIRR | M3 |
| P20333 | SL8368_102 | Tumor necrosis factor receptor superfamily member 1B | TNF sR-II | M3 |
| P80370 | SL8380_244 | Protein delta homolog 1 | DLK1 | M3 |
| Q9BXY4 | SL8427_118 | R-spondin-3 | RSPO3 | M3 |
| P18509 | SL8446_4 | Pituitary adenylate cyclase-activating polypeptide 27 | PACAP-27 | M3 |
| P18509 | SL8450_36 | Pituitary adenylate cyclase-activating polypeptide 38 | PACAP-38 | M3 |
| P41159 | SL8484_24 | Leptin | Leptin | M3 |
| O14763 | SL8529_1 | Tumor necrosis factor receptor superfamily member 10B | TRAIL R2 | M3 |
| Q9P0T7 | SL8597_1 | Transmembrane protein 9 | TMEM9 | M3 |
| Q14956 | SL8606_39 | Transmembrane glycoprotein NMB | GPNMB | M3 |
| Q9UJ90 | SL8756_41 | Potassium voltage-gated channel subfamily E regulatory beta subunit 5 | KCE1L | M3 |
| Q9NT22 | SL8773_172 | EMILIN-3 | EMIL3 | M3 |
| Q6P995 | SL8786_6 | Protein FAM171B | F171B | M3 |
| Q7Z7K6 | SL8817_29 | Centromere protein V | CENPV | M3 |
| Q9NXS2 | SL8866_53 | Glutaminyl-peptide cyclotransferase-like protein | QPCTL | M3 |
| Q8IZS8 | SL8885_6 | Voltage-dependent calcium channel subunit alpha-2/delta-3 | CA2D3 | M3 |
| Q5VY43 | SL8892_14 | Platelet endothelial aggregation receptor 1 | PEAR1 | M3 |
| Q92859 | SL8900_28 | Neogenin | NEO1 | M3 |
| Q9UJ90 | SL8908_14 | Potassium voltage-gated channel subfamily E regulatory beta subunit 5 | KCE1L | M3 |
| Q9NYX4 | SL8924_55 | Neuron-specific vesicular protein calcyon | CALY | M3 |
| Q96GP6 | SL8956_96 | Scavenger receptor class F member 2 | SREC-II | M3 |
| Q96DZ1 | SL8957_72 | Endoplasmic reticulum lectin 1 | XTP3B | M3 |
| O00533 | SL8958_51 | Neural cell adhesion molecule L1-like protein | CHL1 | M3 |
| Q6P179 | SL8960_3 | Endoplasmic reticulum aminopeptidase 2 | LRAP | M3 |
| P39059 | SL8974_172 | Collagen alpha-1(XV) chain | COFA1 | M3 |
| P49746 | SL8982_65 | Thrombospondin-3 | TSP3 | M3 |
| Q9UHN6 | SL8992_1 | Transmembrane protein 2 | TMEM2 | M3 |
| Q9Y661 | SL8998_15 | Heparan sulfate glucosamine 3-O-sulfotransferase 4 | HS3S4 | M3 |
| Q8NFZ4 | SL9027_10 | Neuroligin-2 | NLGN2 | M3 |
| O60476 | SL9077_10 | Mannosyl-oligosaccharide 1,2-alpha-mannosidase IB | MA1A2 | M3 |
| O95841 | SL9092_33 | Angiopoietin-related protein 1 | ANGL1 | M3 |
| Q16552 | SL9170_24 | Interleukin-17A | IL-17 | M3 |
| P58499 | SL9177_6 | Protein FAM3B | FAM3B | M3 |
| P01189 | SL9204_33 | Pro-opiomelanocortin | Corticotropin-lipotropin | M3 |
| P36955 | SL9211_19 | Pigment epithelium-derived factor | PEDF | M3 |
| Q9UBX1 | SL9212_22 | Cathepsin F | CATF | M3 |
| O15031 | SL9216_100 | Plexin-B2 | PLXB2 | M3 |
| P48307 | SL9233_71 | Tissue factor pathway inhibitor 2 | TFPI -2 | M3 |
| Q9P0T7 | SL9249_17 | Transmembrane protein 9 | TMEM9 | M3 |
| P16442 | SL9253_52 | Histo-blood group ABO system transferase | BGAT | M3 |
| Q15818 | SL9256_78 | Neuronal pentraxin-1 | NPTX1 | M3 |
| Q9NP99 | SL9266_1 | Triggering receptor expressed on myeloid cells 1 | sTREM-1 | M3 |
| P48061 | SL9278_9 | Stromal cell-derived factor 1 | SDF-1 | M3 |
| P16562 | SL9282_12 | Cysteine-rich secretory protein 2 | CRIS2 | M3 |
| P35270 | SL9287_6 | Sepiapterin reductase | SPRE | M3 |
| P55001 | SL9294_45 | Microfibrillar-associated protein 2 | MFAP2 | M3 |
| P23435 | SL9313_27 | Cerebellin-1 | CBLN1 | M3 |
| Q9HC57 | SL9316_67 | WAP four-disulfide core domain protein 1 | WFDC1 | M3 |
| Q96DN0 | SL9333_59 | Endoplasmic reticulum resident protein 27 | ERP27 | M3 |
| Q9Y5I4 | SL9361_7 | Protocadherin alpha-C2 | PCDC2 | M3 |
| Q9HBL6 | SL9368_64 | Leucine-rich repeat and transmembrane domain-containing protein 1 | LRTM1 | M3 |
| Q9BX93 | SL9380_2 | Group XIIB secretory phospholipase A2-like protein | sPLA(2)-XIII | M3 |
| P25445 | SL9459_7 | Tumor necrosis factor receptor superfamily member 6 | Fas, soluble | M3 |
| Q12907 | SL9468_8 | Vesicular integral-membrane protein VIP36 | Lectin, mannose-binding 2 | M3 |
| Q14126 | SL9484_75 | Desmoglein-2 | Desmoglein-2 | M3 |
| O95831 | SL9522_3 | Apoptosis-inducing factor 1, mitochondrial | AIF | M3 |
| Q9H8J5 | SL9557_5 | MANSC domain-containing protein 1 | MANS1 | M3 |
| A6NHS7 | SL9578_263 | MANSC domain-containing protein 4 | MANS4 | M3 |
| O60909 | SL9595_11 | Beta-1,4-galactosyltransferase 2 | B4GT2 | M3 |
| Q8IYJ0 | SL9599_6 | PILR alpha-associated neural protein | PIANP | M3 |
| Q8NFZ4 | SL9772_153 | Neuroligin-2 | NLGN2 | M3 |
| Q8TDY8 | SL9793_145 | Immunoglobulin superfamily DCC subclass member 4 | IGDC4 | M3 |
| Q06520 | SL9829_91 | Bile salt sulfotransferase | SULT 2A1 | M3 |
| P09972 | SL9876_20 | Fructose-bisphosphate aldolase C | aldolase C | M3 |
| Q96GP6 | SL9925_56 | Scavenger receptor class F member 2 | SREC-II | M3 |
| P01876 | SL11089_7 | Immunoglobulin A | IgA | M3 |
| P08476 | SL18814_21 | Inhibin beta A chain:Inhibin beta C chain heterodimer | Activin AC | M3 |
| O75462 | SL2607_54 | Cytokine receptor-like factor 1:Cardiotrophin-like cytokine factor 1 Complex | CLF-1/CLC Complex | M3 |
| P02794 | SL5934_1 | Ferritin | Ferritin | M3 |
| P08476 | SL8467_9 | Inhibin beta A chain:Inhibin beta B chain heterodimer | Activin AB | M3 |
| O14793 | SL2765_4 | Growth/differentiation factor 11/8 | GDF-11/8 | M3 |
| O00189 | SL10076_1 | AP-4 complex subunit mu-1 | AP4M1 | M4 |
| P01106 | SL10362_35 | Myc proto-oncogene protein | c-Myc | M4 |
| Q9ULT6 | SL10390_21 | E3 ubiquitin-protein ligase ZNRF3 | ZNRF3 | M4 |
| Q9Y5C1 | SL10391_1 | Angiopoietin-related protein 3 | ANGL3 | M4 |
| O43561 | SL10551_7 | Linker for activation of T-cells family member 1 | LAT | M4 |
| Q8NC67 | SL10562_42 | Neuropilin and tolloid-like protein 2 | NETO2 | M4 |
| Q6UXG2 | SL10637_50 | UPF0577 protein KIAA1324 | K1324 | M4 |
| Q6UWU4 | SL10885_36 | Bombesin receptor-activated protein C6orf89 | CF089 | M4 |
| O43752 | SL10945_11 | Syntaxin-6 | Syntaxin-6 | M4 |
| Q8IV08 | SL10948_14 | Phospholipase D3 | PLD3 | M4 |
| Q4V9L6 | SL11110_4 | Transmembrane protein 119 | TM119 | M4 |
| Q9H9V4 | SL11160_56 | RING finger protein 122 | RN122 | M4 |
| Q7Z6M3 | SL11173_29 | Allergin-1 | MILR1 | M4 |
| P21757 | SL11207_3 | Macrophage scavenger receptor types I and II | Macrophage scavenger receptor | M4 |
| P04424 | SL11241_8 | Argininosuccinate lyase | ARLY | M4 |
| O75427 | SL11252_30 | Leucine-rich repeat and calponin homology domain-containing protein 4 | LRCH4 | M4 |
| Q8TC36 | SL11260_47 | SUN domain-containing protein 5 | SPA4L | M4 |
| Q9NZR2 | SL11275_94 | Low-density lipoprotein receptor-related protein 1B | LRP1B | M4 |
| Q6GTX8 | SL11284_24 | Leukocyte-associated immunoglobulin-like receptor 1 | LAIR1 | M4 |
| P48730 | SL11289_31 | Casein kinase I isoform delta | KC1D | M4 |
| P49959 | SL11319_106 | Double-strand break repair protein MRE11 | MRE11 | M4 |
| O00472 | SL11494_4 | RNA polymerase II elongation factor ELL2 | ELL2 | M4 |
| O95822 | SL11538_216 | Malonyl-CoA decarboxylase, mitochondrial | DCMC | M4 |
| Q96C24 | SL11563_51 | Synaptotagmin-like protein 4 | SYTL4 | M4 |
| O43663 | SL11591_43 | Protein regulator of cytokinesis 1 | PRC1 | M4 |
| P13674 | SL11645_9 | Prolyl 4-hydroxylase subunit alpha-1 | P4HA1 | M4 |
| Q9UI08 | SL11656_110 | Ena/VASP-like protein | EVL | M4 |
| Q6S5L8 | SL11692_21 | SHC-transforming protein 4 | SHC4 | M4 |
| Q9NT99 | SL11911_13 | Leucine-rich repeat-containing protein 4B | LRC4B | M4 |
| Q8IVU1 | SL11952_1 | Immunoglobulin superfamily DCC subclass member 3 | IGDC3 | M4 |
| Q8N766 | SL11989_35 | ER membrane protein complex subunit 1 | EMC1 | M4 |
| Q03393 | SL12014_19 | 6-pyruvoyl tetrahydrobiopterin synthase | PTPS | M4 |
| O95786 | SL12382_2 | Probable ATP-dependent RNA helicase DDX58 | DDX58 | M4 |
| O60869 | SL12415_122 | Endothelial differentiation-related factor 1 | EDF1 | M4 |
| Q9P016 | SL12424_107 | Thymocyte nuclear protein 1 | THYN1 | M4 |
| Q9H074 | SL12430_78 | Polyadenylate-binding protein-interacting protein 1 | PAIP1 | M4 |
| O00482 | SL12444_39 | Nuclear receptor subfamily 5 group A member 2 | NR5A2 | M4 |
| Q9HB21 | SL12459_13 | Pleckstrin homology domain-containing family A member 1 | PKHA1 | M4 |
| O95793 | SL12471_47 | Double-stranded RNA-binding protein Staufen homolog 1 | STAU1 | M4 |
| P10827 | SL12527_50 | Thyroid hormone receptor alpha | THA | M4 |
| Q9HB90 | SL12548_75 | Ras-related GTP-binding protein C | RRAGC | M4 |
| Q14145 | SL12568_14 | Kelch-like ECH-associated protein 1 | KEAP1 | M4 |
| P43357 | SL12576_21 | Melanoma-associated antigen 3 | MAGE-3 | M4 |
| O95352 | SL12627_97 | Ubiquitin-like modifier-activating enzyme ATG7 | ATG7 | M4 |
| P18440 | SL12632_14 | Arylamine N-acetyltransferase 1 | ARY1 | M4 |
| P43378 | SL12633_3 | Tyrosine-protein phosphatase non-receptor type 9 | PTN9 | M4 |
| O75815 | SL12634_79 | Breast cancer anti-estrogen resistance protein 3 | BCAR3 | M4 |
| O14717 | SL12635_9 | tRNA (cytosine(38)-C(5))-methyltransferase | TRDMT | M4 |
| Q9BYW2 | SL12647_52 | Histone-lysine N-methyltransferase SETD2 | SETD2 | M4 |
| O60671 | SL12670_15 | Cell cycle checkpoint protein RAD1 | RAD1 | M4 |
| Q9UK80 | SL12681_63 | Ubiquitin carboxyl-terminal hydrolase 21 | UBP21 | M4 |
| Q53G59 | SL12695_62 | Kelch-like protein 12 | KLH12 | M4 |
| P53396 | SL12700_9 | ATP-citrate synthase | ACLY | M4 |
| Q9HAT8 | SL12702_13 | E3 ubiquitin-protein ligase pellino homolog 2 | PELI2 | M4 |
| Q8TDX7 | SL12703_6 | Serine/threonine-protein kinase Nek7 | NEK7 | M4 |
| Q15751 | SL12705_9 | Probable E3 ubiquitin-protein ligase HERC1 | HERC1 | M4 |
| O14777 | SL12730_3 | Kinetochore protein NDC80 homolog | KNTC2 | M4 |
| O75534 | SL12735_39 | Cold shock domain-containing protein E1 | CSDE1 | M4 |
| Q9Y2I1 | SL12738_43 | Nischarin | NISCH | M4 |
| Q58F21 | SL12748_6 | Bromodomain testis-specific protein | BRDT | M4 |
| Q92870 | SL12753_6 | Amyloid beta A4 precursor protein-binding family B member 2 | APBB2 | M4 |
| Q92870 | SL12761_12 | Amyloid beta A4 precursor protein-binding family B member 2 | APBB2 | M4 |
| Q96ER9 | SL12790_10 | Coiled-coil domain-containing protein 51 | CCD51 | M4 |
| Q96MN2 | SL12794_6 | NACHT, LRR and PYD domains-containing protein 4 | NALP4 | M4 |
| P55040 | SL12817_1 | GTP-binding protein GEM | GEM | M4 |
| Q9UKA1 | SL12846_3 | F-box/LRR-repeat protein 5 | FBXL5 | M4 |
| P51808 | SL12867_40 | Dynein light chain Tctex-type 3 | DYLT3 | M4 |
| P28749 | SL12879_5 | Retinoblastoma-like protein 1 | p107 | M4 |
| O75452 | SL12881_17 | Retinol dehydrogenase 16 | RDH16 | M4 |
| P43353 | SL12940_35 | Aldehyde dehydrogenase family 3 member B1 | AL3B1 | M4 |
| Q9UN76 | SL13053_6 | Sodium- and chloride-dependent neutral and basic amino acid transporter B(0+) | S6A14 | M4 |
| P43220 | SL13085_18 | Glucagon-like peptide 1 receptor | GLP1R | M4 |
| Q16665 | SL13089_6 | Hypoxia-inducible factor 1-alpha | HIF-1a | M4 |
| Q9NZU0 | SL13123_3 | Leucine-rich repeat transmembrane protein FLRT3 | FLRT3 | M4 |
| P01861 | SL13231_90 | Ig gamma-4, Kappa | IgG4, Kappa | M4 |
| Q13336 | SL13430_50 | Urea transporter 1 | UT1 | M4 |
| Q8N912 | SL13464_8 | Nutritionally-regulated adipose and cardiac enriched protein homolog | NRAC | M4 |
| Q96K49 | SL13485_20 | Transmembrane protein 87B | TM87B | M4 |
| Q9NZW5 | SL13490_1 | MAGUK p55 subfamily member 6 | MPP6 | M4 |
| Q8N5B7 | SL13494_6 | Ceramide synthase 5 | CERS5 | M4 |
| Q93084 | SL13510_7 | Sarcoplasmic/endoplasmic reticulum calcium ATPase 3 | AT2A3 | M4 |
| P05455 | SL13526_5 | Lupus La protein | LA | M4 |
| Q8N699 | SL13541_1 | Myc target protein 1 | MYCT1 | M4 |
| P55036 | SL13568_30 | 26S proteasome non-ATPase regulatory subunit 4 | PSMD4 | M4 |
| O94929 | SL13578_98 | Actin-binding LIM protein 3 | ABLM3 | M4 |
| P05455 | SL13625_19 | Lupus La protein | LA | M4 |
| Q14449 | SL13628_58 | Growth factor receptor-bound protein 14 | GRB14 | M4 |
| Q15291 | SL13631_1 | Retinoblastoma-binding protein 5 | RB binding protein 5 | M4 |
| P21695 | SL13697_51 | Glycerol-3-phosphate dehydrogenase [NAD(+)], cytoplasmic | GPDA | M4 |
| Q96IW7 | SL13713_164 | Vesicle-trafficking protein SEC22a | SC22A | M4 |
| P80075 | SL13748_4 | C-C motif chemokine 8 | MCP-2 | M4 |
| O15068 | SL13934_3 | Guanine nucleotide exchange factor DBS | MCF2L | M4 |
| P07205 | SL13936_24 | Phosphoglycerate kinase 2 | PGK2 | M4 |
| O75899 | SL13948_50 | Gamma-aminobutyric acid type B receptor subunit 2 | GABR2 | M4 |
| Q9BPX1 | SL13972_4 | 17-beta-hydroxysteroid dehydrogenase 14 | DHB14 | M4 |
| Q08257 | SL13983_27 | Quinone oxidoreductase | QOR | M4 |
| O43252 | SL14007_22 | Bifunctional 3'-phosphoadenosine 5'-phosphosulfate synthase 1 | PAPS1 | M4 |
| Q96C10 | SL14012_17 | Probable ATP-dependent RNA helicase DHX58 | LGP2 | M4 |
| Q9UPW8 | SL14052_26 | Protein unc-13 homolog A | UN13A | M4 |
| Q15811 | SL14070_56 | Intersectin-1 | ITSN1 | M4 |
| Q9ULT6 | SL14122_132 | E3 ubiquitin-protein ligase ZNRF3 | ZNRF3 | M4 |
| P01574 | SL14127_240 | Interferon beta | IFN-b | M4 |
| Q9HBX9 | SL14135_3 | Relaxin receptor 1 | RXFP1 | M4 |
| Q9P0K8 | SL14204_55 | Forkhead box protein J2 | FOXJ2 | M4 |
| Q9BY84 | SL14631_22 | Dual specificity protein phosphatase 16 | DUS16 | M4 |
| P12757 | SL14670_1 | Ski-like protein | SKIL | M4 |
| Q8TDF5 | SL15298_199 | Neuropilin and tolloid-like protein 1 | NETO1 | M4 |
| O43663 | SL15310_61 | Protein regulator of cytokinesis 1 | PRC1 | M4 |
| Q9NZV1 | SL15492_1 | Cysteine-rich motor neuron 1 protein | CRIM1 | M4 |
| P21757 | SL15533_97 | Macrophage scavenger receptor types I and II | Macrophage scavenger receptor | M4 |
| P11912 | SL15674_3 | B-cell antigen receptor complex-associated protein alpha chain | CD79A | M4 |
| P37023 | SL16318_12 | Serine/threonine-protein kinase receptor R3 | ALK-1 | M4 |
| Q6UWL6 | SL16609_106 | Kin of IRRE-like protein 2 | KIRR2 | M4 |
| P16860 | SL16751_15 | Natriuretic peptides B | BNP | M4 |
| P08571 | SL16914_104 | Monocyte differentiation antigen CD14, soluble | sCD14 | M4 |
| P49888 | SL16932_5 | Estrogen sulfotransferase | SULT 1E | M4 |
| P02768 | SL18380_78 | Serum albumin | Albumin | M4 |
| P15692 | SL19437_61 | Isoform L-VEGF165 | L-VEGF165 | M4 |
| Q9Y4X3 | SL2192_63 | C-C motif chemokine 27 | CTACK | M4 |
| P58294 | SL2247_20 | Prokineticin-1 | EG-VEGF | M4 |
| P08311 | SL2431_17 | Cathepsin G | Cathepsin G | M4 |
| Q9NZK7 | SL2447_7 | Group IIE secretory phospholipase A2 | GIIE | M4 |
| O60609 | SL2505_49 | GDNF family receptor alpha-3 | GFRa-3 | M4 |
| P01189 | SL2558_51 | Beta-endorphin | b-Endorphin | M4 |
| P15692 | SL2597_8 | Vascular endothelial growth factor A | VEGF | M4 |
| Q12904 | SL2714_78 | Endothelial monocyte-activating polypeptide 2 | EMAP-2 | M4 |
| Q9GZX6 | SL2778_10 | Interleukin-22 | IL-22 | M4 |
| P04114 | SL2797_56 | Apolipoprotein B | Apo B | M4 |
| Q16619 | SL2889_37 | Cardiotrophin-1 | Cardiotrophin-1 | M4 |
| P04070 | SL2961_1 | Vitamin K-dependent protein C | Protein C | M4 |
| P57087 | SL2997_8 | Junctional adhesion molecule B | JAM-B | M4 |
| P05162 | SL3033_57 | Galectin-2 | Galectin-2 | M4 |
| P14136 | SL3034_1 | Glial fibrillary acidic protein | GFAP | M4 |
| P09486 | SL3043_49 | SPARC | ON | M4 |
| P12034 | SL3065_65 | Fibroblast growth factor 5 | FGF-5 | M4 |
| Q9BZM6 | SL3081_70 | NKG2D ligand 1 | ULBP-1 | M4 |
| Q9NR28 | SL3122_6 | Diablo homolog, mitochondrial | SMAC | M4 |
| P01730 | SL3143_3 | T-cell surface glycoprotein CD4 | sCD4 | M4 |
| P54108 | SL3187_52 | Cysteine-rich secretory protein 3 | CRIS3 | M4 |
| P16112 | SL3280_49 | Aggrecan core protein | Aggrecan | M4 |
| P12314 | SL3312_64 | High affinity immunoglobulin gamma Fc receptor I | FCGR1 | M4 |
| Q9HB63 | SL3327_27 | Netrin-4 | NET4 | M4 |
| P42684 | SL3342_76 | Abelson tyrosine-protein kinase 2 | ABL2 | M4 |
| Q16610 | SL3366_51 | Extracellular matrix protein 1 | ECM1 | M4 |
| P20718 | SL3373_5 | Granzyme H | Granzyme H | M4 |
| P23946 | SL3423_59 | Chymase | Chymase | M4 |
| P02751 | SL3434_34 | Fibronectin Fragment 3 | FN1.3 | M4 |
| P02751 | SL3435_53 | Fibronectin Fragment 4 | FN1.4 | M4 |
| P36888 | SL3437_80 | Receptor-type tyrosine-protein kinase FLT3 | Flt-3 | M4 |
| P06239 | SL3452_17 | Tyrosine-protein kinase Lck | LCK | M4 |
| O00626 | SL3508_78 | C-C motif chemokine 22 | MDC | M4 |
| Q14012 | SL3592_4 | Calcium/calmodulin-dependent protein kinase type 1 | CAMK1 | M4 |
| Q8N6P7 | SL3620_67 | Interleukin-22 receptor subunit alpha-1 | IL22RA1 | M4 |
| P16860 | SL3723_1 | Brain natriuretic peptide 32 | BNP-32 | M4 |
| P04070 | SL3758_68 | Activated Protein C | Activated Protein C | M4 |
| P02751 | SL4131_72 | Fibronectin | Fibronectin | M4 |
| P47992 | SL4143_74 | Lymphotactin | Lymphotactin | M4 |
| O60506 | SL4224_7 | Heterogeneous nuclear ribonucleoprotein Q | HNRPQ | M4 |
| P27169 | SL4261_55 | Serum paraoxonase/arylesterase 1 | paraoxonase 1 | M4 |
| P25787 | SL4280_47 | Proteasome subunit alpha type-2 | PSA2 | M4 |
| Q07817 | SL4423_77 | Bcl-2-like protein 1 | BCL2-like 1 protein | M4 |
| Q8IZU9 | SL4557_61 | Kin of IRRE-like protein 3 | KIRR3 | M4 |
| Q16719 | SL4559_64 | Kynureninase | KYNU | M4 |
| P06239 | SL4560_34 | Tyrosine-protein kinase Lck | LCK | M4 |
| Q6UXX9 | SL4566_24 | R-spondin-2 | RSPO2 | M4 |
| P01374 | SL4703_87 | Lymphotoxin-alpha | TNF-b | M4 |
| P15692 | SL4867_15 | Vascular endothelial growth factor A, isoform 121 | VEGF121 | M4 |
| P01189 | SL4890_10 | Corticotropin | ACTH | M4 |
| P06576 | SL4965_27 | ATP synthase subunit beta, mitochondrial | ATP synthase beta chain | M4 |
| P38919 | SL4997_19 | Eukaryotic initiation factor 4A-III | IF4A3 | M4 |
| Q9Y219 | SL5093_47 | Protein jagged-2 | JAG2 | M4 |
| Q14108 | SL5100_53 | Lysosome membrane protein 2 | LIMP II | M4 |
| Q13946 | SL5178_5 | High affinity cAMP-specific 3',5'-cyclic phosphodiesterase 7A | PDE7A | M4 |
| P42684 | SL5261_13 | Abelson tyrosine-protein kinase 2 | ABL2 | M4 |
| O75815 | SL5262_57 | Breast cancer anti-estrogen resistance protein 3 | BCAR3 | M4 |
| Q9H1K4 | SL5280_68 | Mitochondrial glutamate carrier 2 | GHC2 | M4 |
| Q5KU26 | SL5457_5 | Collectin-12 | COLEC12 | M4 |
| Q9UNW1 | SL5586_66 | Multiple inositol polyphosphate phosphatase 1 | MINP1 | M4 |
| Q8TDF5 | SL5639_49 | Neuropilin and tolloid-like protein 1 | NETO1 | M4 |
| P81534 | SL5679_16 | Beta-defensin 103 | HBD-3 | M4 |
| Q6PB30 | SL5716_49 | Putative chondrosarcoma-associated gene 1 protein | CSAG1 | M4 |
| Q15884 | SL5719_66 | Protein FAM189A2 | CI061 | M4 |
| Q16568 | SL5743_82 | Cocaine- and amphetamine-regulated transcript protein | CART | M4 |
| Q96L08 | SL5752_63 | Sushi domain-containing protein 3 | SUSD3 | M4 |
| Q08648 | SL5762_35 | Sperm-associated antigen 11B | Sperm-associated antigen 11 | M4 |
| P32970 | SL5807_77 | CD70 antigen | CD70 | M4 |
| Q13561 | SL5879_51 | Dynactin subunit 2 | Dynactin subunit 2 | M4 |
| P61278 | SL5957_30 | Somatostatin-28 | Somatostatin-28 | M4 |
| P12273 | SL6060_2 | Prolactin-inducible protein | PIP | M4 |
| P46734 | SL6151_18 | Dual specificity mitogen-activated protein kinase kinase 3 | MP2K3 | M4 |
| Q5EBL8 | SL6219_14 | PDZ domain-containing protein 11 | PDZ11 | M4 |
| Q6UXI9 | SL6342_10 | Nephronectin | Nephronectin | M4 |
| Q15256 | SL6361_49 | Receptor-type tyrosine-protein phosphatase R | PTPRR | M4 |
| P0C0P6 | SL6390_18 | Neuropeptide S | NPS | M4 |
| Q8NBR0 | SL6400_33 | Tumor protein p53-inducible protein 13 | P5I13 | M4 |
| P11465 | SL6405_74 | Pregnancy-specific beta-1-glycoprotein 2 | PSG2 | M4 |
| Q86Y29 | SL6442_6 | B melanoma antigen 3 | BAGE3 | M4 |
| P83859 | SL6463_59 | Orexigenic neuropeptide QRFP | OX26 | M4 |
| Q96QH8 | SL6494_60 | Sperm acrosome-associated protein 5 | sperm acrosome associated 5 | M4 |
| O75078 | SL6586_19 | Disintegrin and metalloproteinase domain-containing protein 11 | ADA11 | M4 |
| P23352 | SL6603_18 | Anosmin-1 | KALM | M4 |
| P02786 | SL6895_1 | Transferrin receptor protein 1 | TR | M4 |
| Q8N6Q1 | SL6907_17 | Transmembrane and coiled-coil domain-containing protein 5A | TMC5A | M4 |
| P61647 | SL6930_95 | Alpha-2,8-sialyltransferase 8F | SIA8F | M4 |
| Q96FT7 | SL6951_26 | Acid-sensing ion channel 4 | ASIC4 | M4 |
| Q9Y231 | SL6991_24 | Alpha-(1,3)-fucosyltransferase 9 | FUT9 | M4 |
| Q02742 | SL7016_12 | Beta-1,3-galactosyl-O-glycosyl-glycoprotein beta-1,6-N-acetylglucosaminyltransferase | GCNT1 | M4 |
| Q12983 | SL7045_4 | BCL2/adenovirus E1B 19 kDa protein-interacting protein 3 | BNIP3 | M4 |
| Q9UHP7 | SL7054_87 | C-type lectin domain family 2 member D | CLC2D | M4 |
| O95866 | SL7065_1 | Protein G6b | G6B | M4 |
| Q5JX69 | SL7066_199 | Protein FAM209B | F209B | M4 |
| P23276 | SL7070_25 | Kell blood group glycoprotein | KELL | M4 |
| Q9BT88 | SL7089_42 | Synaptotagmin-11 | SYT11 | M4 |
| Q8IVU1 | SL7118_24 | Immunoglobulin superfamily DCC subclass member 3 | IGDC3 | M4 |
| P01570 | SL7180_114 | Interferon alpha-14 | IFN14 | M4 |
| O95292 | SL7181_17 | Vesicle-associated membrane protein-associated protein B/C | VAPB | M4 |
| Q9Y644 | SL7203_125 | Beta-1,3-N-acetylglucosaminyltransferase radical fringe | RFNG | M4 |
| P00387 | SL7215_18 | NADH-cytochrome b5 reductase 3 | NADH-cytochrome b5 reductase | M4 |
| Q99584 | SL7223_60 | Protein S100-A13 | S100A13 | M4 |
| O15547 | SL7233_73 | P2X purinoceptor 6 | P2RX6 | M4 |
| Q8NCH0 | SL7262_191 | Carbohydrate sulfotransferase 14 | CHSTE | M4 |
| P16860 | SL7655_11 | N-terminal pro-BNP | N-terminal pro-BNP | M4 |
| Q96PE5 | SL7736_28 | Opalin | OPALI | M4 |
| Q969F0 | SL7740_33 | Fetal and adult testis-expressed transcript protein | FATE1 | M4 |
| P01906 | SL7757_5 | HLA class II histocompatibility antigen, DQ alpha 2 chain | DQA2 | M4 |
| O43736 | SL7765_15 | Integral membrane protein 2A | ITM2A | M4 |
| Q96LW7 | SL7778_104 | Caspase recruitment domain-containing protein 19 | BINCA | M4 |
| Q13162 | SL7789_182 | Peroxiredoxin-4 | PRDX4 | M4 |
| P15018 | SL7790_21 | Leukemia inhibitory factor | LIF | M4 |
| Q9GZT6 | SL7792_58 | Coiled-coil domain-containing protein 90B, mitochondrial | CC90B | M4 |
| P11912 | SL7796_10 | B-cell antigen receptor complex-associated protein alpha chain | CD79A | M4 |
| Q8N2K0 | SL7825_7 | Monoacylglycerol lipase ABHD12 | ABD12 | M4 |
| O75920 | SL7842_52 | Small EDRK-rich factor 1 | SERF1 | M4 |
| P53816 | SL7865_126 | HRAS-like suppressor 3 | HRSL3 | M4 |
| P60468 | SL7878_2 | Protein transport protein Sec61 subunit beta | SC61B | M4 |
| Q15836 | SL7903_18 | Vesicle-associated membrane protein 3 | VAMP3 | M4 |
| Q13291 | SL7953_20 | Signaling lymphocytic activation molecule | SLAF1 | M4 |
| O60704 | SL8024_64 | Protein-tyrosine sulfotransferase 2 | TPST2 | M4 |
| Q8N5K1 | SL8094_20 | CDGSH iron-sulfur domain-containing protein 2 | CISD2 | M4 |
| Q4G148 | SL8229_1 | Glucoside xylosyltransferase 1 | GXLT1 | M4 |
| P0C7M8 | SL8242_9 | C-type lectin domain family 2 member L | CLC2L | M4 |
| Q8N690 | SL8315_5 | Beta-defensin 119 | DB119 | M4 |
| Q9NWH9 | SL8320_5 | SAFB-like transcription modulator | SLTM | M4 |
| Q6GMR7 | SL8396_42 | Fatty-acid amide hydrolase 2 | FAAH2 | M4 |
| Q6UXX9 | SL8409_3 | R-spondin-2 | RSPO2 | M4 |
| P01241 | SL8462_18 | Somatotropin | HGH | M4 |
| Q12805 | SL8480_29 | EGF-containing fibulin-like extracellular matrix protein 1 | FBLN3 | M4 |
| Q14145 | SL8485_7 | Kelch-like ECH-associated protein 1 | KEAP1 | M4 |
| Q9UKF2 | SL8520_8 | Disintegrin and metalloproteinase domain-containing protein 30 | ADA30 | M4 |
| O95866 | SL8659_68 | Protein G6b | G6B | M4 |
| P35052 | SL8697_38 | Glypican-1 | Glypican 1 | M4 |
| Q9NZV1 | SL8699_43 | Cysteine-rich motor neuron 1 protein | CRIM1 | M4 |
| P35218 | SL8791_151 | Carbonic anhydrase 5A, mitochondrial | Carbonic Anhydrase VA | M4 |
| P02786 | SL8795_48 | Transferrin receptor protein 1 | TR | M4 |
| P27824 | SL8834_58 | Calnexin | Calnexin | M4 |
| Q5R387 | SL8850_5 | Putative inactive group IIC secretory phospholipase A2 | PA2GC | M4 |
| Q9NR55 | SL8858_21 | Basic leucine zipper transcriptional factor ATF-like 3 | BATF3 | M4 |
| Q5EE01 | SL8864_59 | Centromere protein W | CENPW | M4 |
| P60059 | SL8872_1 | Protein transport protein Sec61 subunit gamma | SC61G | M4 |
| Q8N967 | SL8906_60 | Leucine-rich repeat and transmembrane domain-containing protein 2 | LRTM2 | M4 |
| Q9H013 | SL8948_13 | Disintegrin and metalloproteinase domain-containing protein 19 | ADA19 | M4 |
| Q96GP6 | SL8956_96 | Scavenger receptor class F member 2 | SREC-II | M4 |
| P08571 | SL8969_49 | Monocyte differentiation antigen CD14 | CD14 | M4 |
| O43353 | SL8970_9 | Receptor-interacting serine/threonine-protein kinase 2 | RIPK2 | M4 |
| O43353 | SL8993_151 | Receptor-interacting serine/threonine-protein kinase 2 | RIPK2 | M4 |
| Q9BUV0 | SL9050_170 | Arginine/serine-rich protein 1 | CA063 | M4 |
| Q9UPX6 | SL9075_121 | UPF0258 protein KIAA1024 | K1024 | M4 |
| Q5JS37 | SL9087_8 | NHL repeat-containing protein 3 | NHLC3 | M4 |
| Q96C24 | SL9090_9 | Synaptotagmin-like protein 4 | SYTL4 | M4 |
| Q6UXG2 | SL9097_5 | UPF0577 protein KIAA1324 | K1324 | M4 |
| Q5XG99 | SL9106_87 | LysM and putative peptidoglycan-binding domain-containing protein 4 | LYSM4 | M4 |
| A8MVW5 | SL9116_28 | HEPACAM family member 2 | HECA2 | M4 |
| Q9NZU0 | SL9128_34 | Leucine-rich repeat transmembrane protein FLRT3 | FLRT3 | M4 |
| Q9Y258 | SL9168_31 | C-C motif chemokine 26 | Eotaxin-3 | M4 |
| Q16552 | SL9170_24 | Interleukin-17A | IL-17 | M4 |
| P08962 | SL9190_7 | CD63 antigen | CD63 | M4 |
| P01189 | SL9204_33 | Pro-opiomelanocortin | Corticotropin-lipotropin | M4 |
| P40313 | SL9229_9 | Chymotrypsin-like protease CTRL-1 | CTRL | M4 |
| O75629 | SL9357_4 | Protein CREG1 | CREG1 | M4 |
| O00499 | SL9574_11 | Myc box-dependent-interacting protein 1 | BIN1 | M4 |
| Q9H8L6 | SL9723_105 | Multimerin-2 | EMILIN-3 | M4 |
| Q96CS3 | SL9738_7 | FAS-associated factor 2 | FAF2 | M4 |
| Q9NVH1 | SL9783_75 | DnaJ homolog subfamily C member 11 | DJC11 | M4 |
| P00374 | SL9823_2 | Dihydrofolate reductase | DYR | M4 |
| P27707 | SL9836_20 | Deoxycytidine kinase | DCK | M4 |
| Q7Z6E9 | SL9887_40 | E3 ubiquitin-protein ligase RBBP6 | RBBP6 | M4 |
| Q13126 | SL9910_9 | S-methyl-5'-thioadenosine phosphorylase | MTAP | M4 |
| Q9NT99 | SL9916_146 | Leucine-rich repeat-containing protein 4B | LRC4B | M4 |
| P05937 | SL9918_23 | Calbindin | Calbindin D28 | M4 |
| Q96GP6 | SL9925_56 | Scavenger receptor class F member 2 | SREC-II | M4 |
| O75899 | SL9930_48 | Gamma-aminobutyric acid type B receptor subunit 2 | GABR2 | M4 |
| P17302 | SL9937_7 | Gap junction alpha-1 protein | CXA1 | M4 |
| P49336 | SL3359_11 | Cyclin-dependent kinase 8:Cyclin-C complex | CDK8/cyclin C | M4 |
| P42336 | SL3390_72 | PIK3CA/PIK3R1 | PIK3CA/PIK3R1 | M4 |
| P01374 | SL3505_6 | Lymphotoxin alpha1:beta2 | Lymphotoxin a1/b2 | M4 |
| P01374 | SL3506_49 | Lymphotoxin alpha2:beta1 | Lymphotoxin a2/b1 | M4 |
| O43318 | SL5259_2 | Mitogen-activated protein kinase kinase kinase 7:TGF-beta-activated kinase 1 and MAP3K7-binding protein 1 fusion | TAK1-TAB1 | M4 |
| O14791 | SL11510_31 | Apolipoprotein L1 | Apo L1 | M8 |
| P01042 | SL15343_337 | Kininogen, HMW, Two Chain | Kininogen, HMW, Two Chain | M8 |
| P01042 | SL19631_13 | Kininostatin | Kininostatin | M8 |
| P18075 | SL2972_57 | Bone morphogenetic protein 7 | BMP-7 | M8 |
| P60568 | SL3070_1 | Interleukin-2 | IL-2 | M8 |
| P05154 | SL3389_7 | Plasma serine protease inhibitor | PCI | M8 |
| Q04756 | SL3617_80 | Hepatocyte growth factor activator | HGFA | M8 |
| P03952 | SL4152_58 | Plasma kallikrein | Prekallikrein | M8 |
| P26927 | SL4407_10 | Hepatocyte growth factor-like protein | MSP | M8 |
| Q14624 | SL4811_33 | Inter-alpha-trypsin inhibitor heavy chain H4 | ITI heavy chain H4 | M8 |
| P19021 | SL5620_13 | Peptidyl-glycine alpha-amidating monooxygenase | AMD | M8 |
| P01148 | SL5627_53 | Progonadoliberin-1 | GON1 | M8 |
| Q969E1 | SL5708_1 | Liver-expressed antimicrobial peptide 2 | LEAP2 | M8 |
| Q16363 | SL6577_64 | Laminin subunit alpha-4 | LAMA4 | M8 |
| P01042 | SL7784_1 | Kininogen-1 | Kininogen, HMW | M8 |
| O60507 | SL7928_183 | Protein-tyrosine sulfotransferase 1 | TPST1 | M8 |
| Q9H4A9 | SL8327_26 | Dipeptidase 2 | DPEP2 | M8 |
| P01178 | SL8356_88 | Oxytocin-neurophysin 1 | NEU1 | M8 |
| P10645 | SL8476_11 | Chromogranin-A | CgA | M8 |
| Q8IUK5 | SL9235_3 | Plexin domain-containing protein 1 | PXDC1 | M8 |
| O14791 | SL9506_10 | Apolipoprotein L1 | Apo L1 | M8 |
| Q86WK6 | SL9979_13 | Amphoterin-induced protein 1 | AMGO1 | M8 |
| Q8N729 | SL9986_14 | Neuropeptide W | Neuropeptide W | M8 |
