# Supplementary material for: Inflammatory proteins associated with Alzheimer’s disease reduced by a GLP1 receptor agonist: a post hoc analysis of the EXSCEL randomized placebo controlled trial
Source: Alzheimers Res Ther. 2024 Oct 2;16:212. doi: 10.1186/s13195-024-01573-x (PMC11448378; doi:10.1186/s13195-024-01573-x)
Supplement: Supplementary file 3 — Supplementary Table S3 [file 13195_2024_1573_MOESM3_ESM.docx]

Table S3.1. Effects of outlier removal on analyses. We compare results for the Wilcoxon signed rank test for the overall linear mixed models, with and without outliers.

|  |  | OUTLIERS INCLUDED | | OUTLIERS REMOVED | |
| --- | --- | --- | --- | --- | --- |
| SeqID1 | Target | Mean difference | P value | Mean difference | P value |
| SL13717_15_sc | FCN2 | -0.019 | 0.004 | -0.019 | 0.016 |
| SL2925_9_sc | PAI-1 | -0.033 | 0.003 | -0.033 | 0.003 |
| SL2967_8_sc | VCAM-1 | 0.034 | 0.000 | 0.035 | 0.001 |
| SL3313_21_sc | FCN2 | -0.008 | 0.907 | -0.010 | 0.917 |
| SL4337_49_sc | CRP | 0.007 | 0.576 | 0.007 | 0.608 |

Table S3.2. Effects of outlier removal on Model A analyses.

|  |  | Interaction term pvalue | |
| --- | --- | --- | --- |
| SeqID1 | Target | OUTLIERS INCLUDED | OUTLIERS REMOVED |
| SL13717_15_sc | FCN2 | 0.016 | 0.012 |
| SL2925_9_sc | PAI-1 | 0.183 | 0.183 |
| SL2967_8_sc | VCAM-1 | 0.014 | 0.024 |
| SL3313_21_sc | FCN2 | 0.720 | 0.361 |
| SL4337_49_sc | CRP | 0.000 | 4.32E-07 |
