# Supplementary material for: Inflammatory proteins associated with Alzheimer’s disease reduced by a GLP1 receptor agonist: a post hoc analysis of the EXSCEL randomized placebo controlled trial
Source: Alzheimers Res Ther. 2024 Oct 2;16:212. doi: 10.1186/s13195-024-01573-x (PMC11448378; doi:10.1186/s13195-024-01573-x)
Supplement: Supplementary file 4 — Supplementary Table S4 [file 13195_2024_1573_MOESM4_ESM.docx]

**Table S4.** Exploratory analysis of individual proteins included in the four clusters: Top 20 most significant proteins by Model C p values (adjusted for age, sex, smoking, SBP, DBP, BMI, HbA_1c_, cholesterol, HDL-cholesterol, LDL-cholesterol, triglycerides, diabetes duration) for the whole cohort.

|  |  | **Interaction term p value** | |
| --- | --- | --- | --- |
| Target | Module | Nominal | FDR-adjusted |
| LIPR1 | M2 | 2.52E-39 | 4.64E-36 |
| Trypsin 2 | M3 | 5.35E-26 | 2.46E-23 |
| Carboxypeptidase B1 | M3 | 5.87E-23 | 2.49E-20 |
| Trypsin | M3 | 2.16E-20 | 5.96E-18 |
| C1QT1 | M3 | 3.91E-20 | 1.03E-17 |
| SEM3G | M2 | 7.29E-19 | 1.83E-16 |
| PLXB2 | M3 | 4.34E-17 | 9.57E-15 |
| ALCAM | M3 | 1.11E-16 | 2.36E-14 |
| C2 | M3 | 1.93E-15 | 3.44E-13 |
| ELA2A | M3 | 4.00E-15 | 6.89E-13 |
| B3GN2 | M3 | 2.42E-14 | 4.05E-12 |
| NEO1 | M3 | 4.86E-12 | 6.38E-10 |
| CECR1 | M3 | 2.21E-11 | 2.49E-09 |
| C1QR1 | M2 | 2.54E-11 | 2.76E-09 |
| Contactin-4 | M3 | 2.56E-11 | 2.76E-09 |
| Prolylcarboxypeptidase | M2 | 3.16E-11 | 3.29E-09 |
| Cadherin-5 | M3 | 5.74E-11 | 5.54E-09 |
| M-CSF R | M3 | 7.13E-11 | 6.55E-09 |
| Nr-CAM | M3 | 6.86E-11 | 6.41E-09 |
| ROBO2 | M3 | 1.49E-10 | 1.26E-08 |
